# Machine learning guided prediction of warfarin blood levels for personalized medicine based on clinical longitudinal data from cardiac surgery patients: a prospective observational study

**DOI:** 10.1097/JS9.0000000000001734

**Published:** 2024-06-04

**Authors:** Ling Xue, Shan He, Rajeev K. Singla, Qiong Qin, Yinglong Ding, Linsheng Liu, Xiaoliang Ding, Harbil Bediaga-Bañeres, Sonia Arrasate, Aliuska Durado-Sanchez, Yuzhen Zhang, Zhenya Shen, Bairong Shen, Liyan Miao, Humberto González-Díaz

**Affiliations:** a Department of Pharmacy, the First Affiliated Hospital of Soochow University; b Department of Cardiovascular Surgery, the First Affiliated Hospital of Soochow University; c Department of Cardiology, the First Affiliated Hospital of Soochow University; d Institute for Cardiovascular Science, Soochow University; e Institute for Interdisciplinary Drug Research and Translational Sciences, Soochow University; f Joint Laboratory of Artificial Intelligence for Critical Care Medicine, Department of Critical Care Medicine and Institutes for Systems Genetics, Frontiers Science Center for Disease-related Molecular Network, West China Hospital, Sichuan University, Chengdu, China; g Department of Pharmacology, Faculty of Medicine, University of The Basque Country (UPV/EHU), Bilbao, Basque Country; h Department of Organic and Inorganic Chemistry, Faculty of Science and Technology, University of The Basque Country (UPV/EHU), Bilbao, Basque Country, Spain; i IKERDATA S.L., ZITEK, University of The Basque Country (UPV/EHU), Bilbao, Basque Country; j Department of Painting, Faculty of Fine Arts, University of the Basque Country UPV/EHU, 48940, Leioa, Biscay; k Department of Public Law, Faculty of Law, University of The Basque Country (UPV/EHU), Leioa, Biscay, Basque, Country; l BIOFISIKA: Basque Center for Biophysics CSIC, University of The Basque Country (UPV/EHU), Bilbao, Basque Country; m IKERBASQUE, Basque Foundation for Science, Bilbao, Basque Country, Spain; n School of Pharmaceutical Sciences, Lovely Professional University, Phagwara, Punjab, India

**Keywords:** cardiac surgery, information fusion, machine learning, personalized medicine, perturbation theory, warfarin

## Abstract

**Background:**

Warfarin is a common oral anticoagulant, and its effects vary widely among individuals. Numerous dose-prediction algorithms have been reported based on cross-sectional data generated via multiple linear regression or machine learning. This study aimed to construct an information fusion perturbation theory and machine-learning prediction model of warfarin blood levels based on clinical longitudinal data from cardiac surgery patients.

**Methods and material:**

The data of 246 patients were obtained from electronic medical records. Continuous variables were processed by calculating the distance of the raw data with the moving average (MA ∆v_ki_(**s**_j_)), and categorical variables in different attribute groups were processed using Euclidean distance (ED ǁ∆v_k_(**s**_j_)ǁ). Regression and classification analyses were performed on the raw data, MA ∆v_ki_(**s**_j_), and ED ǁ∆v_k_(**s**_j_)ǁ. Different machine-learning algorithms were chosen for the STATISTICA and WEKA software.

**Results:**

The random forest (RF) algorithm was the best for predicting continuous outputs using the raw data. The correlation coefficients of the RF algorithm were 0.978 and 0.595 for the training and validation sets, respectively, and the mean absolute errors were 0.135 and 0.362 for the training and validation sets, respectively. The proportion of ideal predictions of the RF algorithm was 59.0%. General discriminant analysis (GDA) was the best algorithm for predicting the categorical outputs using the MA ∆v_ki_(**s**_j_) data. The GDA algorithm’s total true positive rate (TPR) was 95.4% and 95.6% for the training and validation sets, respectively, with MA ∆v_ki_(**s**_j_) data.

**Conclusions:**

An information fusion perturbation theory and machine-learning model for predicting warfarin blood levels was established. A model based on the RF algorithm could be used to predict the target international normalized ratio (INR), and a model based on the GDA algorithm could be used to predict the probability of being within the target INR range under different clinical scenarios.

## Graphical abstract

**Figure:**
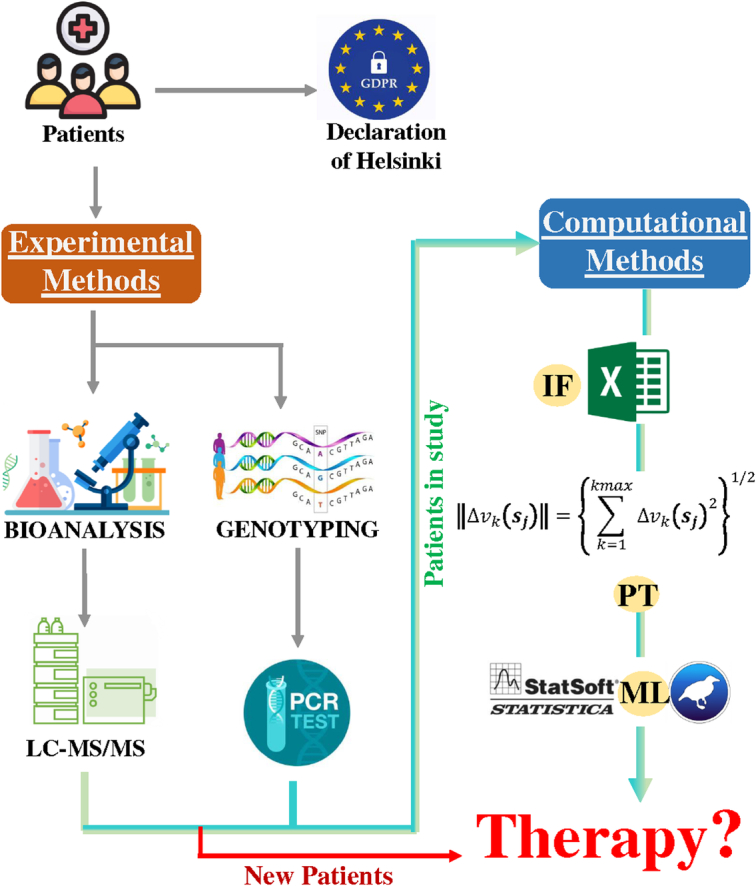

## Introduction

Highlights

- Clinical longitudinal data were recorded from cardiac surgery patients.
- Warfarin blood levels were considered a continuous or categorical output.
- An artificial intelligence model of warfarin blood levels was established.

Warfarin is a commonly prescribed oral anticoagulant in the clinic. The approved clinical indications for warfarin are thromboembolism prophylaxis and treatment for atrial fibrillation (AF), prosthetic heart valve, pulmonary embolism (PE), renal disease, hepatic disease, pregnancy, hip and total knee arthroplasty, malignancy, and other conditions^1^. Moreover, large individual variability in effects and a narrow therapeutic index make warfarin dosage challenging for clinicians^2^. Although several novel oral anticoagulants (NOACs), such as dabigatran, rivaroxaban, apixaban, and edoxaban, have been used in the clinic since 2010, the approved clinical indications for NOACs do not include heart valve replacement (HVR)^1,3^. Warfarin is currently the only anticoagulant approved by the FDA for patients with mechanical valves^3^. Moreover, compared with NOACs, warfarin is more cost-effective^1,4^. Therefore, most patients who need anticoagulant treatment for HVR or in low- and middle-income territories may choose warfarin in the clinic.

Numerous dose-prediction algorithms have been developed based on demographic, clinical, and genetic factors^2^. Most algorithms utilize multiple linear regression (MLR)^5^. MLR assumes that the relationship between the dependent and independent variables is simply linear. All MLR algorithms are restricted to patients within a target international normalized ratio (INR) range^6^. Therefore, these algorithms might not provide accurate guidance for dosages to achieve INRs out of the target range. Several studies have established warfarin dosage algorithms using pharmacometrics (PMX) approaches^7,8^. However, the PMX approach is usually time-consuming and labor-intensive, and it may take several days to weeks to complete a study^9^.

Attempts are being made to apply artificial intelligence (AI) in medicine, including precision medication and drug development, to aid clinical decision-making^10^. In this context, artificial intelligence/machine learning (AI/ML) methods are tools that have the potential to improve the efficiency of pharmacological approaches and reduce experimental time and resources^11^. For instance, several researchers have successfully applied AI/ML algorithms to estimate warfarin doses^12–20^. However, the datasets used by these researchers were obtained mainly from public sources, such as the International Warfarin Pharmacogenetic Consortium (IWPC, www.pharmgkb.org/downloads) and the Chinese Low-Intensity Anticoagulant Therapy After Heart Valve Replacement (CLIATHVR, unpublished) study, and these data concerning these populations were cross-sectional data^21^. Practical management guidance recommends long-term warfarin anticoagulant use for venous thromboembolism therapy for over three months^22,23^. The INRs usually fluctuate during warfarin anticoagulant therapy; therefore, the reported ML models may not be suitable over the whole period of warfarin anticoagulant therapy. This study estimated warfarin blood levels based on clinical longitudinal follow-up data, accounting for the medical data collected *in situ*. AI/ML models could provide personalized medicine (PM) treatments for specific patients. The dataset included all essential patient clinical data, precise demographic characteristics, combined drugs, indications for surgery, comorbidities, and laboratory results. The extent of the dataset created by combining all the patients’ health information makes this study even more challenging. IFPTML combines information fusion (IF) techniques with perturbation theory (PT) concepts and ML algorithms^24^. PT methods can find the solution to a problem by adding perturbations to the previously known exact solution for a related problem^25^. IFPTML modeling is helpful for the IF of big data from different sources^26^. Combining IF, PT and ML allows it to establish models for predicting various response targets^24^. IFPTML has been applied mainly in medicinal chemistry, proteomics, organic synthesis, nanotechnology, and others^24–27^. Here, we applied IFPTML to analyze data from clinical scenarios.

We introduced the IFPTML approach to construct a predictive model for warfarin blood levels. The IFPTML predictive models can assist with patient follow-up, improving patient prognosis, and designing more personalized and effective treatment protocols for warfarin.

## Materials and methods

### Experimental methods

#### Patients

All patients provided written informed consent. Patients underwent cardiac surgery for various reasons, including valvular heart disease, rheumatic valve disease, infectious endocarditis, and aortic dissection. The inclusion criteria for patients were age older than or equal to 18 years and cardiac surgery. The exclusion criteria were as follows: patients who refused surgery had tumors, were pregnant, had severe liver or kidney dysfunction, or had coagulation dysfunction. The indications for warfarin included HVR and large vessel replacement of the pericardium. The demographic characteristics, combined drugs, indications for surgery, comorbidities, and laboratory test results were collected from the patient’s electronic medical records. The hospital clinical laboratory routinely measured the INR, blood cells, and blood biochemistry. Most patients were followed up for approximately three months after the first day of warfarin administration after cardiac surgery. This study was registered on Research Registry website (www.researchregistry.com/browse-the-registry#home/registrationdetails/65f3c161b71a9f00265cab41/) with a unique identifying number (UIN) for researchregistry10088. This study was reported in line with the strengthening the reporting of cohort studies in surgery (STROCSS, Supplemental Digital Content 1, http://links.lww.com/JS9/C688) guidelines^28^.

#### Data protection

All the data analysis procedures in Europe complied with the General Data Protection Regulation (GDPR)^29–32^. The GDPR defines pseudonymization in Article 4 (5) as the processing of personal data in such a way that they can no longer be attributed to a subject without the use of additional information, provided that such additional information is kept separate and is subject to technical and organizational measures intended to ensure that the personal data are not attributed to an identified or identifiable natural person. In general terms, pseudonymization aims to protect personal data by hiding the identity of individuals (data subjects) in a dataset, for example, by replacing one or more personal data identifiers with so-called pseudonyms and adequately protecting the link between the pseudonyms and the initial identifiers. Consequently, all patient data were pseudonymized by the team from the first institution using an internal code to identify the patients. These codes and patients’ identities were never shared with the other researchers in this study. In addition, the pseudonymized data were processed on a single computer and were never copied, transferred, or accessed from/to other devices at the second institution.

#### Bioanalysis

Warfarin exerts its anticoagulation effect by interfering with the recycling of vitamin K in the liver, subsequently blocking the synthesis of vitamin K-dependent clotting factors^33^. Therefore, fluctuations in vitamin K concentrations in the body may affect the anticoagulant effects of warfarin. However, the concentration of vitamin K is not monitored as part of standard clinical care. The present study established a method for measuring the serum vitamin K concentration via liquid chromatography-tandem mass spectrometry (LC-MS/MS). The validation of the measurement method was consistent with the biological sample analysis guidelines in the Chinese Pharmacopoeia. Approximately 2 ml of blood was drawn in a coagulation tube. The vitamin K concentrations were detected in these blood samples. All the samples were stored at −80°C before analysis. Only the concentrations of vitamin K1 and one subtype of vitamin K2 (MK4) were measured.

#### Genotyping

Genetic analysis of *CYP2C9* (rs1057910) and *VKORC1* (rs9923231) was performed in the standard clinical care for patients taking warfarin^8^. Many additional genes have been reported to be involved in the pharmacokinetics (PK) and pharmacodynamics (PD) of warfarin and the coagulation process^34^. Other warfarin-related genes were genotyped by multiplex polymerase chain reaction (PCR) and sequencing. The detailed methods used for multiplex PCR are described in the supplementary materials, Supplemental Digital Content 2, http://links.lww.com/JS9/C689.

### Computational methods

#### Univariate analysis

To avoid introducing additional nonphysiological variables into the models, we first performed univariate analysis to assess the effect of each variable on warfarin blood levels before performing IFPTML data analysis. Each continuous variable was tested for continuous output by simple correlation analysis with the Pearson correlation coefficient and *P* value. Each categorical variable was tested for continuous output by the Mann–Whitney U test or Kruskal–Wallis H test with a *P* value. The Wilcoxon rank sum test tested each continuous variable for categorical output with a *P* value. Pearson’s chi-square test with a *P* value tested each categorical variable for categorical output. A *P* value less than 0.05 was considered to indicate statistical significance. All univariate analyses were performed with R (ver.4.2.3).

#### IFPTML data analysis

**Figure 1:**
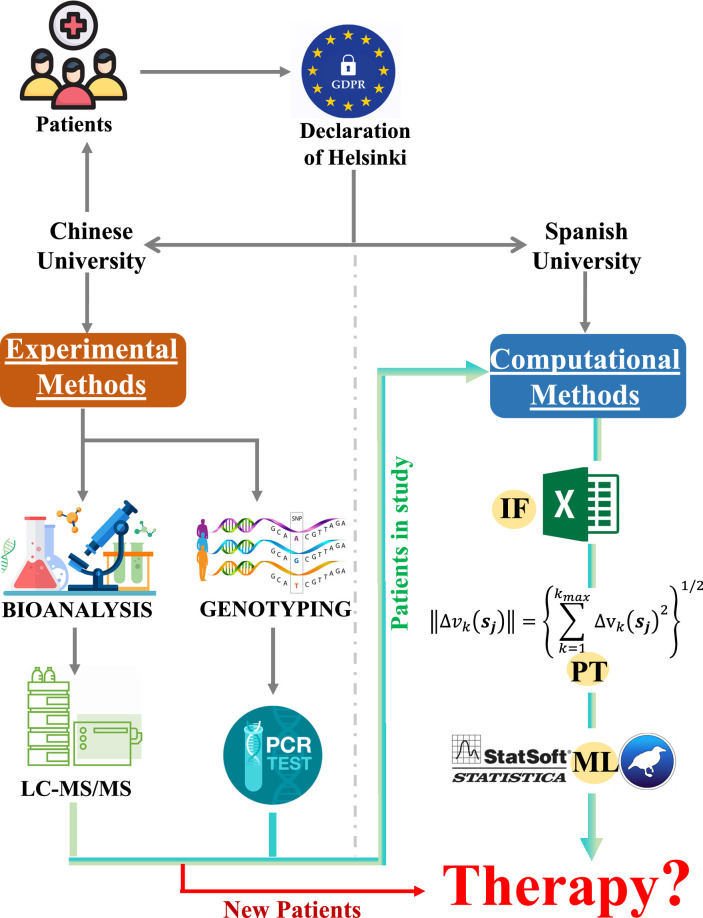
Workflow summary of AI/ML guided prediction of warfarin blood levels for personalized therapy based on clinical longitudinal data from cardiac surgery patients. AI, artificial intelligence; IF, information fusion; LC-MS/MS, liquid chromatography-mass spectrometry/mass spectrometry; ML, machine learning; PCR, polymerase chain reaction; PT, perturbation theory.

In this study, we used the IFPTML approach to train/validate linear and nonlinear models to predict the outcome values v_ij_ for each i^th^ patient conforming to the subset of conditions/labels **s**_j_. The general form of the IFPTML model is illustrated in Equation 1 and Figure 1. We also describe different cases based on the general linear model. This approach includes three stages: IF, PT, and AI/ML.


$${f\left(v_{\mathrm{ij}}\right)}_{\mathrm{calc}}=a_{0}\;+\;a_{1}\cdot \alpha \cdot {f\left(v_{\mathrm{ij}}\right)}_{\mathrm{ref}}\;+\sum_{s=1}^{\mathrm{smax}}a_{c,s}\cdot {\left\{\sum_{k=1}^{\mathrm{kmax}}{a_{k,s}\cdot \left[v_{k}-\alpha \cdot \left(<v_{k}\left(s_{j}\right)>\right)\right]}^{q}\right\}}^{r}\;\left(\mathrm{Eq}.\;1\right)$$

The outcome of equation f(v_ij_)_calc_ is a scoring function of the predicted propensity of the patient to have the clinically accepted level for the output variable v_ij_. The first input variable is the function of reference f(v_ij_)_ref_, which is equal to the prior probability that the j^th^ property of the patient is at the desired level. The variables v_k_ are the continuous input variables for the patient (age, weight, height, drug doses, and others). The variables **s**_j_=[s_1_, s_2_, … s_max_] are partitions/subsets of the patients’ original discrete/labeling variables (genre, mutations, treatments, and others). The parameters of a_1_, a_c,s_, and a_k,s_ are the coefficients of the model to be fitted by AI/ML algorithms. Finally, the user fits the hyperparameters α, q, and r. Here, we introduce three cases/stages of IFPTML linear models depending on the values of the hyperparameters (α, q, and r) and the thresholds of the coefficients (a_1_, a_c,s_, and a_k,s_); the three detailed forms of IFPTML models are shown in the supplementary materials, Supplemental Digital Content 2, http://links.lww.com/JS9/C689. The hyperparameters are: α=switching, q=moment power, and r=distance power. The coefficients are as follows: a_0_=bias, a_1_=variable coefficients, a_c,s_=distance weights, and a_k,s_=moment weights.

**Figure 2:**
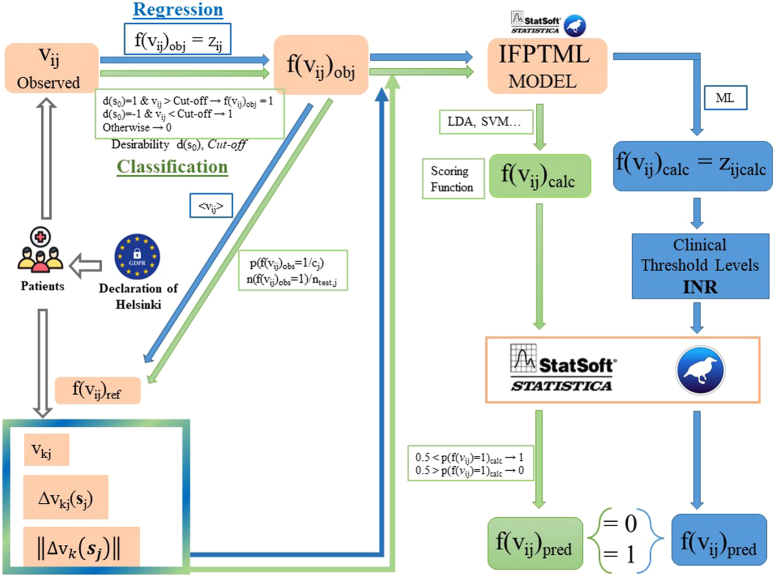
General workflow for pre- and post-processing of variables in IFPTML model development. IFPTML: information fusion perturbation theory machine learning; INR: international normalized ratio; LDA, linear discriminant analysis; ML, machine learning; SVM, support vector machine.

The next step is performing the following IFPTML data preprocessing phases to explore the diverse cases of the IFPTML models mentioned in the previous section. We introduce how to identify the data (data engineering) and determine the values for the objective function f(v_ij_)_obs_, reference function f(v_ij_)_ref_, moving averages <v_k_(**s**_j_)>, deviations ∆v_k_(**s**_j_) (first-order PTOs), and Euclidean distances ǁ∆v_k_(**s**_j_)ǁ. The general workflow for calculating the model’s objective function and input parameters is shown in Figure 2. In the previous cases, linear discriminant analysis (LDA) can be the algorithm of choice for identifying this kind of IFPTML linear classification.

#### IF preprocessing and data rearrangement

First, we performed an IF analysis of patient clinical data, which included demographics, laboratory tests, and genetic data. The database was rearranged as follows: each row (case) relates to the i^th^ patient at the j^th^ visit/interview. The columns contained three types of variables. The first variable class (v_ij_) was each patient’s output values. The second class (v_ki_) included patient data, such as age, weight, and drug dose. The third-class variables **s**_j_ were categorical variables (such as sex, smoking status, and genotype). Notably, the categorical label **s**_k_ only applies to the input variable **s**_j_. A detailed description of the horizontal IF process for input labels and continuous input, as well as the vertical IF process and output preprocessing, are provided in the supplementary materials, Supplemental Digital Content 2, http://links.lww.com/JS9/C689.

The input categorical variables are unified as a vector **s**_j_=[s_1_, s_2_ …, s_n_] (Table S1, Supplemental Digital Content 2, http://links.lww.com/JS9/C689). The output continuous variables (v_ij_) are labeled “Within” when the observed INR is within the range of 1.8-2.5; otherwise, the output variable is labeled “Out.” Furthermore, N_vars_=235 input continuous variables (v_ki_) and N_labels_=62 input labeling variables (**s**_j_) are included. After the initial IF process, we performed two additional IF and data rearrangement processes. The first was an IF horizontal process, and the second was an IF vertical process (more details are shown in the supplementary materials, Supplemental Digital Content 2, http://links.lww.com/JS9/C689). The IF horizontal process was applied to input labels **s**_j>0_ and continuous input variables v_ki_.

#### IFPTML linear vs. nonlinear algorithms

LDA and MLR were proposed as the linear classification and regression model algorithms. In these linear models, we used the objective function value (f(v_ij_)_obj_, supervised learning) as the algorithm’s input, as well as the other input variables. The input variables were the function of reference f(v_ij_)_ref_, the original variables v_ki_, and their first-order PTOs such as ∆v_k_(**s**_j_) or second-order PTOs such as Euclidean distance ǁ∆v_k_(**s**_j_)ǁ_cj_. After defining the input variables, the LDA algorithm was used to fit the model by determining the coefficients a_1_, a_k,s_, and a_c,s_ values. Similarly, we also used these input variables to train and validate nonlinear IFPTML models via different algorithms. The specificity (Sp) (%), sensibility (Sn) (%), χ^2^, and *P* value were used to measure the performance of the classification model. Furthermore, the correlation coefficient, mean absolute error (MAE), root mean square error (RMSE), relative absolute error (RAE), and root relative square error (RRSE) were used for regression analysis to measure the performance of the regression models. In addition, we utilized different classification models, such as general discriminant analysis (GDA), BayesNet (BN), logistic, multilayer perceptron (MLP), support vector machine (SVM), K-nearest neighbor (KNN), and random forest (RF) models. We also used multiple regression models, such as linear regression (LR), MLP, SVM, KNN, bagging, and RF models, to train/validate our model. The methods used for calculating the output variable and posterior probability are described in the supplementary materials, Supplemental Digital Content 2, http://links.lww.com/JS9/C689. The GDA model was performed using STATISTICA (v 6.0) software. Other AI/ML algorithms utilized WEKA (v 3.8.6) software.

## Results

### Clinical results

A total of 246 patients with 3261 INRs were enrolled in this study. Fifty-four single-nucleotide polymorphisms (SNPs) in related genes were detected in these patients. The median duration of follow-up was 99 days (range: 12–188 days). Some of the clinical characteristics of the enrolled patients are shown in Table 1; more detailed information is shown in Tables S1, Supplemental Digital Content 2, http://links.lww.com/JS9/C689 and S2, Supplemental Digital Content 2, http://links.lww.com/JS9/C689. The SNP frequency of the patients’ additional genes is shown in Table S3, Supplemental Digital Content 2, http://links.lww.com/JS9/C689.

**Table 1:** Sets of categorical variables (s_k_) and continuous (v_ki_) input variables, characteristics of the patients in the study, and their partitions (s_j_ and c_k_).

Sets	Subset	v_ki_ **/s**_k_	Variable	Value
**s**_j_	**s**_I_=DemoCat	s_1_	Male	157 (63.8%)
	**s**_I_=DemoCat	s_1_	Female	89 (36.2%)
	**s**_III_=PKGeneCat	s_9_	*CYP2C9* (rs1057910) *1/*1	227 (92.3%)
	**s**_III_=PKGeneCat	s_9_	*CYP2C9* (rs1057910) *1/*3	19 (7.7%)
	**s**_IV_=PDGeneCat	s_24_	*VKORC1* (rs9923231) AA	209 (85.5%)
	**s**_IV_=PDGeneCat	s_24_	*VKORC1* (rs9923231) AG	34 (13.8%)
	**s**_IV_=PDGeneCat	s_24_	*VKORC1* (rs9923231) GG	3 (1.2%)
	**s**_II_=TypeCat	s_7_	DVR	25 (10.2%)
	**s**_II_=TypeCat	s_7_	AVR	36 (14.6%)
	**s**_II_=TypeCat	s_7_	MVR	97 (39.4%)
	**s**_II_=TypeCat	s_7_	TVR	3 (1.2%)
	**s**_II_=TypeCat	s_8_	Mechanical valve	86 (35.0%)
	**s**_II_=TypeCat	s_8_	Bioprosthetic valve	123 (50.0%)
**c**_k_	**c**_III_=Demography	v_127_	Age (years)	58 ± 13
	**c**_III_=Demography	v_128_	Height (cm)	164.7 ± 8.8
	**c**_III_=Demography	v_129_	Weight (kg)	63.9 ±11.8
	**c**_I_=Warfarin daily dosage regime	v_1_-v_63_	Doses (mg/d)	2.12±0.96
	**c**_II_=Days of warfarin administration prior to INR measurement	v_64_-v_126_	Treatment times (day)	6.7±7.8

AVR, aortic valve replacement; DVR, aortic and mitral valve replacement; INR, international normalized ratio; MVR, mitral valve replacement; TVR, tricuspid valve replacement.

Blood counts and blood biochemistry indices were recorded during the follow-up period. The hospital clinical laboratory measured blood cell counts and biochemistry as needed. Generally, blood and biochemical data were collected more frequently during hospitalization than before, and blood analysis was less frequent in the outpatient department. In most cases, only the INR was monitored when patients were followed up in outpatient clinics. The blood count and biochemical indicator data are shown in Tables S4, Supplemental Digital Content 2, http://links.lww.com/JS9/C689 and S5, Supplemental Digital Content 2, http://links.lww.com/JS9/C689, respectively.

In clinical practice, patients usually have multiple diseases, and other drugs are needed to treat comorbidities. This study focused mainly on warfarin; only the daily dosage of the combined drugs was recorded. The characteristics of the patients taking combined medication are shown in Table S6, Supplemental Digital Content 2, http://links.lww.com/JS9/C689.

### Computational results

#### Univariate analysis

Using univariate analysis, all the continuous and categorical input variables were first assessed to predict warfarin blood levels. The results of each variable for continuous output variables are shown in Table S7, Supplemental Digital Content 2, http://links.lww.com/JS9/C689 (continuous input variables) and S8, Supplemental Digital Content 2, http://links.lww.com/JS9/C689 (categorical input variables). The results of each variable for the categorical output variables are shown in Table S9, Supplemental Digital Content 2, http://links.lww.com/JS9/C689 (continuous input variables) and S10, Supplemental Digital Content 2, http://links.lww.com/JS9/C689 (categorical input variables). The results of univariate analysis were not the only strategy for model establishment. For instance, the variables CYP2C9 (rs1057910) and CYP4F2(rs2108622) had no statistical significance after univariate analysis for the categorical output variables. However, we also introduced these two variables during the subsequent analysis because many studies have demonstrated that these two variables have physiological significance for warfarin.

#### Regression model

**Figure 3:**
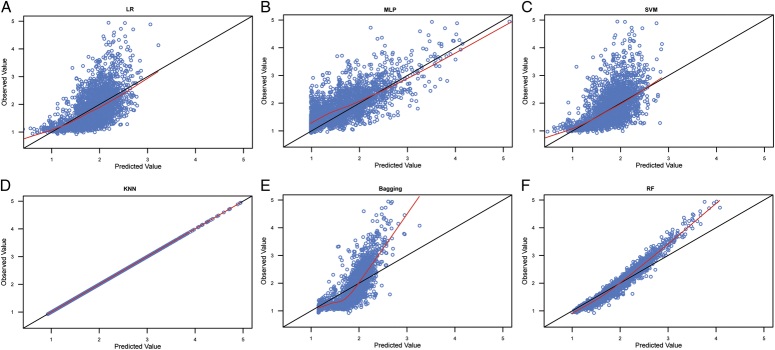
Scatter plot of the observed values and model-predicted values in the training datasets with the raw data. A: scatter plot of the observed values and model-predicted values for LR algorithm; B: scatter plot of the observed values and model-predicted values for MLP algorithm; C: scatter plot of the observed values and model-predicted values for SVM algorithm; D: scatter plot of the observed values and model-predicted values for KNN algorithm; E: scatter plot of the observed values and model-predicted values for Bagging algorithm; F: scatter plot of the observed values and model-predicted values for RF algorithm. LR, linear regression; MLP, multilayer perceptron; SVM, support vector machine; KNN, K-nearest neighbor; RF, random forest.

All regression models were generated using WEKA software. The selected classifiers for the different algorithms in WEKA software are shown in Table S11, Supplemental Digital Content 2, http://links.lww.com/JS9/C689. The input variables for the first order of the IFPTML model, called MA ∆v_k_(s_j_), and the second order of the IFPTML model, called Euclidean distances ǁ∆v_k_(s_j_)ǁ, are shown in Tables S12, Supplemental Digital Content 2, http://links.lww.com/JS9/C689 and S13, Supplemental Digital Content 2, http://links.lww.com/JS9/C689. We used raw warfarin dosage data as input variables in the IFPTML model. Table 2 shows the parameters that can quantify the performance of the IFPTML regression model. The goodness-of-fit results for the six training and validation raw data algorithms are shown in the scatter plots presented in Figures 3 and S1, Supplemental Digital Content 2, http://links.lww.com/JS9/C689, respectively. The predicted biases (PBs) for the six algorithms for the raw data in the training and validation sets are shown in Table 3 and Figure S2, Supplemental Digital Content 2, http://links.lww.com/JS9/C689 and S3, Supplemental Digital Content 2, http://links.lww.com/JS9/C689. The optimal algorithm was determined based on a significant correlation coefficient and the minimum parameters of MAE, RMSE, RAE, and RRSE. According to these criteria, the results showed that the RF algorithm was the optimal algorithm (Table 2), and the proportion of ideal predictions of the validation data was 59.0% (Table 3) for fitting with the raw data. However, the prediction performance did not significantly improve when the MA ∆v_ki_(**s**_j_) and ED data were processed (Table 2). Although the correlation coefficient of the RF algorithm in the validation set of MA ∆v_ki_(**s**_j_) data was slightly greater than that of the raw data (62.9% *vs*. 59.5%), the MAE, RMSE, and RAE of the RF algorithm in the validation set of the raw data were slightly smaller than those of the MA ∆v_ki_(**s**_j_) data.

**Table 2:** Regression parameters for different data levels using WEKA.

		Corr. Coeffi.			MAE			RMSE			RAE (%)			RRSE (%)		
AI/ML	Set	A	B	C	A	B	C	A	B	C	A	B	C	A	B	C
LR	t	0.588	0.605	0.562	0.377	0.591	0.62	0.507	0.796	0.826	78.6	77.3	81.1	80.9	79.6	82.7
	v	0.527	0.448	0.016	0.397	0.645	0.843	0.537	0.933	3.59	82.6	84.2	110.1	85.3	93	358
MLP	t	0.751	0.652	0.294	0.359	0.611	0.779	0.482	0.838	1.066	74.9	79.8	101.8	77	83.9	106.7
	v	0.475	0.431	−0.05	0.483	0.701	1.621	0.687	1.014	20.823	100.5	91.5	211.7	109.2	101.2	2076.8
SVM	t	0.564	0.583	0.516	0.353	0.573	0.596	0.525	0.824	0.866	73.6	74.9	78	83.7	82.5	86.7
	v	0.524	0.43	0.143	0.395	0.628	0.701	0.543	0.932	1.477	82.2	82	91.5	86.3	92.9	147.3
KNN	t	1	0.991	0.9997	0	0.035	0.002	0	0.134	0.025	0	4.5	0.2	0	13.4	2.5
	v	0.305	0.397	0.363	0.482	0.726	0.761	0.706	1.057	1.137	100.3	94.8	99.4	112.3	105.4	113.4
Bagging	t	0.753	0.774	0.765	0.317	0.498	0.507	0.443	0.68	0.702	66.1	65	66.3	70.7	68	70.3
	v	0.562	0.573	0.514	0.371	0.608	0.623	0.524	0.828	0.864	77.2	79.4	81.4	83.3	82.6	86.1
RF	t	0.978	0.969	0.973	0.135	0.231	0.222	0.19	0.32	0.313	28.1	30.1	29	30.4	32	31.4
	v	0.595	0.629	0.553	0.362	0.578	0.601	0.51	0.787	0.837	75.3	75.4	78.5	81.1	78.5	83.5

A, raw data; AI/ML, artificial intelligence/machine learning; B, MA ∆v_ki_(**s**_j_) data; C, ED ǁ∆v_k_(**s**_j_)ǁ data; Corr. Coeffi., correlation coefficient; KNN, K-nearest neighbor; LR, linear regression; MAE, mean absolute error; MLP, multilayer perceptron; RAE, relative absolute error; RF, random forest; RMSE, root mean squared error; RRSE, root relative squared error; SVM, support vector machine; t, training; v, validation.

**Table 3:** Predicted bias results of the regression for different data levels in different predicted groups using WEKA software.

		PB<−20%			−20%≤PB≤20%			PB>20%		
AI/ML	Set	A	B	C	A	B	C	A	B	C
LR	t	15.1	15.2	15.5	57.5	57.8	56.0	27.4	27.0	28.5
	v	15.6	14.3	17.3	54.1	56.4	51.4	30.3	29.4	31.3
MLP	t	31.6	30.3	37.8	60.1	59.3	43.2	8.3	10.4	19.0
	v	35.3	30.7	38.5	48.4	56.4	41.5	16.3	12.9	20.0
SVM	t	17.8	18.5	18.8	62.8	60.9	59.6	19.4	20.6	21.6
	v	19.2	18.8	21.0	57.9	56.9	56.8	23.0	24.3	22.2
KNN	t	0.0	0.3	0.0	100.0	98.8	100.0	0.0	0.9	0.0
	v	17.2	22.0	19.9	55.0	54.8	56.4	27.8	23.2	23.7
Bagging	t	10.8	10.8	10.8	66.6	64.8	65.4	22.7	24.3	23.8
	v	13.8	13.6	13.9	60.0	56.8	56.4	26.3	29.6	29.7
RF	t	0.5	0.7	0.4	96.8	95.1	95.2	2.7	4.1	4.4
	v	11.8	13.8	12.0	59.0	57.6	58.4	29.2	28.6	29.6

A, raw data; AI/ML, artificial intelligence/machine learning; B, MA ∆v_ki_(**s**_j_) data; C, ED ǁ∆v_k_(**s**_j_)ǁ data; KNN, K-nearest neighbor; LR, linear regression; MLP, multilayer perceptron; PB, predicted bias; RF, random forest; SVM, support vector machine; t, training; v, validation.

**Figure 4:**
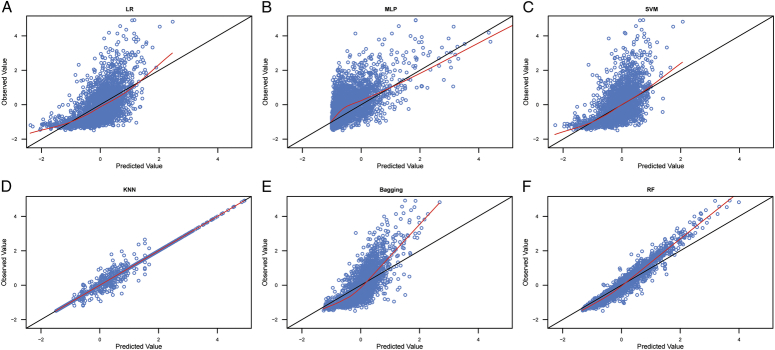
Scatter plot of the observed values and model-predicted values in the training datasets with moving average ∆vki(sj) data. A: scatter plot of the observed values and model-predicted values for LR algorithm; B: scatter plot of the observed values and model-predicted values for MLP algorithm; C: scatter plot of the observed values and model-predicted values for SVM algorithm; D: scatter plot of the observed values and model-predicted values for KNN algorithm; E: scatter plot of the observed values and model-predicted values for Bagging algorithm; F: scatter plot of the observed values and model-predicted values for RF algorithm. LR, linear regression; MLP, multilayer perceptron; SVM, support vector machine; KNN, K-nearest neighbor; RF, random forest.

The estimated parameters for the MA ∆v_ki_(**s**_j_) data are shown in Table 2. The goodness-of-fit results for the six algorithms for the training and validation MA ∆v_ki_(**s**_j_) data are shown in the scatter plots in Figure 4 and S4, Supplemental Digital Content 2, http://links.lww.com/JS9/C689, respectively. The PBs for the six MA ∆v_ki_(s_j_) data algorithms in the training and validation sets are shown in Table 3 and Figures S5, Supplemental Digital Content 2, http://links.lww.com/JS9/C689 and S6, Supplemental Digital Content 2, http://links.lww.com/JS9/C689. All these results confirm that the RF algorithm performed the best in fitting the MA ∆v_ki_(**s**_j_) data.

**Figure 5:**
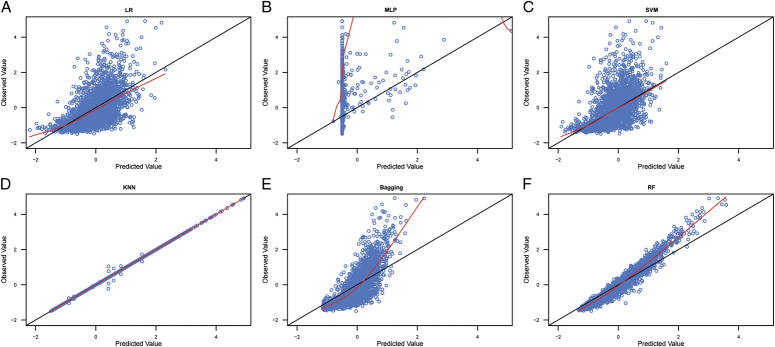
Scatter plot of the observed values and model-predicted values in the training datasets with Euclidean distance ǁ∆vk(sj)ǁ data. A: scatter plot of the observed values and model-predicted values for LR algorithm; B: scatter plot of the observed values and model-predicted values for MLP algorithm; C: scatter plot of the observed values and model-predicted values for SVM algorithm; D: scatter plot of the observed values and model-predicted values for KNN algorithm; E: scatter plot of the observed values and model-predicted values for Bagging algorithm; F: scatter plot of the observed values and model-predicted values for RF algorithm. LR: linear regression; MLP: multilayer perceptron; SVM: support vector machine; KNN: K-nearest neighbor; RF: random forest.

The estimated parameters for the ǁ∆v_k_(**s**_j_)ǁ data are shown in Table 2. The goodness-of-fit results for the six algorithms used for training and validation ǁ∆v_k_(**s**_j_)ǁ data are shown in the scatter plots in Figures 5 and S7, Supplemental Digital Content 2, http://links.lww.com/JS9/C689, respectively. The PBs for the six algorithms on the ǁ∆v_k_(**s**_j_)ǁ dataset in the training and validation sets are shown in Table 3 and Figures S8, Supplemental Digital Content 2, http://links.lww.com/JS9/C689 and S9, Supplemental Digital Content 2, http://links.lww.com/JS9/C689, respectively. All these results confirm that the RF algorithm had the best performance for fitting the ED ǁ∆v_k_(**s**_j_)ǁ data.

#### Classification model

STATISTICA software was applied to train/validate the IFPTML-LDA classification linear model. The classification was performed for the output as the categorical variable, including outputs within and out of the target range according to the raw INR value. Only the continuous variables were included as the input attributes. However, the categorical variables used as the input attributes were excluded from the raw data during the GDA. We defined the function of reference and the PTOs of zero, first, or second order according to the case/order of the model as the input. Initially, we scanned all the hyperparameters α, q, and r. For the zero-order model (α=0, q=1, and r=1), we used the raw original variables v_ki_. For the first-order model (α=1, q=1, and r=1), we used the PTOs of first-order ∆v_ki_(**s**_j_), and for the second-order model (α=1, q=2, and r=½), we used the second-order PTOs ǁ∆v_k_(**s**_j_)ǁ.

In the first IFPTML-LDA model, we used the original/raw data as the input variable. Additionally, for variable selection, we applied forward stepwise (FSW) selection complemented by an expert-guided selection (EGS) strategy to select the most essential features. The formula of the best-identified model is described as follows (Equation 2):


$$f{\left(v_{\mathrm{ij}}\right)}_{\mathrm{calc}}=-1.6772+0.1204\times \mathrm{Dose}3+0.0112\;\times \mathrm{POD}+0.1188\times \mathrm{Cefoperazone}\;+0.9305\times \mathrm{Ceftriaxone}+0.1086\;\times \mathrm{Ticarcillin}+12.84\;\times \mathrm{Tigecycline}\;+9.82\times \mathrm{Spironolactone}\;-0.0644\;\times \mathrm{Neutrophil}+2.264\times \mathrm{Plateletcrit}\;-0.019\times \mathrm{Human\_albumin}\;(\mathrm{Eq}.\;2)\;$$

$${N_{\mathrm{train}}=2446\;\chi }^{2}=222.839\;P<0.05$$

where Dose 3 represents the day 3 warfarin dose before the INR, and POD represents postoperative days.

The total prediction accuracies of GDA for the raw data were 61.2% and 64.0% for the training and validation datasets, respectively. The results of the other algorithms for the raw data using WEKA software are shown in Table 4. The RF algorithm had the highest number of correctly classified instances, with 68.6% in the validation dataset.

**Table 4:** Classification analysis results of the prediction performance for different data levels using WEKA.

			TPR			FPR			Precision			Recall			F-Measure			ROC		
AI/ML	Set	Class	A	B	C	A	B	C	A	B	C	A	B	C	A	B	C	A	B	C
BN	t	Out	0.718	0.766	0.908	0.483	0.198	0.091	0.735	0.878	0.949	0.718	0.766	0.908	0.726	0.818	0.928	0.694	0.832	0.954
		Within	0.517	0.802	0.909	0.282	0.234	0.092	0.497	0.648	0.841	0.517	0.802	0.909	0.507	0.717	0.874	0.694	0.832	0.954
	v	Out	0.743	0.779	0.923	0.519	0.196	0.126	0.727	0.881	0.931	0.743	0.779	0.923	0.735	0.827	0.927	0.688	0.831	0.937
		Within	0.481	0.804	0.874	0.257	0.221	0.077	0.502	0.662	0.859	0.481	0.804	0.874	0.491	0.726	0.866	0.688	0.831	0.937
Logistic	t	Out	0.843	0.981	0.984	0.543	0.036	0.023	0.743	0.981	0.987	0.843	0.981	0.984	0.79	0.981	0.986	0.773	0.994	0.996
		Within	0.457	0.964	0.977	0.157	0.019	0.016	0.611	0.964	0.971	0.457	0.964	0.977	0.523	0.964	0.974	0.773	0.994	0.996
	v	Out	0.794	0.966	0.962	0.586	0.06	0.084	0.716	0.968	0.955	0.794	0.966	0.962	0.753	0.967	0.959	0.686	0.981	0.98
		Within	0.414	0.94	0.916	0.206	0.034	0.038	0.52	0.937	0.929	0.414	0.94	0.916	0.461	0.939	0.922	0.686	0.981	0.98
MLP	t	Out	0.75	0.986	0.999	0.461	0.096	0.974	0.752	0.95	0.656	0.75	0.986	0.999	0.751	0.968	0.792	0.675	0.981	0.231
		Within	0.539	0.904	0.026	0.25	0.014	0.001	0.537	0.971	0.917	0.539	0.904	0.026	0.538	0.936	0.05	0.675	0.981	0.231
	v	Out	0.726	0.981	0.985	0.537	0.074	0.968	0.716	0.961	0.654	0.726	0.981	0.985	0.721	0.971	0.786	0.627	0.982	0.249
		Within	0.463	0.926	0.032	0.274	0.019	0.015	0.477	0.964	0.529	0.463	0.926	0.032	0.47	0.945	0.06	0.627	0.982	0.249
SVM	t	Out	0.882	0.975	0.976	0.599	0.076	0.056	0.733	0.96	0.97	0.882	0.975	0.976	0.8	0.968	0.973	0.642	0.95	0.96
		Within	0.401	0.924	0.944	0.118	0.025	0.024	0.646	0.953	0.955	0.401	0.924	0.944	0.495	0.938	0.949	0.642	0.95	0.96
	v	Out	0.84	0.968	0.97	0.621	0.053	0.06	0.715	0.972	0.968	0.84	0.968	0.97	0.773	0.97	0.969	0.609	0.958	0.955
		Within	0.379	0.947	0.94	0.16	0.032	0.03	0.56	0.941	0.944	0.379	0.947	0.94	0.452	0.944	0.942	0.609	0.958	0.955
KNN	t	Out	1	0.999	1	0	0.002	0	1	0.999	1	1	0.999	1	1	0.999	1	1	1	1
		Within	1	0.998	1	0	0.001	0	1	0.999	1	1	0.998	1	1	0.998	1	1	1	1
	v	Out	0.647	0.826	0.828	0.477	0.407	0.382	0.716	0.791	0.801	0.647	0.826	0.828	0.68	0.808	0.814	0.585	0.707	0.723
		Within	0.523	0.593	0.618	0.353	0.174	0.172	0.443	0.648	0.659	0.523	0.593	0.618	0.48	0.619	0.638	0.585	0.707	0.723
RF	t	Out	1	0.998	1	0	0	0	1	1	1	1	0.998	1	1	0.999	1	1	1	1
		Within	1	1	1	0	0.002	0	1	0.997	1	1	1	1	1	0.998	1	1	1	1
	v	Out	0.84	0.962	0.943	0.6	0.263	0.302	0.722	0.872	0.853	0.84	0.962	0.943	0.777	0.915	0.896	0.698	0.965	0.936
		Within	0.4	0.737	0.698	0.16	0.038	0.057	0.573	0.913	0.869	0.4	0.737	0.698	0.471	0.816	0.774	0.698	0.965	0.936

A, raw data; AI/ML, artificial intelligence/machine learning; B, MA ∆v_ki_(**s**_j_) data; BN, BayesNet; C, ED ǁ∆v_k_(**s**_j_)ǁ data; FPR, false positive rate; KNN, K-nearest neighbor; MLP, multilayer perceptron; RF, random forest; ROC, receiver operating characteristic; SVM, support vector machine; t, training; TPR, true positive rate; v, validation.

The total prediction accuracies of GDA for the MA ∆v_ki_(**s**_j_) dataset were 95.4% and 95.6% for the training and validation datasets, respectively. The second IFPTML-LDA algorithm used the first order of PTO (∆v_ki_(s_j_)) as input variables to train/validate the model. The best IFPTML model is described below (Equation 3). The results of other algorithms for MA ∆v_ki_(**s**_j_) data using WEKA software are shown in Table 4.


$$\;f{\left(v_{\mathrm{ij}}\right)}_{\mathrm{calc}}=17.04-34.04\times f{\left(v_{\mathrm{ij}}\right)}_{\mathrm{ref}}+0.6244\;\times \Delta \mathrm{Dose}01\left(\mathrm{TypeCat}\right)-1.137\times \Delta \mathrm{CEFUS}\left(\mathrm{ROVTypeCat}\right)\;-0.5472\times \Delta \mathrm{Dose}01\left(\mathrm{PKGeneCat}\right)-6.191\;\times \Delta \mathrm{Bifidobacterium}\left(\mathrm{PKGeneCat}\right)\;-0.162\times \Delta \mathrm{HALB}\left(\mathrm{VKPKGeneCat}\right)\;+0.8073\times \Delta \mathrm{CEFUS}\left(\mathrm{ROVNRGeneCat}\right)-1.585\;\times \Delta \mathrm{HDCL}\left(\mathrm{NRGeneCat}\right)+0.3398\;\times \Delta \mathrm{Dose}03\left(\mathrm{ClotGeneCat}\right)-0.2648\;\times \Delta \mathrm{PDW}\left(\mathrm{ClotGeneCat}\right)\;(\mathrm{Eq}.3)$$

$${N_{\mathrm{train}}=2446\;\chi }^{2}=2919.95\;P<0.05$$

where Dose01 represents the day 1 warfarin dose prior to the INR; CEFUS represents cefuroxime sodium; HALB represents human albumin; HDCL represents high-density cholesterol; Dose03 represents the day 3 warfarin dose prior to the INR; and PDW represents the platelet distribution width.

The third IFPTML-LDA model is used as the input variable, and the second is PTO ǁ∆v_k_(**s**_j_)ǁ. The total predicted accuracy of GDA for ǁ∆v_k_(**s**_j_)ǁ data was 94.6% and 95.7% for the training and validation datasets, respectively. The best IFPTML-GDA model is described below (Equation 4). The results of other algorithms for ǁ∆v_k_(**s**_j_)ǁ data using WEKA software are shown in Table 4. Of all the algorithms, the IFPTML-LDA algorithm using the first-order PTO (∆v_ki_(**s**_j_)) data had the highest number of correctly classified instances, with 96.0% in the validation dataset for the ǁ∆v_k_(**s**_j_)ǁ data.


(Eq. 4) $$f{\left(v_{\mathrm{ij}}\right)}_{\mathrm{calc}}=17.81-34.83\times f{\left(v_{\mathrm{ij}}\right)}_{\mathrm{ref}}\;+0.115\times ǁ∆V_{k}\left(\mathrm{DoseTypeCat}\right)ǁ-0.1031\;\times ǁ∆V_{k}\left(\mathrm{MWDiureticTypeCat}\right)ǁ-0.1314\;\times ǁ∆V_{k}\left(\mathrm{MWDiureticPKGeneCat}\right)ǁ+10.93\;\times ǁ∆V_{k}\left(\mathrm{LOGDAspirinPKGeneCat}\right)ǁ\;+40.92\times ǁ∆V_{k}\left(\mathrm{LOGDDiclofenacPKGeneCat}\right)ǁ\;-0.0104\times ǁ∆V_{k}(\mathrm{Blood\_cell\_countPKGeneCat})ǁ\;-0.069\times ǁ∆V_{k}(\mathrm{DEMOPDGeneCat})ǁ\;+0.0302\times ǁ∆V_{k}(\mathrm{DEMOVKPKGeneCat})ǁ\;-0.6514\times ǁ∆V_{k}\left(\mathrm{ProbioticsVKPKGeneCat}\right)ǁ\;$$

$${N_{\mathrm{train}}=2446\;\chi }^{2}=2952.951\;P<0.05$$

### Program for warfarin blood level prediction

Overall, discriminant analysis was the algorithm with the best performance to predict the categorical output using the MA ∆v_ki_(s_j_) data for classification analysis (Equation 3). A program was designed to predict warfarin blood levels. This program used a file with all the test results to perform the prediction calculations. Once the necessary changes were made after running the program, a new file with the predictions was automatically created in the same directory from which the information from the input file was obtained. The type of mutation in each gene and the status of each categorical variable (such as sex or smoking status) in Excel were converted into a string of characters readable by the script. The specific mutations for each selected gene and categorical variables within each group were selected via drop-down lists. All the necessary data from Equation 3 were entered into a template Excel file to make the prediction. This program is stored in the GitHub repository (https://github.com/hbediaga/warfarin.PTML).

## Discussion

This study aimed to construct a precise model for predicting warfarin blood level based on clinical longitudinal data using the IFPTML model. The IFPTML model has been widely used to identify the activity of new chemical compounds in drug discovery^24^. Although numerous warfarin prediction algorithms have been developed using MLR or PMX, few studies have reported using the IFPTML model in clinical data analysis. Many factors are related to warfarin dosage requirements, including combined chemical drugs, herbal medicines, dietary supplements^35^, and genetic polymorphisms^34^. Previous studies on the precision of warfarin dosages have focused mainly on predicting the optimal initial or stable dose via ML models^36^. However, the results of these reports may not be helpful in the long-term management of warfarin anticoagulant therapy because clinicians need more guidance on how to adjust the warfarin dosage during the duration of anticoagulant therapy. We conducted a study to explore the effects of numerous factors on warfarin response using the IFPTML algorithm based on longitudinal follow-up data. The results showed that RF was the best algorithm for predicting the continuous output from the raw data (Table 2). The IFPTML-GDA model was the best algorithm for predicting categorical outputs from the MA ∆v_ki_(**s**_j_) data for classification analysis.

The present study simultaneously conducted regression and classification analyses for clinical longitudinal data. Because several scenarios exist in clinical practice, such as adjusting the warfarin dosage according to the target INR when clinicians know the patient’s INR, the regression model could be helpful. When clinicians are concerned about whether the INR could be archived within the target range at a specific dose, the results of the classification model might be a helpful tool. For instance, a clinical physician wants to know the initial warfarin dose for a new patient and measures the INR after 3 days of warfarin administration. The user could input the variables included in the model and set the target INR range as 1.8–2.5, then test different dosages to determine which dose could achieve the target INR range using the regression model and the derived dosage as the initial dose. Then, the user could input the model variables and the daily dose of warfarin from the patient’s last INR measurement to this INR measurement period and test different dosages to determine which dose could achieve the target INR range using the regression model. The model could derive the subsequent dose of warfarin. Generally, patients seen in the outpatient department are those who have been taking warfarin for a long time. Clinicians may be concerned about whether the target INR could be achieved at a specific dose for outpatient patients, for which the classification model might be a helpful tool. If the input variables of the model for patients change, the user inputs the changed variables into the model to derive the suitable dosage for the subsequent dose. The current study provides a flexible tool for determining the exact warfarin dosage.

ML aims to describe the mathematical function between observed output variables and the corresponding input variables using additional free parameters and complex interactions. This complexity makes the model difficult to interpret physiologically; this model is called the “black box.” Most ML algorithms are designed to handle high-dimensional data^37^. Generally, it is necessary to perform data preprocessing before analysis using ML algorithms because the raw data are usually unable to be used directly and often have complex nonlinear relationships^38^. Therefore, the MA ∆v_ki_(**s**_j_) and ǁ∆v_k_(**s**_j_)ǁ distances were calculated for different feature groups (categorical variables). The standard algorithms utilized in the present study included SVM, KNN, RF, and MLP. The ML algorithm was trained using a data-driven approach that does not consider biological/pharmacological mechanisms^37^. Therefore, discussing the impact of factors on model parameters, such as the PMX analysis, is unnecessary. However, this did not mean the data could be collected at will during the research process, and irrelevant data might decrease the model fitting performance. Researchers should conduct a detailed literature review and clarify which factors impact the output variables before conducting project research. During execution, the factors that impact the output variables should be collected as often as possible. To determine the critical factors (input attributes), we performed a univariate analysis of automatically selected attributes using WEKA software and expert experience before performing the data analysis via ML algorithms.

The data were split into a training set and a validation set. The training set was used to fit the model to different algorithms, and the validation set was used to evaluate the model in the different algorithms^38^. The optimal algorithm was determined based on a significant correlation coefficient and the MAE, RMSE, RAE, and RRSE minimum parameters. According to these criteria, the RF model was the optimal algorithm, with a correlation coefficient of 0.595 and an MAE of 0.362(Table 2); the proportion of ideal predictions was 59.0% for fitting with the raw data in the validation set (Table 3). However, the prediction performance did not significantly improve when the MA ∆v_ki_(**s**_j_) and ED ǁ∆v_k_(**s**_j_)ǁ data were used (Table 2). Although the correlation coefficient of the RF algorithm in the validation set of MA ∆v_ki_(**s**_j_) data was slightly more significant than that of the raw data (62.9% vs. 59.5%), the MAE, RMSE, and RAE of the RF algorithm in the validation set of the raw data were slightly smaller than those of the MA ∆v_ki_(**s**_j_) data. Compared with published studies that constructed models with ML algorithms, the model’s predictive performance in this study had some inconsistencies. The correlation coefficient of this model in the validation set was lower than those of the ANN model of Saleh *et al.* ^39^. (0.656), TWIST system (0.693)^40^, the EEM model of Tao *et al.* ^20^. (0.664), the kernel-based SVR model of Maghsoudi *et al.* ^41^. (0.825), and the LightGBM algorithm model of Liu *et al.* ^42^. (0.872) but higher than that of the ANFIS model of Tao *et al.* ^18^. (0.313). The percentage of ideal predictions in this study was close to those of the ANFIS model of Tao *et al.* ^18^. (63.7% for internal validation and 60.6% for external validation), the hybrid model with a genetic algorithm and back propagation neural network of Li *et al.* ^19^. (58.7% for internal validation and 62.9% for external validation), and the EEM model of Tao *et al.* ^20^. (52.7%). However, the percentage of ideal predictions in this study was more significant than those of the models reported by Saleh *et al.* ^39^. (47.8%), Truda *et al.* ^14^. (46.6%), Asiimwe *et al.* ^43^. (50%), and Jahmunah *et al.* ^44^. (38.6%) and lower than those of the LightGBM algorithm model of Liu *et al.* ^42^. (68%), the ANFIS model of Gu *et al.* ^15^. (73.5%), the GBM model of Nguyen *et al.* ^45^. (73.8%) and the SSNN model of Ma *et al.* ^46^. (75.6%). Most reported ML algorithms for predicting warfarin dosage are based on cross-sectional data. The algorithm in the current study was used to predict blood levels after warfarin administration. It may be why the current study’s results are inconsistent with those of previous reports. It was not necessary to compare the difference in the MAE between the current study and previous reports because the current study used it to evaluate the predictive blood levels. In contrast, previous studies used it to evaluate the predictive dosage.

Huang *et al.* ^47^. and Lee *et al.* ^48^. constructed INR prediction models using ML algorithms. The correlation coefficients and proportion of ideal INR predictions for the validation set were more significant in the present study than in the study by Huang *et al.* ^47^. (with a 0.473 correlation coefficient and a 47.1% INR ideal prediction). The current study had a similar MAE for the INR (0.362) for the validation set as that of Huang *et al.* ^47^. (0.376). Lee *et al.* ^48^. did not report the proportion of the predicted INR within 20% of the observed INR; however, they reported the proportion of the predicted INR within distances of 0.2, 0.25, and 0.3, or distances of greater than 0.5 and greater than 1.0 from the observed INR. We chose to compare the proportion of predicted INR values within 0.3 of the observed INR because the INR MAE was 0.362. Therefore, in the current study, the percentage of ideal predictions was similar to that of the Lee *et al.* ^48^. model (53.4%).

We found that several algorithms had similar prediction performances during the classification analysis. GDA merely analyzes linear variables. Therefore, categorical variables were excluded from the classification analysis using raw data. It led to the loss of a substantial amount of information, such as information about the effect of genes. Hence, the prediction performance of the classification analysis was relatively poor when using the raw data (Table 4). The MA of continuous variables was calculated for different attribute groups to consider the effect of categorical variables. The distance between each continuous variable and the MA and Euclidean distances ǁ∆v_k_(**s**_j_)ǁ of similar attributes of continuous variables were further calculated. The prediction performance was significantly improved for classification analysis using MA ∆v_ki_(**s**_j_) and Euclidean distance ǁ∆v_k_(**s**_j_)ǁ data (Table 4). The TPRs of the logistic, MLP, and SVM algorithms exceeded 90% for classification analysis in different groups using MA ∆v_ki_(**s**_j_) data, and the TPRs of the logistic and SVM algorithms were also more significant than 90% using Euclidean distance ǁ∆v_k_(**s**_j_)ǁ data. These algorithms were generated using WEKA software, and it could not be determined which variables affected the warfarin response (black box). All the input variables could be considered to have effects on warfarin efficacy.

GDA was selected as the classification algorithm using STATISTICA software. The GDA algorithm provides an explicit equation that describes the relationship between the output and input variables. The total TPRs of the GDA algorithm were 95.4% and 95.6% for the training and validation sets, respectively, with MA ∆v_ki_(**s**_j_) data, and 94.6% and 95.7% for the training and validation sets, with Euclidean distance ǁ∆v_k_(**s**_j_)ǁ data. Therefore, MA ∆v_ki_(**s**_j_)-level data might be sufficient for future GDA algorithm-based clinical applications. The variables included in the model using MA ∆v_ki_(**s**_j_) data for classification analysis are shown in Equation 3. Therefore, it was easy to understand the relationship between the output and input variables using the GDA algorithm for classification analysis. No studies have considered the output (INR or warfarin dosage) of the algorithm as a categorical variable, so we did not compare the results of the classification analysis for the current study with those of other studies.

Overall, compared with other reported AI/ML algorithms for warfarin, the current research features are as follows: patient data were longitudinal data, and the INR was selected as the output variable. Most of the reported ML algorithms for determining warfarin dosage use cross-sectional data. The data were downloaded from the IWPC (www.pharmgkb.org/downloads)^12,14,39,44^ or CLIATHVR (unpublished)^18,19,46,49,50^ datasets and were used to predict the maintenance dose^12,16,18–20,39,40,42–44,46,48–51^. The dataset used in the present study was more extensive than that used in previously reported ML algorithms. It might be because there are more interpretations of warfarin variability. The limitations of the current study include the following: the sample size was relatively small, unrelated (or nonphysiological) variables were included in the IFPTML model, external data did not evaluate the model, and a web server based on the foundation of the current study to facilitate access for clinical applications was not developed. An evaluation of the model and a more user-friendly and accessible platform should be established in the future.

## Conclusions

In conclusion, an IFPTML model for predicting warfarin blood levels was developed based on longitudinal clinical data. The IFPTML model is a valuable tool that can be used to determine personalized warfarin dosages based on the INR range. This model also considers patient characteristics that affect the warfarin dosage response. The best IFPTML model was generated via the RF algorithm, an ML algorithm that can be used to predict the target INR. GDA can be used to predict the probability of being within the target INR range under different clinical scenarios.

## Ethical approval

This study was approved by the Health Authority Ethics Committee of the First Affiliated Hospital of Soochow University and followed the Declaration of Helsinki.

## Consent

All patients have given written informed consent. Patients were informed that they need to provide blood samples for this study.

## Source of funding

This work was supported by the National Natural Science Foundation of China (grant number 81803628, 8237052); the Jiangsu Provincial Science and Technology Plan Special Fund (BM2023003); the Jiangsu Provincial Medical Key Discipline (grant number ZDXK202247); the Key R&D Program of Jiangsu Province (grant number BE2021644); the Suzhou Health Leading Talent (grant number GSWS2019001); the Talent Project established by the Chinese Pharmaceutical Association Hospital Pharmacy Department (grant number CPA-Z05-ZC-2023-003); the Priority Academic Program Development of the Jiangsu Higher Education Institutes (grant number PAPD), the Suzhou Science and Technology Project under Grant (SKY2023163), grants Basque Government / Eusko Jaurlaritza (IT1558-22), SPRI ELKARTEK grants AIMOFGIF (KK-2022/00032), Ministry of Science and Innovation (PID2022-137365NB-I00), and Eusko Jaurlaritza, LANBIDE, INEVESTIGO Grants, IKERDATA 2022/IKER/000040 funded by NextGenerationEU funds of European Commission.

## Author contribution

L.X.: designing the study, data collection, data analysis and interpretation, writing and revising the paper. S.H.: formal analysis, writing and revising the paper. R.K.S.: formal analysis, writing and revising the paper. Q.Q.: data collection, data interpretation. Y.D.: data collection, data interpretation. L.L.: detecting the concentrations of vitamin K1 and menaquinones K4. X.D.: detecting the genotype. H.B.-B.: data analysis, revising the paper. S.A.: designing the study, data analysis, interpretation, revising the paper. A.D.-S.: interpretation, revising the paper. Y.Z.: data collection, data interpretation. Z.S.: designing the study, data interpretation, revising the paper. B.S.: designing the study, data interpretation, revising the paper. L.M.: designing the study, data interpretation, revising the paper. H.G.-D.: designing the study, data analysis, interpretation, revising the paper.

## Conflicts of interest disclosure

The authors declare no Conflicts of interest.

## Research registration unique identifying number (UIN)

AI-Guided Prediction of Warfarin Blood Levels for Personalized Medicine in Cardiac Surgery Patients (https://www.researchregistry.com/browse-the-registry#home, researchregistry10088)

## Guarantor

Liyan Miao.

## Data availability statement

All the original data in this research are available upon reasonable request from the corresponding authors.

## Provenance and peer review

Not commissioned, externally peer-reviewed

## Supplementary Material

SUPPLEMENTARY MATERIAL
