# Supplementary material for: Machine learning guided prediction of warfarin blood levels for personalized medicine based on clinical longitudinal data from cardiac surgery patients: a prospective observational study
Source: Int J Surg. 2024 Jun 4;110(10):6528–40. doi: 10.1097/JS9.0000000000001734 (PMC11487003; doi:10.1097/JS9.0000000000001734)
Supplement: SUPPLEMENTARY MATERIAL [file js9-110-6528-s002.docx]

**IFPTML data analysis**

Three cases/stages of the IFPTML linear models are shown below.

*Case 1. Classic ML linear model (model of order zero).*

For the classic AI/ML model, we use the original variables as input without regrouping them in distance functions (r = 1, q = 1) and without scaling them with a moving average (α = 0). By applying the previous constraints, the equation of the model can be simplified as follows (Eq. S1):

*Hyperparameters: α = 0, q = 1, r = 1, Parameters:*$a_{c,s}$*= 0,* $a_{k,s}\in R$

$${f\left( v_{ij} \right)}_{calc} = a_{0} +\sum_{k=1}^{kmax} a_{k,s}\cdot v_{k} \left( Eq. S1 \right)$$

*Case 2. First order of the IFPTML model.*

Here, we use as input the original variables without regrouping them into distance functions (r = 1, q = 1), but we perform scaling (α = 1). We transform the original variables into first-order perturbation operations (PTOs) with deviations ∆vk(**s**j) in this scaling. These deviations/perturbations ∆v_k_(**s**_j_) = v_k_ - <v_k_(**s**_j_)> are calculated with a multiconditional moving average <v_k_(**s**_j_)>, which takes different values for different groups of patients according to the subset selected **s**_j_ = **s**_I_, **s**_II_, or **s**_III_, and others. (**Table S2)**. The different possible subsets of patients are stratified according to genetics, genre, treatments, and other factors. Applying the constraints mentioned above, Equations S2 and S3 of the model can be denoted as *follows:*

*Hyperparameters: α = 1, q = 1, r = 1, Parameters:*$a_{c,s}$*=1,* $a_{k,s}\in R$

$${f\left( v_{ij} \right)}_{calc} = a_{0} + a_{1}\cdot{f\left( v_{ij} \right)}_{ref}+\sum_{s=1}^{smax} \sum_{k=1}^{kmax} a_{k,s}\cdot\left[ v_{k}-\left( <v_{k}\left( s_{j} \right)> \right) \right]\left( Eq. S2 \right)$$

$${f\left( v_{ij} \right)}_{calc} = a_{0} + a_{1}\cdot{f\left( v_{ij} \right)}_{ref} +\sum_{s=1}^{smax} \sum_{k=1}^{kmax} a_{k,s}\cdot\Delta v_{k}\left( s_{j} \right) \left( Eq. S3 \right)$$

*Case 3. Second order of the IFPTML model.*

In this subsection, we present an IFPTML model with second-order PTOs. For input, we utilize the original variables; however, we first scale the variables (α = 1) with a multiconditional moving average ∆v_k_(**s**_j_) and then regroup them (r = 1/2, q = 2) into the Euclidean distance (ED) function ǁ∆v_k_(**s**_j_)ǁ, which is calculated as the square root (r = ½) of the sum of the power two (q = 2) for each deviation. Finally, applying the constraints mentioned above, the equation of the model can be denoted as follows (Eqs. S4-S6):

*Hyperparameters: α = 1, q = 2, r = ½, Parameters:* $a_{c,s}\in R,$ $a_{k,s}=1$

$${f\left( v_{ij} \right)}_{calc} = a_{0} + a_{1}\cdot{f\left( v_{ij} \right)}_{ref}+\sum_{s=1}^{smax} a_{c,s}\cdot\left\{ \sum_{k=1}^{kmax} \left[ v_{k}-\left( <v_{k}\left( c_{j} \right)> \right) \right]^{2} \right\}^{1/2}\left( Eq. S4 \right)$$

$${f\left( v_{ij} \right)}_{calc} = a_{0} + a_{1}\cdot{f\left( v_{ij} \right)}_{ref}+\sum_{s=1}^{smax} a_{c,s}\cdot\left\{ \sum_{k=1}^{kmax} {\Delta v_{k}\left( s_{j} \right)}^{2} \right\}^{1/2} \left( Eq. S5 \right)$$

$${f\left( v_{ij} \right)}_{calc} = a_{0} + a_{1}\cdot{f\left( v_{ij} \right)}_{ref}+\sum_{s=1}^{smax} a_{c,s}\cdot\left\| \Delta V_{k}\left( s_{j} \right) \right\|_{c_{j}} \left( Eq. S6 \right)$$

**IF preprocessing and data rearrangement**

The categorical data were grouped according to the physiological characteristics and mechanism of action as follows: sex, smoking status, drinking status, hypertension status, diabetes status, and atrial fibrillation status, labeled **s**_I_ = DemoCat; indication for surgery and valve type as a group, labeled **s**_II_ =TypeCat; genes related to warfarin PK, labeled **s**_III_ = PKGeneCat; genes related to warfarin PD, labeled **s**_IV_ = PDGeneCat; genes related to vitamin K PK, labeled **s**_V_ = VKPKGeneCat; genes related to cytochrome P450 enzyme activity, labeled **s**_VI_ = NRGeneCat; and genes related to the coagulation system, labeled **s**_VII_ = ClotGeneCat.

***IF horizontal process for input labels***

First, we performed IF and reorganization/regrouping of the 62 input labels **s**_j>0_ = [s_1_, s_2_ ...s_62_] into seven different subsets or partitions **s**_j>0_ = **s**_I_, **s**_II_, **s**_III_ …, **s**_VII_. Each partition is constructed as an IF or regrouping of different input labels, *i.e.*, **s**_I_ = [s_1_, s_2_, s_3_, …, s_6_], **s**_II_ = [s_7_, s_8_,], and others. The IFs of the input labels with **s**_j_>0 were divided into partitions **s**_I_, **s**_II_, **s**_III_, … **s**_VII_ according to the expert criteria. We grouped labels of the same type or category of data. For example, **s**_I_ = DemoCat label, **s**_II_ = TypeCat label, **s**_III_ = PKGeneCat label, and others. The names and values of all the input labels and their partitions are shown in **Table 1** or **S1.**

***IF horizontal process for continuous input***

In the IF horizontal process, we performed two steps. First, we calculated the IF of one continuous input variable v_ki_ at the time *vs*. one partition of the input labels **s**_I_, **s**_II_, …., or **s**_VII_. This process enabled the calculation of the first-order PTOs using multicondition deviations ∆v_ki_(**s**_j_). The calculation of these PTOs is explained above. With N_vars_ = 235 input continuous variables (v_ki_) and N_labels_ = 62 input labeling variables (s_j>0_) grouped into seven partitions **s**_j_, we could theoretically calculate a total of N_dev_ = 434 operators ∆v_ki_(s_j_). The input continuous variables, including molecular weight (MW), polar surface area (PSA), Rule of Five (Lipinski) (RO5), and LogD pH 7.4 (LOGP), of the chemical drug were recorded from the ChEMBL database. We used the same criteria to regroup all continuous input variables into partitions of continuous variables **c**_j_. In each of these partitions, **c**_j_ is formed as an IF or regrouping of different input continuous variables v_ki_. Specifically, we grouped r labels of the same type or data category. For instance, **c**_I_ = warfarin daily dosage regime, **c**_II_ = days for warfarin administration prior to INR measurement, **c**_III_ = demography, and others. Afterward, we calculated the first-order PTOs as deviations/perturbations concerning the average of each subset **s**_I_, **s**_II_, and others and second-order PTOs in the form of Euclidean distances ǁ∆v_k_(**s**_j_)ǁ. These parameters measure the distance between the values v_ki_ of the i^th^ patient for all patients with the same values of **s**_j_. Please see the codes, names, and values of the input labels and their partitions in **Table 1** or **S2.**

***IF vertical process and output preprocessing***

The original dataset was formed by one outcome value obtained from patient interviews. The output variables have the values v_ij_ and the labels or names s_0_ = "Within" and **s**_1_ = "Out". All daily dosages of the combined drugs were recorded in grams. Once we assembled all outcome values v_ij_ into one objective variable (column), we preprocessed the values so that all variables were on the same scale. In addition, we converted the original values v_ij_ into an objective function with values f(v_ij_)_obj_ = 1 => when the patient's INR was within the range of 1.8-2.5 or f(v_ij_)_obj_ = 0 => when the patient's INR was outside the range of 1.8-2.5. The values of the objective function are calculated as shown below (Eq. S7):

$${f\left( v_{ij} \right)}_{obj} = IF\left( AND\left( v_{ij}>{cutoff}_{j},d_{j}=1 \right),1,IF\left( AND\left( v_{ij}<{cutoff}_{j},d_{j}=-1 \right),1,0 \right) \right) \left( Eq. S87 \right)$$

The cutoff_j_ values used for the different properties were as follows: INR=1.8-2.5. The parameter desirability d_j_ specifies whether the desired values of v_ij_ are greater than the cutoff d_j_ = 1 or not d_j_ = -1. The reference function f(v_ij_)_ref_ is the first variable in the model. This is equivalent to the expected probability p[f(v_ij_) = 1] with which the parameter (v_ij_) of a sample at the end of the process falls within the specified limits, f(v_ij_)_obs_=1. The risk score depends on the number of patients with an INR in the target range (1.8-2.5) n (f(v_ij_)_obj_= 1) and on the total number of patients n (f(v_ij_)_obs_) with respect to the value of the parameter n(v_ij_)_obs_ for the first interview I_0_ (Eq. S8).

$${f\left( v_{ij} \right)}_{ref} = p[{f\left( v_{ij} \right)}_{obj} = 1]= n({f\left( v_{ij} \right)}_{obj} = 1)/ {n\left( v_{ij} \right)}_{obj} \left( Eq. S98 \right)$$

**Output variable and posterior probability calculation**

In classification models, the output variable f(v_ij_)_calc_ is a scoring function correlated with the probability p[f(v_ij_)_pred_ = 1], where the experimental value of each property measured is predicted v_ij_ to fall within the limits given by the cutoff_j_ values. In addition, the output variable f(v_ij_)_calc_ was discretized to obtain the predicted classification of each case (f(v_ij_)_pred_ = 1) or not (f(v_ij_)_pred_ = 0). The Boolean variable f(v_ij_)_calc_ was compared to the observed classification f(v_ij_)_obs_ for each sample to calculate the specificity (Sp) and sensitivity (Sn) parameters.

The IFPTML model is a classification model generated by LDA. In addition, we can determine the values of f(v_ij_)_calc_ by applying the input values in the model. f(v_ij_)_calc_ is a real value variable (scoring function). Therefore, we must discretize the sample to classify it as f(v_ij_)_pred_ = 1 or f(v_ij_)_pred_ = 0. To do this, we used a sigmoidal function aimed at calculating the a posteriori probabilities p[f(v_ij_)_pred_=1] when a sample was classified as f(v_ij_)_pred_ = 1. This sigmoid function uses a priori probabilities (π_0_ and π_1_) as input. The utility of the prior probabilities in the Bayesian method is to calculate the posterior probabilities. The equation of the sigmoid function of the posterior probabilities is described as follows (Eq. S9):

$$p\left[ {f\left( v_{ij} \right)}_{pred}=1 \right] = \frac{1}{1 + \left( \pi_{0}/\pi_{1} \right)\times exp(-{f(v_{ij})}_{calc})} (Eq. S9)$$

**Table S1**. Category variables are characterized, including each partition **s**_j_ label and the different variables **s**_k_.

| Sets | Name | Variables | Levels/Values | Number of levels |
| --- | --- | --- | --- | --- |
| **s_I_** | DemoCat | s_1_=Gender;  s_2_=Hypertension;  s_3_=Diabetes;  s_4_=Smoke;  s_5_=Drink;  s_6_=Atrial fibrillation | s_1_=Male, female;  s_2_=Hypertension, non-Hypertension;  s_3_=Diabetes, non-Diabetes;  s_4_=Smoke, non-Smoke;  s_5_=Drink, non-Drink;  s_6_=Atrial fibrillation, non-Atrial fibrillation | 6 |
| **s_II_** | TypeCat | s_7_=Type of surgery;  s_8_=Type of valve | s_7_=Aortic, mitral, tricuspid valve replacement, Bentall, wheats;  s_8_=Mechanical, biological, other vascular replacement | 2 |
| **s_III_** | PKGeneCat | s_9_=CYP2C9(rs1057910); s_10_=CYP1A2(rs762551); s_11_=CYP2C(rs12777823); s_12_=CYP2C19(rs4244285); s_13_=CYP2C19(rs4986893);  s_14_=CYP3A4 (rs2242480); s_15_=GATA4(rs12458); s_16_=GATA4(rs13273672); s_17_=GATA4(rs13262643); s_18_=GATA4(rs67176365); s_19_=GATA4(rs13264774); s_20_=GATA4(rs143010652); s_21_=GATA4(rs11785481); s_22_=GATA6(rs10454095); s_23_=UGT1A1(rs887829) | s_9_=AA, AC;  s_10_=AA, AC, CC;  s_11_=AA, AG, GG;  s_12_=AA, AG, GG;  s_13_=AG, GG;  s_14_=CC, CT, TT;  s_15_=AA, AT, TT;  s_16_=CC, CT, TT;  s_17_=CC, CG, GG;  s_18_=AA, AG, GG;  s_19_=CC, CT, TT;  s_20_=AA, AT, TT;  s_21_=CC, CT;  s_22_=CC, CT, TT;  s_23_=CC, CT, TT | 15 |
| **s_IV_** | PDGeneCat | s_24_=VKORC1(rs9923231);  s_25_=CALU (rs2290228); s_26_=EPHX (rs1051740); s_27_=EPHX (rs55798709); s_28_=EPHX (rs1131873); s_29_=GGCX (rs12714145);  s_30_=GGCX (rs699664) | s_24_=AA, AG, GG;  s_25_=AA, AG, GG;  s_26_=CC, CT, TT;  s_27_=AG, GG;  s_28_=AA, AG, GG;  s_29_=CC, CT, TT;  s_30_=CC, CT, TT | 7 |
| **s_V_** | VKPKGeneCat | s_31_=APOB (rs1367117);  s_32_=APOB (rs679899);  s_33_=APOB (rs570877);  s_34_=APOB (rs9282605);  s_35_=APOE (rs7412);  s_36_=CYP4F2(rs2108622) | s_31_=AA, AG, GG;  s_32_=AA, AG, GG;  s_33_=GG, TG, TT;  s_34_=CC, CT;  s_35_=CC, CT;  s_36_=CC, CT, TT | 6 |
| **s_VI_** | NRGeneCat | s_37_=miR-146a(rs2910164); s_38_=NEDD4(rs2288344); s_39_=NR1I2(rs2276707);  s_40_=NR1I2(rs3814055);  s_41_=NR1I2(rs6785049);  s_42_=NR1I3(rs2501873);  s_43_=NR1I3(rs2501874); s_44_=NR1I3(rs117631668); s_45_=NR3C1(rs10052957); s_46_=NR3C1(rs244465);  s_47_=NR3C1(rs33388); s_48_=NR3C1(rs41423247);  s_49_=NR3C1(rs6196);  s_50_=VDR (rs2228570);  s_51_=VDR (rs7975232);  s_52_=VDR (rs731236);  s_53_=VDR (rs11574113);  s_54_=VDR (rs10783218) | s_37_=CC, CG, GG;  s_38_=GG, GT, TT;  s_39_=CC, CT, TT;  s_40_=CC, CT, TT;  s_41_=AA, AG, GG;  s_42_=CC, CT, TT;  s_43_=AA, AG, GG;  s_44_=CC, CG;  s_45_=AA, AG, GG;  s_46_=CC, CT, TT;  s_47_=AA, AT, TT;  s_48_=CC, CG, GG;  s_49_=AA, AG, GG;  s_50_=AA, AG, GG;  s_51_=AA, AC, CC;  s_52_=AA, AG, GG;  s_53_=CC, CG, GG;  s_54_=AG, GG | 18 |
| **s_VII_** | ClotGeneCat | s_55_=CRPG (rs1205);  s_56_=IL1B (rs16944);  s_57_=TGFB1(rs1800470); s_58_=THBD (rs1042580); s_59_=THBD (rs73611750); s_60_=VEGFA (rs833057); s_61_=VEGFA (rs35410204); s_62_=VEGFA (rs866236) | s_55_=CC, CT, TT;  s_56_=AA, AG, GG;  s_57_=AA, AG, GG;  s_58_=CC, CT, TT;  s_59_=AA, AT, TT;  s_60_=GG, GT, TT;  s_61_=CC, CT, TT;  s_62_=CC, CT, TT | 8 |

**Table S2**. The continuous variables' characteristics include the label of each partition **c**_k_ and the different variables v_ki_.

| Sets | Name | Variables | Levels/Values (range) | Number of levels |
| --- | --- | --- | --- | --- |
| **c_I_** | Warfarin daily dosage regime | v_1_=Dose1;  v_2_=Dose2;  v_3_=Dose3;  v_4_=Dose4;  v_5_=Dose5;  v_6_=Dose6;  v_7_=Dose7;  v_8_=Dose8;  v_9_=Dose9;  v_10_=Dose10;  v_11_=Dose11;  v_12_=Dose12;  v_13_=Dose13;  v_14_=Dose14;  v_15_=Dose15;  v_16_=Dose16;  v_17_=Dose17;  v_18_=Dose18;  v_19_=Dose19;  v_20_=Dose20;  v_21_=Dose21;  v_22_=Dose22;  v_23_=Dose23;  v_24_=Dose24;  v_25_=Dose25;  v_26_=Dose26;  v_27_=Dose27;  v_28_=Dose28;  v_29_=Dose29;  v_30_=Dose30;  v_31_=Dose31;  v_32_=Dose32;  v_33_=Dose33;  v_34_=Dose34;  v_35_=Dose35;  v_36_=Dose36;  v_37_=Dose37;  v_38_=Dose38;  v_39_=Dose39;  v_40_=Dose40;  v_41_=Dose41;  v_42_=Dose42;  v_43_=Dose43;  v_44_=Dose44;  v_45_=Dose45;  v_46_=Dose46;  v_47_=Dose47;  v_48_=Dose48;  v_49_=Dose49;  v_50_=Dose50;  v_51_=Dose51;  v_52_=Dose52;  v_53_=Dose53;  v_54_=Dose54;  v_55_=Dose55;  v_56_=Dose56;  v_57_=Dose57;  v_58_=Dose58;  v_59_=Dose59;  v_60_=Dose60;  v_61_=Dose61;  v_62_=Dose62;  v_63_=Dose63 | v_1_= (0.625,7.5);  v_2_= (0.625,6.25);  v_3_= (0.625,6.25);  v_4_= (0.625,5.625);  v_5_= (0.625,5.625);  v_6_= (0.625,5.625);  v_7_= (0.625,5.625);  v_8_= (0.625,5.625);  v_9_= (0.625,5.625);  v_10_= (0.625,5.625);  v_11_= (0.625,5.625);  v_12_= (0.625,5.625);  v_13_= (0.625,5.625);  v_14_= (0.625,5.625);  v_15_= (0.625,5);  v_16_= (0.625,5);  v_17_= (0.625,5);  v_18_= (0.625,5);  v_19_= (0.625,5);  v_20_= (0.625,5);  v_21_= (0.625,5);  v_22_= (0.625,5);  v_23_= (0.625,5);  v_24_= (0.625,5);  v_25_= (0.625,5);  v_26_= (0.625,5);  v_27_= (0.625,5);  v_28_= (0.625,5);  v_29_= (0.625,5);  v_30_= (0.625,4.375);  v_31_= (0.625,5);  v_32_= (0.625,4.375);  v_33_= (0.625,5);  v_34_= (0.625,4.375);  v_35_= (0.625,5);  v_36_= (1.25,3.75);  v_37_= (0.625,3.125);  v_38_= (1.25,3.125);  v_39_= (0.625,3.125);  v_40_= (1.25,3.125);  v_41_= (0.625,3.125);  v_42_= (1.25,3.125);  v_43_= (0.625,3.125);  v_44_= (1.25,3.125);  v_45_= (0.625,3.125);  v_46_= (1.25,3.125);  v_47_= (0.625,3.125);  v_48_= (1.25,3.125);  v_49_= (0.625,3.125);  v_50_= (1.25,1.875);  v_51_= (1.25,1.875);  v_52_= (1.25,1.875);  v_53_= (1.25,1.875);  v_54_= (1.25,1.875);  v_55_= (1.25,1.875);  v_56_= (1.25,1.875);  v_57_= (1.875,1.875);  v_58_= (1.875,1.875);  v_59_= (1.875,1.875);  v_60_= (1.875,1.875);  v_61_= (1.875,1.875);  v_62_= (1.875,1.875);  v_63_= (1.875,1.875) | 63 |
| **c_II_** | Days of warfarin administration prior to INR measurement | v_64_=Time1;  v_65_=Time2;  v_66_=Time3;  v_67_=Time4;  v_68_=Time5;  v_69_=Time6;  v_70_=Time7;  v_71_=Time8;  v_72_=Time9;  v_73_=Time10;  v_74_=Time11;  v_75_=Time12;  v_76_=Time13;  v_77_=Time14;  v_78_=Time15;  v_79_=Time16;  v_80_=Time17;  v_81_=Time18;  v_82_=Time19;  v_83_=Time20;  v_84_=Time21;  v_85_=Time22;  v_86_=Time23;  v_87_=Time24;  v_88_=Time25;  v_89_=Time26;  v_90_=Time27;  v_91_=Time28;  v_92_=Time29;  v_93_=Time30;  v_94_=Time31;  v_95_=Time32;  v_96_=Time33;  v_97_=Time34;  v_98_=Time35;  v_99_=Time36;  v_100_=Time37;  v_101_=Time38;  v_102_=Time39;  v_103_=Time40;  v_104_=Time41;  v_105_=Time42;  v_106_=Time43;  v_107_=Time44;  v_108_=Time45;  v_109_=Time46;  v_110_=Time47;  v_111_=Time48;  v_112_=Time49;  v_113_=Time50;  v_114_=Time51;  v_115_=Time52;  v_116_=Time53;  v_117_=Time54;  v_118_=Time55;  v_119_=Time56;  v_120_=Time57;  v_121_=Time58;  v_122_=Time59;  v_123_=Time60;  v_124_=Time61;  v_125_=Time62;  v_126_=Time63 | v_64_= (0,27.2);  v_65_= (0.59,2.01);  v_66_= (1.58,3.01);  v_67_= (2.56,4.07);  v_68_= (3.56,5.07);  v_69_= (4.54,6.01);  v_70_= (5.51,7.06);  v_71_= (6.56,8.01);  v_72_= (7.56,9.01);  v_73_= (8.74,10.01);  v_74_= (9.74,11.01);  v_75_= (10.7,12.01);  v_76_= (11.7,13.01);  v_77_= (12.6,14.0);  v_78_= (12.7,15.0);  v_79_= (13.7,16.0);  v_80_= (15.7,17.0);  v_81_= (16.7,18.0);  v_82_= (17.7,19.0);  v_83_= (18.7,20.0);  v_84_= (19.7,21.0);  v_85_= (20.7,22.0);  v_86_= (21.7,23.0);  v_87_= (22.7,24.0);  v_88_= (23.7,25.0);  v_89_= (24.7,26.0);  v_90_= (25.7,27.0);  v_91_= (26.1,28.0);  v_92_= (28,29.0);  v_93_= (29,30.0);  v_94_= (30,31.0);  v_95_= (31,32.0);  v_96_= (32,33.0);  v_97_= (33,34.0);  v_98_= (34,35.0);  v_99_= (35,36.0);  v_100_= (36,37.0);  v_101_= (37,38.0);  v_102_= (38,39.0);  v_103_= (39,40.0);  v_104_= (40,40.9);  v_105_= (41,41.9);  v_106_= (42,42.9);  v_107_= (43,43.9);  v_108_= (44,44.9);  v_109_= (45,45.9);  v_110_= (46,46.9);  v_111_= (47,47.9);  v_112_= (48,48.8);  v_113_= (49,49.8);  v_114_= (50,50.8);  v_115_= (51,51.8);  v_116_= (52,52.8);  v_117_= (53,53.8);  v_118_= (54,54.8);  v_119_= (55,55.8);  v_120_= (56,56.8);  v_121_= (57,57.8);  v_122_= (58,58.8);  v_123_= (59,59.8);  v_124_= (60,60.8);  v_125_= (61,61.8);  v_126_= (62,62.8); | 63 |
| **c_III_** | Demography | v_127_=Age;  v_128_=Height;  v_129_=Weight | v_127_= (23.2,83.9);  v_128_= (140,186);  v_129_= (36.3,130); | 3 |
| **c_IV_** | Postoperative days | v_130_=Postoperative days | v_130_= (-42,191); | 1 |
| **c_V_** | Antibiotics | v_131_=Penicillin G sodium;  v_132_=Cefuroxime sodium;  v_133_=Cefuroxime axetil;  v_134_=Cefoperazone sodium;  v_135_=Sulbactam sodium;  v_136_=Ceftazidime;  v_137_=Cefaclor;  v_138_=Imipenem;  v_139_=Cilastatin sodium;  v_140_=Ceftriaxone sodium;  v_141_=Cefdinir;  v_142_=Levofloxacin;  v_143_=Ofloxacin;  v_144_=Moxifloxacin;  v_145_=Ciprofloxacin;  v_146_=Meropenem;  v_147_=Piperacillin sodium;  v_148_=Tazobactam sodium;  v_149_=Ticarcillin disodium;  v_150_=Clavulanate potassium;  v_151_=Azithromycin;  v_152_=Clindamycin phosphate;  v_153_=Tigecycline;  v_154_=Morinidazole | v_131_= (0,11.52);  v_132_= (0,3);  v_133_= (0,1);  v_134_= (0,6);  v_135_= (0,3);  v_136_= (0,6);  v_137_= (0,0.75);  v_138_= (0,3);  v_139_= (0,3);  v_140_= (0,2);  v_141_= (0,0.3);  v_142_= (0,0.5);  v_143_= (0,0.6);  v_144_= (0,2);  v_145_= (0,0.4);  v_146_= (0,3);  v_147_= (0,10.8);  v_148_= (0,2.7);  v_149_= (0,9);  v_150_= (0,0.6);  v_151_= (0,0.5);  v_152_= (0,1.2);  v_153_= (0,150);  v_154_= (0,1) | 24 |
| **c_VI_** | Inhibitor of cytochrome P450 enzyme | v_155_=Amiodarone;  v_156_=Fluconazole;  v_157_=Voriconazole | v_155_= (0,2850);  v_156_= (0,400);  v_157_= (0,800); | 3 |
| **c_VII_** | Celecoxib | v_158_=Celecoxib | v_158_= (0,400); | 1 |
| **c_VIII_** | Diltiazem | v_159_=Diltiazem hydrochloride | v_159_= (0,90); | 1 |
| **c_IX_** | Corticoids | v_160_=Methylprednisolone sodium succinate;  v_161_=Prednisone | v_160_= (0,40);  v_161_= (0,20) | 2 |
| **c_X_** | Diuretic | v_162_=Spironolactone;  v_163_=Furosemide | v_162_= (0,40);  v_163_= (0,80); | 2 |
| **c_XI_** | Omeprazole | v_164_=Omeprazole | v_164_= (0,80); | 1 |
| **c_XII_** | Duphalac | v_165_=Duphalac (Lactulose) | v_165_= (0,20); | 1 |
| **c_XIII_** | Aspirin | v_166_=Aspirin | v_166_= (0,100); | 1 |
| **c_XIV_** | Statin | v_167_=Rosuvastatin calcium;  v_168_=Atorvastatin calcium;  v_169_=Simvastatin | v_167_= (0,10);  v_168_= (0,20);  v_169_= (0,40) | 3 |
| **c_XV_** | Diclofenac | v_170_=Diclofenac sodium | v_170_= (0,100); | 1 |
| **c_XVI_** | Digoxin | v_171_=Digoxin | v_171_= (0,0.125); | 1 |
| **c_XVII_** | Clopidogrel | v_172_=Clopidogrel bisulfate | v_172_= (0,75); | 1 |
| **c_XVIII_** | Vitamin K1 | v_173_=Vitamin K1 | v_173_= (0,20); | 1 |
| **c_XIX_** | Blood cell count | v_174_=Leukocyte Count;  v_175_=Lymphocyte count;  v_176_=Monocyte count;  v_177_=Neutrophil count;  v_178_=Eosinophil count;  v_179_=Basophil count;  v_180_=Lymphocyte percentage;  v_181_=Monocyte percentage;  v_182_=Neutrophil percentage;  v_183_=Eosinophil percentage;  v_184_=Basophil percentage;  v_185_=Red blood cell count;  v_186_=hemoglobin;  v_187_=Hematocrit;  v_188_=Mean corpuscular volume;  v_189_=Mean corpuscular hemoglobin;  v_190_=Mean corpuscular hemoglobin concentration;  v_191_=Red blood cell distribution width;  v_192_=Platelet count;  v_193_=Plateletcrit;  v_194_=Mean platelet volume;  v_195_=Platelet distribution width;  v_196_=C-reactive protein | v_174_= (1.52,27.62);  v_175_= (0.14,7.08);  v_176_= (0.06,1.98);  v_177_= (0.24,25.77);  v_178_= (0,8.56);  v_179_= (0,0.33);  v_180_= (1.32,74.8);  v_181_= (0.51,19.1);  v_182_= (14.1,96.1);  v_183_= (0,56.7);  v_184_= (0,3.70);  v_185_= (2.24,5.68);  v_186_= (66,174);  v_187_= (0.204,0.512);  v_188_= (62.2,109);  v_189_= (18,35.2);  v_190_= (288,364);  v_191_= (11.3,43.8);  v_192_= (18,712);  v_193_= (0.02,0.62);  v_194_= (7.2,16.3);  v_195_= (7.6,22.1);  v_196_= (0.04,286.34) | 23 |
| **c_XX_** | Biochemistry | v_197_=Total bilirubin;  v_198_=Alanine aminotransferase;  v_199_=Glutamic transaminase;  v_200_=γ- Glutamyl transpeptidase;  v_201_=Alkaline phosphatase;  v_202_=Total protein;  v_203_=Albumin;  v_204_=Globulin;  v_205_=Albumin/globulin ratio;  v_206_=Prealbumin;  v_207_=Urea nitrogen;  v_208_=Creatinine;  v_209_=Uric acid;  v_210_=Cystatin C;  v_211_=Blood sugar;  v_212_=Total cholesterol;  v_213_=Triglyceride;  v_214_=High-density cholesterol;  v_215_=Low- density cholesterol;  v_216_=Blood potassium;  v_217_=Blood sodium;  v_218_=Blood chlorine;  v_219_=Blood calcium;  v_220_=Blood phosphorus;  v_221_=Blood magnesium;  v_222_=Lactate dehydrogenase;  v_223_=Creatine kinase;  v_224_=α-Hydroxybutyrate dehydrogenase;  v_225_=Hypersensitivity C-reactive protein | v_197_= (4.3,90.8);  v_198_= (1.9,456.9);  v_199_= (6.9,242);  v_200_= (4.6,503.3);  v_201_= (19.9,324.4);  v_202_= (42.4,87.1);  v_203_= (23.5,51.4);  v_204_= (12.6,48.2);  v_205_= (0.7,2.83);  v_206_= (33.5,398);  v_207_= (2,52.9);  v_208_= (9.79,869.8);  v_209_= (58.6,816);  v_210_= (0.36,8.51);  v_211_= (3.19,21.28);  v_212_= (1.14,8.44);  v_213_= (0.34,19.18);  v_214_= (0.14,1.9);  v_215_= (0.43,5.7);  v_216_= (2.58,6.06);  v_217_= (126.6,162.3);  v_218_= (85.3,120.4);  v_219_= (1.23,3.38);  v_220_= (0.16,3.51);  v_221_= (0.59,1.62);  v_222_= (112.2,2250);  v_223_= (7.9,9492.2);  v_224_= (87.2,933.4);  v_225_= (0.09,22.76) | 29 |
| **c_XXI_** | Vitamin K concentration | v_226_=Vitamin K1 concentration;  v_227_=MK4 concentration | v_226_= (0.033,275.0);  v_227_= (0.012,1.504) | 2 |
| **c_XXII_** | Antibiotics | v_228_=Teicoplanin;  v_229_=Vancomycin | v_228_= (0,1.6);  v_229_= (0,2) | 2 |
| **c_XXIII_** | Human albumin | v_230_=Human albumin | v_230_= (0,40) | 1 |
| **c_XXIV_** | Probiotics | v_231_=Bifidobacterium triple viable powder;  v_232_=Live bacillus licheniformis;  v_233_=Bifidobacterium | v_231_= (0,1890);  v_232_= (0,10);  v_233_= (0,1.4) | 3 |
| **c_XXV_** | Low molecular weight heparin | v_234_=Enoxaparin sodium;  v_235_=Nadroparin calcium | v_234_= (0,8000);  v_235_= (0,8200) | 2 |

**Table S3**. Frequency of additional single nucleotide polymorphisms (SNPs) in the study.

| Gene | SNP (rs) | Genotype | Frequencies (n, %) | Genotype | Frequencies (n, %) | Genotype | Frequencies (n, %) |
| --- | --- | --- | --- | --- | --- | --- | --- |
| APOB | rs1367117 | AA | 5(2%) | AG | 62(25.2%) | GG | 179(72.8%) |
| APOB | rs679899 | AA | 166(67.5%) | AG | 72(29.3%) | GG | 8(3.3%) |
| APOB | rs570877 | GG | 221(89.8%) | TG | 24(9.8%) | TT | 1(0.4%) |
| APOB | rs9282605 | CC | 236(95.9%) | CT | 10(4.1%) | TT | 0(0%) |
| APOE | rs7412 | CC | 214(87%) | CT | 32(13%) | TT | 0(0%) |
| CALU | rs2290228 | AA | 10(4.1%) | AG | 88(35.8%) | GG | 148(60.2%) |
| CRP | rs1205 | CC | 42(17.1%) | CT | 124(50.4%) | TT | 80(32.5%) |
| CYP1A2 | rs762551 | AA | 101(41.1%) | AC | 108(43.9%) | CC | 37(15%) |
| CYP2C | rs12777823 | AA | 22(8.9%) | AG | 106(43.1%) | GG | 118(48%) |
| CYP2C19 | rs4244285 | AA | 22(8.9%) | AG | 107(43.5%) | GG | 117(47.6%) |
| CYP2C19 | rs4986893 | AA | 0(0%) | AG | 18(7.3%) | GG | 229(93.1%) |
| CYP3A4 | rs2242480 | CC | 129(52.4%) | CT | 102(41.5%) | TT | 15(6.1%) |
| EPHX1 | rs1051740 | CC | 43(17.5%) | CT | 121(49.2%) | TT | 82(33.3%) |
| EPHX1 | rs55798709 | AA | 0(0%) | AG | 25(10.2%) | GG | 221(89.8%) |
| EPHX1 | rs1131873 | AA | 28(11.4%) | AG | 99(40.2%) | GG | 119(48.4%) |
| GATA4 | rs12458 | AA | 63(25.6%) | AT | 127(51.6%) | TT | 56(22.8%) |
| GATA4 | rs13273672 | CC | 84(34.1%) | CT | 113(45.9%) | TT | 49(19.9%) |
| GATA4 | rs13262643 | CC | 80(32.5%) | CG | 114(46.3%) | GG | 52(21.1%) |
| GATA4 | rs67176365 | AA | 86(35%) | AG | 114(46.3%) | GG | 46(18.7%) |
| GATA4 | rs13264774 | CC | 52(21.1%) | CT | 113(45.9%) | TT | 81(32.9%) |
| GATA4 | rs143010652 | AA | 1(0.4%) | AT | 6(2.4%) | TT | 239(97.2%) |
| GATA4 | rs11785481 | CC | 237(96.3%) | CT | 9(3.7%) | TT | 0(0%) |
| GATA6 | rs10454095 | CC | 96(39%) | CT | 111(45.1%) | TT | 39(15.9%) |
| GGCX | rs12714145 | CC | 99(40.2%) | CT | 103(41.9%) | TT | 44(17.9%) |
| GGCX | rs699664 | CC | 108(43.9%) | CT | 103(41.9%) | TT | 35(14.2%) |
| IL1B | rs16944 | AA | 61(24.8%) | AG | 123(50%) | GG | 62(25.2%) |
| miR-146a | rs2910164 | CC | 85(34.6%) | CG | 112(45.5%) | GG | 49(19.9%) |
| NEDD4 | rs2288344 | GG | 23(9.3%) | GT | 124(50.4%) | TT | 99(40.2%) |
| NR1I2 | rs2276707 | CC | 67(27.2%) | CT | 115(46.7%) | TT | 64(26%) |
| NR1I2 | rs3814055 | CC | 149(60.6%) | CT | 85(34.6%) | TT | 12(4.9%) |
| NR1I2 | rs6785049 | AA | 43(17.5%) | AG | 116(47.2%) | GG | 87(35.4%) |
| NR1I3 | rs2501873 | CC | 35(14.2%) | CT | 119(48.4%) | TT | 92(37.4%) |
| NR1I3 | rs2501874 | AA | 35(14.2%) | AG | 119(48.4%) | GG | 92(37.4%) |
| NR1I3 | rs117631668 | CC | 234(95.1%) | CG | 12(4.9%) | GG | 0(0%) |
| NR3C1 | rs10052957 | AA | 1(0.4%) | AG | 36(14.6%) | GG | 209(85%) |
| NR3C1 | rs244465 | CC | 141(57.3%) | CT | 84(34.1%) | TT | 21(8.5%) |
| NR3C1 | rs33388 | AA | 10(4.1%) | AT | 83(33.7%) | TT | 153(62.2%) |
| NR3C1 | rs41423247 | CC | 9(3.7%) | CG | 73(29.7%) | GG | 164(66.7%) |
| NR3C1 | rs6196 | AA | 221(89.8%) | AG | 24(9.8%) | GG | 1(0.4%) |
| TGFB1 | rs1800470 | AA | 69(28%) | AG | 113(45.9%) | GG | 64(26%) |
| THBD | rs1042580 | CC | 15(6.1%) | CT | 89(36.2%) | TT | 142(57.7%) |
| THBD | rs73611750 | AA | 2(0.8%) | AT | 19(7.7%) | TT | 225(91.5%) |
| UGT1A1 | rs887829 | CC | 190(77.2%) | CT | 51(20.7%) | TT | 5(2%) |
| VDR | rs2228570 | AA | 48(19.5%) | AG | 122(49.6%) | GG | 76(30.9%) |
| VDR | rs7975232 | AA | 29(11.8%) | AC | 85(34.6%) | CC | 132(53.7%) |
| VDR | rs731236 | CC | 221(89.8%) | CG | 24(9.8%) | GG | 1(0.4%) |
| VDR | rs11574113 | CC | 158(64.2%) | CG | 63(25.6%) | GG | 25(10.2%) |
| VDR | rs10783218 | AA | 0(0%) | AG | 29(11.8%) | GG | 217(88.2%) |
| VEGFA | rs833057 | GG | 85(34.6%) | GT | 126(51.2%) | TT | 35(14.2%) |
| VEGFA | rs35410204 | CC | 3(1.2%) | CT | 64(26%) | TT | 179(72.8%) |
| VEGFA | rs866236 | CC | 56(22.8%) | CT | 122(49.6%) | TT | 68(27.6%) |

**Table S4**. Characteristics of patients' hematological indicators in the study

| Items | Units | Value (Mean ± SD, 95% CI) |
| --- | --- | --- |
| White blood cell | 10^9^/L | 7.88±2.89 (3.79-14.96) |
| Lymphocyte | 10^9^/L | 1.47±0.64 (0.49-2.86) |
| Monocyte | 10^9^/L | 0.56±0.23 (0.23-1.1) |
| Neutrophil | 10^9^/L | 5.61±2.63 (2.18-12.27) |
| Eosinophil | 10^9^/L | 0.21±0.34 (0-0.84) |
| Basophil | 10^9^/L | 0.04±0.03 (0.01-0.1) |
| Red blood cell | 10^12^/L | 3.76±0.52 (2.77-4.84) |
| Hemoglobin | g/L | 111.21±15.53 (84-145.27) |
| Hematocrit | L/L | 0.338±0.045 (0.256-0.431) |
| Mean Corpuscular Volume | fl | 89.98±4.42 (81.5-99) |
| Mean Corpuscular Hemoglobin | pg | 29.63±1.69 (26.2-32.6) |
| Mean corpuscular hemoglobin concentration | g/L | 329.3±9.43 (310-346) |
| Red blood cell distribution width | % | 13.54±1.95 (11.8-18.3) |
| Platelet | 10^9^/L | 236.94±87.47 (90-444) |
| Plateletcrit | % | 0.23±0.07 (0.1-0.39) |
| Mean platelet volume | fl | 9.71±1.31 (7.8-13.2) |
| Platelet distribution width | % | 15.54±1.67 (9.9-16.8) |
| C-reactive protein | mg/L | 15.61±29.03 (0.29-102.36) |

**Table S5**. Patient biochemical indicator characteristics in the study.

| Items | Units | Value (Mean ± SD, 95% CI) |
| --- | --- | --- |
| Total bilirubin | μmol/L | 15.84±9.8 (5.9-41.45) |
| Alanine transaminase | U/L | 39.74±47.2 (7.5-172.4) |
| Aspartate aminotransferase | U/L | 30.76±26.65 (10.9-97) |
| Gamma-glutamyl transpeptidase | U/L | 65.88±61.43 (13.2-274.8) |
| Alkaline phosphatase | U/L | 84.23±40.65 (33.9-196.7) |
| Total protein | g/L | 63.64±6.27 (50.4-75.9) |
| Albumin | g/L | 37.5±4.57 (29.5-46.9) |
| Globulin | g/L | 26.14±4.7 (17.5-35.6) |
| Albumin/Globulin |  | 1.49±0.35 (0.9-2.3) |
| Prealbumin | mg/L | 219.8±53.36 (111.7-324.9) |
| Urea nitrogen | mmol/L | 8.42±4.72 (3.7-18.71) |
| Serum creatinine | μmol/L | 70±44.07 (36.2-144.6) |
| Uric acid | μmol/L | 307.91±116.16 (126-556.9) |
| Cystatin C | mg/L | 1.12±0.55 (0.68-2.28) |
| Glucose | mmol/L | 5.94±1.79 (3.98-10.34) |
| Total cholesterol | mmol/L | 3.91±1.08 (2.16-6.24) |
| Triglyceride | mmol/L | 1.59±1.26 (0.62-3.56) |
| High-density lipoprotein cholesterol | mmol/L | 0.79±0.22 (0.45-1.34) |
| Low-density lipoprotein cholesterol | mmol/L | 2.42±0.87 (0.94-4.54) |
| Potassium | mmol/L | 4.17±0.39 (3.47-5.07) |
| Sodium | mmol/L | 138.23±3.26 (131.7-145.2) |
| Chloride | mmol/L | 101.43±3.69 (94.4-109) |
| Calcium | mmol/L | 2.26±0.12 (2.02-2.49) |
| Phosphorus | mmol/L | 1.31±0.23 (0.87-1.74) |
| Magnesium | mmol/L | 1.03±0.14 (0.79-1.34) |
| Lactic dehydrogenase | U/L | 273.32±106.67 (156.7-462.5) |
| Creatine kinase | U/L | 114.23±314.45 (14.8-735.62) |
| Alpha-hydroxybutyric dehydrogenase | U/L | 223.14±72.88 (120.3-387.6) |
| Hypersensitive C-reactive protein | mg/L | 12.43±4.69 (1.09-15.36) |

**Table S6**. Characteristics of the patients receiving combined medication in the study.

| Combined drug | Value (proportion) |
| --- | --- |
| Cefuroxime sodium | 222(90.2%) |
| Cefuroxime axetil | 20(8.1%) |
| Cefoperazone Sodium and Sulbactam Sodium | 154(62.6%) |
| Ceftazidime | 23(9.3%) |
| Cefaclor | 7(2.8%) |
| Imipenem and Cilastatin sodium | 79(32.1%) |
| Ceftriaxone sodium | 5(2%) |
| Levofloxacin | 83(33.7%) |
| Moxifloxacin | 20(8.1%) |
| Ciprofloxacin | 11(4.5%) |
| Meropenem | 27(11%) |
| Piperacillin and tazobactam | 6(2.4%) |
| Teicoplanin | 29(11.8%) |
| Ticarcillin clavulanate potassium | 34(13.8%) |
| Vancomycin | 31(12.6%) |
| Azithromycin | 5(2%) |
| Clindamycin Phosphate | 8(3.3%) |
| Tigecycline | 19(7.7%) |
| Morinidazole and Sodium Chloride Injection | 7(2.8%) |
| Human albumin | 236(95.9%) |
| Amiodarone | 126(51.2%) |
| Fluconazole | 17(6.9%) |
| Voriconazole | 13(5.3%) |
| Celecoxib | 18(7.3%) |
| Diltiazem | 5(2%) |
| Methylprednisolone | 245(99.6%) |
| Prednisone | 134(54.5%) |
| Spironolactone | 224(91.1%) |
| Furosemide | 239(97.2%) |
| Omeprazole | 242(98.4%) |
| Duphalac | 184(74.8%) |
| Bayaspirin | 20(8.1%) |
| Enoxaparin | 87(35.4%) |
| Vitamin K1 | 42(17.1%) |
| Probiotics Capsules | 46(18.7%) |
| Rosuvastatin | 9(3.7%) |
| Atorvastatin | 25(10.2%) |
| Nadroparin | 6(2.4%) |
| Diclofenac | 7(2.8%) |
| Digoxin | 151(61.4%) |
| Clopidogrel | 18(7.3%) |

**Table S7.** Univariate analysis of input continuous variables for regression analysis

| Continuous variables | Correlation coefficient | *P*-value | Continuous variables | Correlation coefficient | *P*-value |
| --- | --- | --- | --- | --- | --- |
| Dose01 | 0.1727 | 3.004E-23 | Ceftriaxone Sodium | 0.0414 | 0.0180 |
| Dose02 | 0.2130 | 9.232E-35 | Cefdinir | 0.0013 | 0.9395 |
| Dose03 | 0.2749 | 1.295E-57 | Moxifloxacin | 0.0150 | 0.3908 |
| Dose04 | 0.2573 | 1.772E-50 | Ciprofloxacin | 0.0184 | 0.2937 |
| Dose05 | 0.2259 | 5.309E-39 | Meropenem | -0.0209 | 0.2339 |
| Dose06 | 0.2106 | 5.123E-34 | Piperacillin Sodium | -0.0145 | 0.4085 |
| Dose07 | 0.2147 | 2.635E-35 | Tazobactam Sodium | -0.0145 | 0.4085 |
| Dose08 | 0.2030 | 1.095E-31 | Ticarcillin Disodium | 0.0558 | 0.0014 |
| Dose09 | 0.2005 | 6.567E-31 | Clavulanate Potassium | 0.0558 | 0.0014 |
| Dose10 | 0.1928 | 1.158E-28 | Azithromycin | 0.0153 | 0.3818 |
| Dose11 | 0.1940 | 5.067E-29 | Tigecycline | 0.0407 | 0.0200 |
| Dose12 | 0.1651 | 2.271E-21 | Morinidazole | 0.0155 | 0.3772 |
| Dose13 | 0.1697 | 1.75E-22 | Amiodarone | 0.0455 | 0.0093 |
| Dose14 | 0.1224 | 2.374E-12 | Fluconazole | 0.0534 | 0.0023 |
| Age | 0.0182 | 0.2980 | Voriconazole | 0.0645 | 0.0002 |
| Postoperative days | 0.2138 | 4.906E-35 | Celecoxib | 0.0382 | 0.0291 |
| Height | -0.0042 | 0.8121 | Methylprednisolone Sodium Succinate | 0.0112 | 0.5212 |
| Weight | -0.0176 | 0.3154 | Prednisone | 0.0617 | 0.0004 |
| Cefuroxime sodium | -0.1451 | 8.325E-17 | Spironolactone | 0.2211 | 2.185E-37 |
| Cefuroxime axetil | -0.0289 | 0.0993 | Furosemide | 0.1110 | 2.038E-10 |
| Cefoperazone Sodium | 0.0149 | 0.3948 | Omeprazole | -0.0279 | 0.1114 |
| Sulbactam Sodium | 0.0149 | 0.3948 | Duphalac | -0.0172 | 0.3267 |
| Ceftazidime | -0.0476 | 0.0065 | Rosuvastatin Calcium | 0.0726 | 0.0000 |
| Cefaclor | -0.0042 | 0.8092 | Diclofenac Sodium | 0.0370 | 0.0344 |
| Imipenem | -0.0118 | 0.5022 | Vitamin K1 | -0.0318 | 0.0698 |
| Cilastatin Sodium | -0.0118 | 0.5022 | Leukocyte Count | -0.0738 | 2.488E-05 |

**Table S7.** Continued

| Continuous variables | Correlation coefficient | *P*-value | Continuous variables | Correlation coefficient | *P*-value |
| --- | --- | --- | --- | --- | --- |
| Lymphocyte count | 0.0205 | 0.2414 | Albumin | 0.0247 | 0.1581 |
| Monocyte count | -0.0562 | 0.0013 | Globulin | 0.0630 | 0.00032 |
| Neutrophil count | -0.0809 | 3.76E-06 | Albumin/globulin ratio | -0.0354 | 0.0432 |
| Eosinophil count | -0.0040 | 0.8190 | Prealbumin | -0.0339 | 0.0526 |
| Basophil count | 0.0438 | 0.0124 | Urea nitrogen | 0.0013 | 0.9409 |
| Red blood cell count | -0.0687 | 8.653E-05 | Creatinine | -0.0244 | 0.1633 |
| Hemoglobin | -0.0898 | 2.794E-07 | Uric acid | -0.0592 | 0.00073 |
| Hematocrit | -0.0799 | 4.887E-06 | Cystatin C | 0.0214 | 0.2216 |
| Mean corpuscular volume | -0.0181 | 0.3022 | Blood sugar | -0.0809 | 3.687E-06 |
| Mean corpuscular hemoglobin | -0.0447 | 0.0107 | Total cholesterol | -0.0224 | 0.2018 |
| Mean corpuscular hemoglobin concentration | -0.0537 | 0.0022 | Triglyceride | 0.0488 | 0.0053 |
| Red blood cell distribution width | 0.0126 | 0.4715 | High-density cholesterol | -0.1088 | 4.689E-10 |
| Platelet count | 0.1126 | 1.153E-10 | Low- density cholesterol | -0.0105 | 0.54931 |
| Plateletcrit | 0.1022 | 4.981E-09 | Blood potassium | -0.0155 | 0.3757 |
| Mean platelet volume | -0.0817 | 2.975E-06 | Blood sodium | -0.0759 | 1.431E-05 |
| Platelet distribution width | -0.1161 | 2.937E-11 | Blood chlorine | -0.1134 | 8.261E-11 |
| C-reactive protein | -0.0824 | 2.436E-06 | Blood calcium | 0.0364 | 0.0377 |
| Total bilirubin | -0.0994 | 1.271E-08 | Blood phosphorus | 0.0078 | 0.6546 |
| Alanine aminotransferase | -0.0073 | 0.6771 | Blood magnesium | -0.0165 | 0.3470 |
| Glutamic transaminase | -0.0305 | 0.0812 | Lactate dehydrogenase | 0.0177 | 0.3117 |
| γ- Glutamyl transpeptidase | 0.0428 | 0.0144 | Creatine kinase | -0.0702 | 6.038E-05 |
| Alkaline phosphatase | 0.0675 | 0.00011 | α- Hydroxybutyrate dehydrogenase | 0.0435 | 0.01305 |
| Total protein | 0.0661 | 0.00016 | Hypersensitivity C-reactive protein | 0.0996 | 1.211E-08 |

**Table S7.** Continued

| Continuous variables | Correlation coefficient | P-value | Continuous variables | Correlation coefficient | P-value |
| --- | --- | --- | --- | --- | --- |
| Vitamin K1 Concentration | -0.0024 | 0.8900 | Human albumin | -0.1219 | 2.844E-12 |
| MK1 Concentration | -0.0402 | 0.021822 | Bifidobacterium triple viable powder | 0.0079 | 0.6502 |
| Teicoplanin | 0.0341 | 0.05182 | Live Bacillus licheniformis | 0.0116 | 0.5091 |
| Vancomycin | 0.0499 | 0.0044 | Bifidobacterium | -0.0301 | 0.0856 |

**Table S8.** Univariate analysis of input categorical variables for regression analysis

| Categorical variables | Statistics (W or Kruskal-Wallis chi-squared) | *P*-value | Categorical variables | Statistics (W or Kruskal-Wallis chi-squared) | *P*-value |
| --- | --- | --- | --- | --- | --- |
| Penicillin G sodium | 36203 | 0.5656 | EPHX(rs55798709) | 449409 | 0.2142 |
| Levofloxacin | 350802 | 0.5655 | EPHX(rs1131873) | 4.8285 | 0.08943 |
| Diltiazem Hydrochloride | 30273 | 0.1217 | GGCX(rs12714145) | 1.1654 | 0.5584 |
| Aspirin | 253337 | 0.1077 | GGCX(rs699664) | 0.93737 | 0.6258 |
| Atorvastatin Calcium | 309684 | 0.04121 | APOB(rs1367117) | 0.28158 | 0.8687 |
| Simvastatin | 33775 | 0.1093 | APOB(rs679899) | 5.8348 | 0.05408 |
| Digoxin | 1016773 | < 2.2e-16 | APOB(rs570877) | 1.5891 | 0.4518 |
| Clopidogrel Bisulfate | 243414 | 0.1497 | APOB(rs9282605) | 223223 | 0.3185 |
| Enoxaparin Sodium | 93460 | 7.57E-05 | APOE(rs7412) | 604172 | 0.5233 |
| Nadroparin calcium | 21611 | 0.07161 | CYP4F2(rs2108622) | 14.47 | 0.000721 |
| Sex | 1289094 | 0.3095 | miR146a(rs2910164) | 1.6185 | 0.4452 |
| Hypertension | 1281909 | 0.2172 | NEDD4(rs2288344) | 8.9272 | 0.01152 |
| Diabetes | 518040 | 0.3247 | NR1I2(rs2276707) | 0.010177 | 0.9949 |
| Smoke | 713249 | 0.7106 | NR1I2(rs3814055) | 2.751 | 0.2527 |
| Drink | 491166 | 0.9886 | NR1I2(rs6785049) | 0.30544 | 0.8584 |
| Atrial fibrillation | 1111366 | 0.09556 | NR1I3(rs2501873) | 5.8072 | 0.05483 |
| Surgery type | 24.779 | 5.57E-05 | NR1I3(rs2501874) | 5.8072 | 0.05483 |
| Valve Type | 8.4426 | 0.01468 | NR1I3(rs117631668) | 243230 | 0.7264 |
| CYP2C9(rs1057910) | 328024 | 0.000263 | NR3C1(rs10052957) | 0.17937 | 0.9142 |
| CYP1A2(rs762551) | 0.66713 | 0.7164 | NR3C1(rs244465) | 7.1973 | 0.02736 |
| CYP2C(rs12777823) | 4.8417 | 0.08885 | NR3C1(rs33388) | 2.786 | 0.2483 |
| CYP2C19(rs4244285) | 4.6691 | 0.09685 | NR3C1(rs41423247) | 3.1594 | 0.206 |
| CYP2C19(rs4986893) | 334189 | 0.04358 | NR3C1(rs6196) | 0.17597 | 0.9158 |
| CYP3A4(rs2242480) | 1.2311 | 0.5404 | VDR(rs2228570) | 0.071098 | 0.9651 |
| GATA4(rs12458) | 2.7071 | 0.2583 | VDR(rs7975232) | 1.9904 | 0.3696 |
| GATA4(rs13273672) | 2.0717 | 0.3549 | VDR(rs731236) | 0.15201 | 0.9268 |
| GATA4(rs13262643) | 1.4827 | 0.4765 | VDR(rs11574113) | 1.9731 | 0.3729 |
| GATA4(rs67176365) | 0.57821 | 0.7489 | VDR(rs10783218) | 531145 | 0.09996 |
| GATA4(rs13264774) | 1.1662 | 0.5582 | CRPG(rs1205) | 5.8114 | 0.05471 |
| GATA4(rs143010652) | 8.0015 | 0.0183 | IL1B(rs16944) | 2.0675 | 0.3557 |
| GATA4(rs11785481) | 227537 | 0.8834 | TGFB1(rs1800470) | 2.321 | 0.3133 |
| GATA6(rs10454095) | 2.2468 | 0.3252 | THBD(rs1042580) | 7.4775 | 0.02378 |
| UGT1A1(rs887829) | 2.492 | 0.2876 | THBD(rs73611750) | 2.7101 | 0.2579 |
| VKORC1(rs9923231) | 28.35 | 6.98E-07 | VEGFA(rs833057) | 3.6701 | 0.1596 |
| CALU(rs2290228) | 1.4917 | 0.4743 | VEGFA(rs35410204) | 1.4145 | 0.493 |
| EPHX(rs1051740) | 7.1186 | 0.02846 | VEGFA(rs866236) | 7.5213 | 0.02327 |

**Table S9.** Univariate analysis of input continuous variables for classification analysis

| **Continuous variable** | **Statistics U** | ***P*-value** | **Continuous variable** | **Statistics U** | ***P*-value** |
| --- | --- | --- | --- | --- | --- |
| Dose01 | 1069696 | 3.16E-08 | Piperacillin Sodium | 1213197 | 0.173 |
| Dose02 | 1022130 | 1.18E-13 | Tazobactam Sodium | 1213197 | 0.173 |
| Dose03 | 931820 | 1.51E-28 | Ticarcillin disodium | 1192206 | 0.0178 |
| Dose04 | 973794 | 1.57E-23 | Clavulanate Potassium | 1192206 | 0.0178 |
| Dose05 | 1027393 | 1.93E-18 | Azithromycin | 1213530 | 0.0379 |
| Dose06 | 1029342 | 7.74E-19 | Tigecycline | 1189312 | 0.00421 |
| Dose07 | 1031553 | 9.31E-19 | Morinidazole | 1199344 | 0.0148 |
| Dose08 | 1034924 | 7.69E-19 | Amiodarone | 1198241 | 0.578 |
| Dose09 | 1045458 | 2.62E-17 | Fluconazole | 1217660 | 0.149 |
| Dose10 | 1056288 | 1.55E-15 | Voriconazole | 1203786 | 0.424 |
| Dose11 | 1057436 | 1.19E-15 | Celecoxib | 1208378 | 0.902 |
| Dose12 | 1058046 | 4.42E-16 | Methylprednisolone Sodium Succinate | 1224340 | 0.0317 |
| Dose13 | 1062896 | 1.16E-15 | Prednisone | 1175853 | 0.0992 |
| Dose14 | 1072661 | 1.01E-14 | Spironolactone | 995239 | 4.05E-19 |
| Age | 1282886 | 0.00394 | Furosemide | 1176100 | 0.17 |
| Postoperative_days | 802992 | 1.72E-56 | Omeprazole | 1297975 | 5.01E-06 |
| Height | 1181574 | 0.284 | Duphalac | 1280657 | 0.0000267 |
| Weight | 1243334 | 0.18 | Rosuvastatin Calcium | 1205655 | 0.579 |
| Cefuroxime sodium | 1260232 | 5.36E-09 | Diclofenac Sodium | 1195321 | 0.00469 |
| Cefuroxime axetil | 1210979 | 0.661 | Vitamin K1 | 1208700 | 0.971 |
| Cefoperazone Sodium | 1239006 | 0.0604 | Leukocyte Count | 1329945 | 2.37E-06 |
| Sulbactam Sodium | 1239006 | 0.0604 | Lymphocyte count | 1093964 | 7.26E-06 |
| Ceftazidime | 1220614 | 0.0311 | Monocyte count | 1300685 | 0.000346 |
| Cefaclor | 1208636 | 0.879 | Neutrophil count | 1362931 | 1.91E-09 |
| Imipenem | 1238926 | 0.0127 | Eosinophil count | 1115610 | 0.000269 |
| Cilastatin Sodium | 1238926 | 0.0127 | Basophil count | 1125042 | 0.000888 |
| Ceftriaxone Sodium | 1195772 | 0.00343 | Red blood cell count | 1184816 | 0.346 |
| Cefdinir | 1208558 | 0.813 | Hemoglobin | 1198186 | 0.674 |
| Moxifloxacin | 1197365 | 0.0352 | Hematocrit | 1186209 | 0.375 |
| Ciprofloxacin | 1211178 | 0.534 | Mean corpuscular volume | 1264634 | 0.0299 |
| Meropenem | 1223588 | 0.0143 | Mean corpuscular hemoglobin | 1306552 | 0.000141 |

**Table S9.** Continued

| **Continuous variable** | **Statistics U** | ***P*-value** | **Continuous variable** | **Statistics U** | ***P*-value** |
| --- | --- | --- | --- | --- | --- |
| Mean corpuscular hemoglobin concentration | 1295994 | 0.000683 | Total cholesterol | 1186792 | 0.387 |
| Red blood cell distribution width | 1146262 | 0.0144 | Triglyceride | 1153058 | 0.0292 |
| Platelet count | 1059956 | 6.16E-09 | High-density cholesterol | 1227199 | 0.477 |
| Plateletcrit | 1071867 | 8.63E-08 | Low- density cholesterol | 1182056 | 0.294 |
| Mean platelet volume | 1303948 | 0.00021 | Blood potassium | 1201705 | 0.777 |
| Platelet distribution width | 1399215 | 9.88E-14 | Blood sodium | 1230607 | 0.399 |
| C-reactive protein | 1355751 | 1.03E-08 | Blood chlorine | 1283384 | 0.0037 |
| Total bilirubin | 1377256 | 5.23E-11 | Blood calcium | 1092274 | 5.28E-06 |
| Alanine aminotransferase | 1163100 | 0.0736 | Blood phosphorus | 1125426 | 0.00112 |
| Glutamic transaminase | 1190792 | 0.478 | Blood magnesium | 1246142 | 0.147 |
| γ- Glutamyl transpeptidase | 1140956 | 0.00798 | Lactate dehydrogenase | 1198864 | 0.693 |
| Alkaline phosphatase | 1068726 | 4.49E-08 | Creatine kinase | 1324143 | 7.04E-06 |
| Total protein | 1064007 | 1.56E-08 | α- Hydroxybutyrate dehydrogenase | 1189965 | 0.459 |
| Albumin | 1177548 | 0.22 | Hypersensitivity C-reactive protein | 1198900 | 0.641 |
| Globulin | 1055230 | 2.01E-09 | Vitamin K1 Concentration | 1344678 | 1.2E-07 |
| Albumin/globulin ratio | 1302332 | 0.000258 | MK1 Concentration | 1159066 | 0.0516 |
| Prealbumin | 1191138 | 0.487 | Teicoplanin | 1214020 | 0.521 |
| Urea nitrogen | 1295271 | 0.000761 | Vancomycin | 1205230 | 0.672 |
| Creatinine | 1257403 | 0.0589 | Human albumin | 1318950 | 1.31E-13 |
| Uric acid | 1231338 | 0.383 | Bifidobacterium triple viable powder | 1238764 | 0.00146 |
| Cystatin C | 1226138 | 0.503 | Live Bacillus licheniformis | 1208640 | 0.881 |
| Blood sugar | 1330664 | 2.07E-06 | Bifidobacterium | 1211250 | 0.142 |

**Table S10.** Univariate analysis of input categorical variables for classification analysis

| Categorical variable | Statistics (X-squared) | *P*-value | Categorical variable | Statistics (X-squared) | *P*-value |
| --- | --- | --- | --- | --- | --- |
| Penicillin G sodium | 3.1183 | 0.07742 | EPHX(rs55798709) | 0.98242 | 0.3216 |
| Levofloxacin | 1.8896 | 0.1692 | EPHX(rs1131873) | 6.8625 | 0.03235 |
| Diltiazem Hydrochloride | 0.040664 | 0.8402 | GGCX(rs12714145) | 4.6728 | 0.09667 |
| Aspirin | 1.1526 | 0.283 | GGCX(rs699664) | 6.5079 | 0.03862 |
| Atorvastatin Calcium | 4.7144 | 0.02991 | APOB(rs1367117) | 0.26994 | 0.8737 |
| Simvastatin | 2.26E-30 | 1 | APOB(rs679899) | 0.92899 | 0.6285 |
| Digoxin | 71.466 | < 2.2e-16 | APOB(rs570877) | 3.1431 | 0.2077 |
| Clopidogrel Bisulfate | 0.0091806 | 0.9237 | APOB(rs9282605) | 1.2326 | 0.2669 |
| Enoxaparin Sodium | 3.1721 | 0.07491 | APOE(rs7412) | 0.73029 | 0.3928 |
| Nadroparin calcium | 3.66E-27 | 1 | CYP4F2(rs2108622) | 0.43477 | 0.8046 |
| Sex | 0.10374 | 0.7474 | miR146a(rs2910164) | 0.35285 | 0.8383 |
| Hypertension | 1.1067 | 0.2928 | NEDD4(rs2288344) | 2.0809 | 0.3533 |
| Diabetes | 0.45835 | 0.4984 | NR1I2(rs2276707) | 1.366 | 0.5051 |
| Smoke | 0.17809 | 0.673 | NR1I2(rs3814055) | 2.425 | 0.2974 |
| Drink | 0.056112 | 0.8128 | NR1I2(rs6785049) | 1.1283 | 0.5688 |
| Atrial fibrillation | 1.5718 | 0.21 | NR1I3(rs2501873) | 2.5725 | 0.2763 |
| Surgery type | 0.57926 | 0.9653 | NR1I3(rs2501874) | 2.5725 | 0.2763 |
| Valve Type | 11.639 | 0.002969 | NR1I3(rs117631668) | 0.08297 | 0.7733 |
| CYP2C9(rs1057910) | 0.69087 | 0.4059 | NR3C1(rs10052957) | 1.5108 | 0.4698 |
| CYP1A2(rs762551) | 1.7675 | 0.4132 | NR3C1(rs244465) | 2.6278 | 0.2688 |
| CYP2C(rs12777823) | 2.2322 | 0.3275 | NR3C1(rs33388) | 2.3236 | 0.3129 |
| CYP2C19(rs4244285) | 2.3279 | 0.3123 | NR3C1(rs41423247) | 1.5617 | 0.458 |
| CYP2C19(rs4986893) | 1.33E-28 | 1 | NR3C1(rs6196) | 0.91021 | 0.6344 |
| CYP3A4(rs2242480) | 0.30327 | 0.8593 | VDR(rs2228570) | 5.6509 | 0.05928 |
| GATA4(rs12458) | 0.56102 | 0.7554 | VDR(rs7975232) | 1.498 | 0.4728 |
| GATA4(rs13273672) | 0.91936 | 0.6315 | VDR(rs731236) | 2.7498 | 0.2529 |
| GATA4(rs13262643) | 0.61871 | 0.7339 | VDR(rs11574113) | 1.0214 | 0.6001 |
| GATA4(rs67176365) | 1.1126 | 0.5733 | VDR(rs10783218) | 0.009007 | 0.9244 |
| GATA4(rs13264774) | 0.40943 | 0.8149 | CRPG(rs1205) | 0.61579 | 0.735 |
| GATA4(rs143010652) | 1.6192 | 0.445 | IL1B(rs16944) | 3.4204 | 0.1808 |
| GATA4(rs11785481) | 0.32301 | 0.5698 | TGFB1(rs1800470) | 1.9517 | 0.3769 |
| GATA6(rs10454095) | 2.2074 | 0.3316 | THBD(rs1042580) | 1.1885 | 0.552 |
| UGT1A1(rs887829) | 6.5342 | 0.03812 | THBD(rs73611750) | 0.46155 | 0.7939 |
| VKORC1(rs9923231) | 15.545 | 4.21E-04 | VEGFA(rs833057) | 0.82452 | 0.6622 |
| CALU(rs2290228) | 2.1496 | 0.3414 | VEGFA(rs35410204) | 0.20889 | 0.9008 |
| EPHX(rs1051740) | 0.59845 | 0.7414 | VEGFA(rs866236) | 0.93824 | 0.6256 |

**Table S11.** The selected classifiers for different IFPTML algorithms in WEKA software.

| Algorithm | Classifier |
| --- | --- |
| Regression |  |
| LR | weka.classifiers.functions.LinearRegression -S 0 -R 1.0E-8 -num-decimal-places 4 |
| MLP | weka.classifiers.functions.MultilayerPerceptron -L 0.3 -M 0.2 -N 500 -V 0 -S 0 -E 20 -H a |
| SVM | weka.classifiers.functions.SMOreg -C 1.0 -N 0 -V "weka.classifiers.functions.supportVector.RegSMOVmproved -T 0.001 -V -P 1.0E-12 -L 0.001 -W 1" -K "weka.classifiers.functions.supportVector.PolyKernel -E 1.0 -C 250007" |
| KNN | weka.classifiers.lazy.VBk -K 1 -W 0 -A "weka.core.neighboursearch.LinearNNSearch -A \"weka.core.EuclideanDistance -R first-last\"" |
| Bagging | weka.classifiers.meta.Bagging -P 100 -S 1 -num-slots 1 -V 10 -W weka.classifiers.trees.REPTree -- -M 2 -V 0.001 -N 3 -S 1 -L -1 -V 0.0 |
| RF | weka.classifiers.trees.RandomForest -P 100 -V 100 -num-slots 1 -K 0 -M 1.0 -V 0.001 -S 1 |
| **Classification** |  |
| BN | weka.classifiers.bayes.BayesNet -D -Q weka.classifiers.bayes.net.search.local.K2 -- -P 1 -S BAYES -E weka.classifiers.bayes.net.estimate.SimpleEstimator -- -A 0.5 |
| Logistic | weka.classifiers.functions.Logistic -R 1.0E-8 -M -1 -num-decimal-places 4 |
| MLP | weka.classifiers.functions.MultilayerPerceptron -L 0.3 -M 0.2 -N 500 -V 0 -S 0 -E 20 -H a |
| SVM | weka.classifiers.functions.SMO -C 1.0 -L 0.001 -P 1.0E-12 -N 0 -V -1 -W 1 -K "weka.classifiers.functions.supportVector.PolyKernel -E 1.0 -C 250007" -calibrator "weka.classifiers.functions.Logistic -R 1.0E-8 -M -1 -num-decimal-places 4" |
| KNN | weka.classifiers.lazy.VBk -K 1 -W 0 -A "weka.core.neighboursearch.LinearNNSearch -A \"weka.core.EuclideanDistance -R first-last\"" |
| RF | weka.classifiers.trees.RandomForest -P 100 -V 100 -num-slots 1 -K 0 -M 1.0 -V 0.001 -S 1 |

Linear regression: LR; multilayer perceptron: MLP; support vector machine: SVM; K-nearest neighbor: KNN; random forest: RF

**Table S12**. The input variables for warfarin dosage data analysis in the first order of the IFPTML model are called the moving average ∆v_k_(**s**_j_).

| Input variables | Description |
| --- | --- |
| ∆v_01_(**s**_I_) | The deviations ∆v_k_(**s**_j_) of day one warfarin dose prior to INR in the DemoCat group |
| ∆v_02_(**s**_I_) | The deviations ∆v_k_(**s**_j_) of day two warfarin dose prior to INR in the DemoCat group |
| ∆v_03_(**s**_I_) | The deviations ∆v_k_(**s**_j_) of day three warfarin dose prior to INR in the DemoCat group |
| ∆v_04_(**s**_I_) | The deviations ∆v_k_(**s**_j_) of day four warfarin dose prior to INR in the DemoCat group |
| ∆v_05_(**s**_I_) | The deviations ∆v_k_(**s**_j_) of day five warfarin dose prior to INR in the DemoCat group |
| ∆v_06_(**s**_I_) | The deviations ∆v_k_(**s**_j_) of day six warfarin dose prior to INR in the DemoCat group |
| ∆v_07_(**s**_I_) | The deviations ∆v_k_(**s**_j_) of day seven warfarin dose prior to INR in the DemoCat group |
| ∆v_08_(**s**_I_) | The deviations ∆v_k_(**s**_j_) of day eight warfarin dose prior to INR in the DemoCat group |
| ∆v_09_(**s**_I_) | The deviations ∆v_k_(**s**_j_) of day nine warfarin dose prior to INR in the DemoCat group |
| ∆v_10_(**s**_I_) | The deviations ∆v_k_(**s**_j_) of day ten warfarin dose prior to INR in the DemoCat group |
| ∆v_11_(**s**_I_) | The deviations ∆v_k_(**s**_j_) of day 11 warfarin dose prior to INR in the DemoCat group |
| ∆v_12_(**s**_I_) | The deviations ∆v_k_(**s**_j_) of day 12 warfarin dose prior to INR in the DemoCat group |
| ∆v_13_(**s**_I_) | The deviations ∆v_k_(**s**_j_) of day 13 warfarin dose prior to INR in the DemoCat group |
| ∆v_14_(**s**_I_) | The deviations ∆v_k_(**s**_j_) of day 14 warfarin dose prior to INR in the DemoCat group |
| ∆v_29_(MW**s**_I_) | The deviations ∆v_k_(**s**_j_) of molecular weight of amiodarone in the DemoCat group |
| ∆v_30_(MW**s**_I_) | The deviations ∆v_k_(s_j_) of molecular weight of fluconazole in the DemoCat group |
| ∆v_31_(MW**s**_I_) | The deviations ∆v_k_(s_j_) of molecular weight of voriconazole in the DemoCat group |
| ∆v_48_(MW**s**_I_) | The deviations ∆v_k_(s_j_) of molecular weight of rosuvastatin calcium in the DemoCat group |
| ∆v_55_(MW**s**_I_) | The deviations ∆v_k_(s_j_) of molecular weight of vitamin K1 in the DemoCat group |
| ∆v_29_(PSA**s**_I_) | The deviations ∆v_k_(s_j_) of the polar surface area of amiodarone in the DemoCat group |
| ∆v_30_(PSA**s**_I_) | The deviations ∆v_k_(s_j_) of the polar surface area of fluconazole in the DemoCat group |
| ∆v_31_(PSA**s**_I_) | The deviations ∆v_k_(s_j_) of the polar surface area of voriconazole in the DemoCat group |
| ∆v_48_(PSA**s**_I_) | The deviations ∆v_k_(s_j_) of the polar surface area of rosuvastatin calcium in the DemoCat group |
| ∆v_55_(PSA**s**_I_) | The deviations ∆v_k_(s_j_) of the polar surface area of vitamin K1 in the DemoCat group |
| ∆v_02_(ROV**s**_I_) | The deviations ∆v_k_(s_j_) of Ro5 violations (Lipinski) of cefuroxime sodium in the DemoCat group |
| ∆v_03_(ROV**s**_I_) | The deviations ∆v_k_(s_j_) of Ro5 violations (Lipinski) of cefuroxime axetil in the DemoCat group |
| ∆v_29_(ROV**s**_I_) | The deviations ∆v_k_(s_j_) of Ro5 violations (Lipinski) of amiodarone in the DemoCat group |
| ∆v_55_(ROV**s**_I_) | The deviations ∆v_k_(s_j_) of Ro5 violations (Lipinski) of vitamin K1 in the DemoCat group |
| ∆v_12_(LOGP**s**_I_) | The deviations ∆v_k_(s_j_) of CX LogD pH 7.4 of ofloxacin in the DemoCat group |
| ∆v_14_(LOGP**s**_I_) | The deviations ∆v_k_(s_j_) of CX LogD pH 7.4 of ciprofloxacin in the DemoCat group |
| ∆v_29_(LOGP**s**_I_) | The deviations ∆v_k_(s_j_) of CX LogD pH 7.4 of amiodarone in the DemoCat group |
| ∆v_30_(LOGP**s**_I_) | The deviations ∆v_k_(s_j_) of CX LogD pH 7.4 of fluconazole in the DemoCat group |
| ∆v_31_(LOGP**s**_I_) | The deviations ∆v_k_(s_j_) of CX LogD pH 7.4 of voriconazole in the DemoCat group |
| ∆v_35_(LOGP**s**_I_) | The deviations ∆v_k_(s_j_) of CX LogD pH 7.4 of celecoxib in the DemoCat group |
| ∆v_48_(LOGP**s**_I_) | The deviations ∆v_k_(s_j_) of CX LogD pH 7.4 of rosuvastatin calcium in the DemoCat group |
| ∆v_55_(LOGP**s**_I_) | The deviations ∆v_k_(s_j_) of CX LogD pH 7.4 of vitamin K1 in the DemoCat group |
| ∆v_20_(COV**s**_I_) | The deviations ∆v_k_(s_j_) of the covariate for plateletcrit in the DemoCat group |
| ∆v_41_(COV**s**_I_) | The deviations ∆v_k_(s_j_) of the covariate for high-density cholesterol in the DemoCat group |
| ∆v_53_(COV**s**_I_) | The deviations ∆v_k_(s_j_) of the covariate for vitamin K1 concentration in the DemoCat group |
| ∆v_54_(COV**s**_I_) | The deviations ∆v_k_(s_j_) of the covariate for MK4 concentration in the DemoCat group |
| ∆v_01_(**s**_II_) | The deviations ∆v_k_(s_j_) of day one warfarin dose prior to INR in the TypeCat group |
| ∆v_02_(**s**_II_) | The deviations ∆v_k_(s_j_) of day two warfarin dose prior to INR in the TypeCat group |
| ∆v_03_(**s**_II_) | The deviations ∆v_k_(s_j_) of day three warfarin dose prior to INR in the TypeCat group |
| ∆v_04_(**s**_II_) | The deviations ∆v_k_(s_j_) of day four warfarin dose prior to INR in the TypeCat group |
| ∆v_05_(**s**_II_) | The deviations ∆v_k_(s_j_) of day five warfarin dose prior to INR in the TypeCat group |
| ∆v_06_(**s**_II_) | The deviations ∆v_k_(s_j_) of day six warfarin dose prior to INR in the TypeCat group |
| ∆v_07_(**s**_II_) | The deviations ∆v_k_(s_j_) of day seven warfarin dose prior to INR in the TypeCat group |
| ∆v_08_(**s**_II_) | The deviations ∆v_k_(s_j_) of day eight warfarin dose prior to INR in the TypeCat group |
| ∆v_09_(**s**_II_) | The deviations ∆v_k_(s_j_) of day nine warfarin dose prior to INR in the TypeCat group |
| ∆v_10_(**s**_II_) | The deviations ∆v_k_(s_j_) of day ten warfarin dose prior to INR in the TypeCat group |
| ∆v_11_(**s**_II_) | The deviations ∆v_k_(s_j_) of day 11 warfarin dose prior to INR in the TypeCat group |
| ∆v_12_(**s**_II_) | The deviations ∆v_k_(s_j_) of day 12 warfarin dose prior to INR in the TypeCat group |
| ∆v_13_(**s**_II_) | The deviations ∆v_k_(s_j_) of day 13 warfarin dose prior to INR in the TypeCat group |
| ∆v_14_(**s**_II_) | The deviations ∆v_k_(s_j_) of day 14 warfarin dose prior to INR in the TypeCat group |
| ∆v_29_(MW**s**_II_) | The deviations ∆v_k_(s_j_) of molecular weight of amiodarone in the TypeCat group |
| ∆v_30_(MW**s**_II_) | The deviations ∆v_k_(s_j_) of molecular weight of fluconazole in the TypeCat group |
| ∆v_31_(MW**s**_II_) | The deviations ∆v_k_(s_j_) of molecular weight of voriconazole in the TypeCat group |
| ∆v_38_(MW**s**_II_) | The deviations ∆v_k_(s_j_) of molecular weight of prednisone in the TypeCat group |
| ∆v_48_(MW**s**_II_) | The deviations ∆v_k_(s_j_) of molecular weight of rosuvastatin calcium in the TypeCat group |
| ∆v_55_(MW**s**_II_) | The deviations ∆v_k_(s_j_) of molecular weight of vitamin K1 in the TypeCat group |
| ∆v_29_(PSA**s**_II_) | The deviations ∆v_k_(s_j_) of the polar surface area of amiodarone in the TypeCat group |
| ∆v_30_(PSA**s**_II_) | The deviations ∆v_k_(s_j_) of the polar surface area of fluconazole in the TypeCat group |
| ∆v_31_(PSA**s**_II_) | The deviations ∆v_k_(s_j_) of the polar surface area of voriconazole in the TypeCat group |
| ∆v_48_(PSA**s**_II_) | The deviations ∆v_k_(s_j_) of the polar surface area of rosuvastatin calcium in the TypeCat group |
| ∆v_55_(PSA**s**_II_) | The deviations ∆v_k_(s_j_) of the polar surface area of vitamin K1 in the TypeCat group |
| ∆v_02_(ROV**s**_II_) | The deviations ∆v_k_(s_j_) of Ro5 violations (Lipinski) of cefuroxime sodium in the TypeCat group |
| ∆v_03_(ROV**s**_II_) | The deviations ∆v_k_(s_j_) of Ro5 violations (Lipinski) of cefuroxime axetil in the TypeCat group |
| ∆v_29_(ROV**s**_II_) | The deviations ∆v_k_(s_j_) of Ro5 violations (Lipinski) of amiodarone in the TypeCat group |
| ∆v_55_(ROV**s**_II_) | The deviations ∆v_k_(s_j_) of Ro5 violations (Lipinski) of vitamin K1 in the TypeCat group |
| ∆v_03_(LOGP**s**_II_) | The deviations ∆v_k_(s_j_) of CX LogD pH 7.4 of cefuroxime axetil in the TypeCat group |
| ∆v_12_(LOGP**s**_II_) | The deviations ∆v_k_(s_j_) of CX LogD pH 7.4 of ofloxacin in the TypeCat group |
| ∆v_14_(LOGP**s**_II_) | The deviations ∆v_k_(s_j_) of CX LogD pH 7.4 of ciprofloxacin in the TypeCat group |
| ∆v_29_(LOGP**s**_II_) | The deviations ∆v_k_(s_j_) of CX LogD pH 7.4 of amiodarone in the TypeCat group |
| ∆v_30_(LOGP**s**_II_) | The deviations ∆v_k_(s_j_) of CX LogD pH 7.4 of fluconazole in the TypeCat group |
| ∆v_31_(LOGP**s**_II_) | The deviations ∆v_k_(s_j_) of CX LogD pH 7.4 of voriconazole in the TypeCat group |
| ∆v_35_(LOGP**s**_II_) | The deviations ∆v_k_(s_j_) of CX LogD pH 7.4 of celecoxib in the TypeCat group |
| ∆v_48_(LOGP**s**_II_) | The deviations ∆v_k_(s_j_) of CX LogD pH 7.4 of rosuvastatin calcium in the TypeCat group |
| ∆v_55_(LOGP**s**_II_) | The deviations ∆v_k_(s_j_) of CX LogD pH 7.4 of vitamin K1 in the TypeCat group |
| ∆v_20_(COV**s**_II_) | The deviations ∆v_k_(s_j_)of the covariate for plateletcrit in the TypeCat group |
| ∆v_41_(COV**s**_II_) | The deviations ∆v_k_(s_j_) of the covariate for high-density cholesterol in the TypeCat group |
| ∆v_53_(COV**s**_II_) | The deviations ∆v_k_(s_j_) of the covariate for vitamin K1 concentration in the TypeCat group |
| ∆v_54_(COV**s**_II_) | The deviations ∆v_k_(s_j_) of the covariate for MK4 concentration in the TypeCat group |
| ∆v_01_(**s**_III_) | The deviations ∆v_k_(s_j_) of day one warfarin dose prior to INR in the PKGeneCat group |
| ∆v_02_(**s**_III_) | The deviations ∆v_k_(s_j_) of day two warfarin dose prior to INR in the PKGeneCat group |
| ∆v_03_(**s**_III_) | The deviations ∆v_k_(s_j_) of day three warfarin dose prior to INR in the PKGeneCat group |
| ∆v_04_(**s**_III_) | The deviations ∆v_k_(s_j_) of day four warfarin dose prior to INR in the PKGeneCat group |
| ∆v_05_(**s**_III_) | The deviations ∆v_k_(s_j_) of day five warfarin dose prior to INR in the PKGeneCat group |
| ∆v_06_(**s**_III_) | The deviations ∆v_k_(s_j_) of day six warfarin dose prior to INR in the PKGeneCat group |
| ∆v_07_(**s**_III_) | The deviations ∆v_k_(s_j_) of day seven warfarin dose prior to INR in the PKGeneCat group |
| ∆v_08_(**s**_III_) | The deviations ∆v_k_(s_j_) of day eight warfarin dose prior to INR in the PKGeneCat group |
| ∆v_09_(**s**_III_) | The deviations ∆v_k_(s_j_) of day nine warfarin dose prior to INR in the PKGeneCat group |
| ∆v_10_(**s**_III_) | The deviations ∆v_k_(s_j_) of day ten warfarin dose prior to INR in the PKGeneCat group |
| ∆v_11_(**s**_III_) | The deviations ∆v_k_(s_j_) of day 11 warfarin dose prior to INR in the PKGeneCat group |
| ∆v_12_(**s**_III_) | The deviations ∆v_k_(s_j_) of day 12 warfarin dose prior to INR in the PKGeneCat group |
| ∆v_13_(**s**_III_) | The deviations ∆v_k_(s_j_) of day 13 warfarin dose prior to INR in the PKGeneCat group |
| ∆v_14_(**s**_III_) | The deviations ∆v_k_(s_j_) of day 14 warfarin dose prior to INR in the PKGeneCat group |
| ∆v_29_(MW**s**_III_) | The deviations ∆v_k_(s_j_) of molecular weight of amiodarone in the PKGeneCat group |
| ∆v_30_(MW**s**_III_) | The deviations ∆v_k_(s_j_) of molecular weight of fluconazole in the PKGeneCat group |
| ∆v_31_(MW**s**_III_) | The deviations ∆v_k_(s_j_) of molecular weight of voriconazole in the PKGeneCat group |
| ∆v_49_(MW**s**_III_) | The deviations ∆v_k_(s_j_) of molecular weight of atorvastatin calcium in the PKGeneCat group |
| ∆v_55_(MW**s**_III_) | The deviations ∆v_k_(s_j_) of molecular weight of vitamin K1 in the PKGeneCat group |
| ∆v_29_(PSA**s**_III_) | The deviations ∆v_k_(s_j_) of the polar surface area of amiodarone in the PKGeneCat group |
| ∆v_30_(PSA**s**_III_) | The deviations ∆v_k_(s_j_) of the polar surface area of fluconazole in the PKGeneCat group |
| ∆v_31_(PSA**s**_III_) | The deviations ∆v_k_(s_j_) of the polar surface area of voriconazole in the PKGeneCat group |
| ∆v_55_(PSA**s**_III_) | The deviations ∆v_k_(s_j_) of the polar surface area of vitamin K1 in the PKGeneCat group |
| ∆v_02_(ROV**s**_III_) | The deviations ∆v_k_(s_j_) of Ro5 violations (Lipinski) of cefuroxime sodium in the PKGeneCat group |
| ∆v_20_(ROV**s**_III_) | The deviations ∆v_k_(s_j_) of Ro5 violations (Lipinski) of azithromycin in the PKGeneCat group |
| ∆v_22_(ROV**s**_III_) | The deviations ∆v_k_(s_j_) of Ro5 violations (Lipinski) of tigecycline in the PKGeneCat group |
| ∆v_29_(ROV**s**_III_) | The deviations ∆v_k_(s_j_) of Ro5 violations (Lipinski) of amiodarone in the PKGeneCat group |
| ∆v_55_(ROV**s**_III_) | The deviations ∆v_k_(s_j_) of Ro5 violations (Lipinski) of vitamin K1 in the PKGeneCat group |
| ∆v_12_(LOGP**s**_III_) | The deviations ∆v_k_(s_j_) of CX LogD pH 7.4 of ofloxacin in the PKGeneCat group |
| ∆v_13_(LOGP**s**_III_) | The deviations ∆v_k_(s_j_) of CX LogD pH 7.4 of moxifloxacin in the PKGeneCat group |
| ∆v_14_(LOGP**s**_III_) | The deviations ∆v_k_(s_j_) of CX LogD pH 7.4 of ciprofloxacin in the PKGeneCat group |
| ∆v_18_2_(LOGP**s**_III_) | The deviations ∆v_k_(s_j_) of CX LogD pH 7.4 of clavulanate potassium in the PKGeneCat group |
| ∆v_20_(LOGP**s**_III_) | The deviations ∆v_k_(s_j_) of CX LogD pH 7.4 of azithromycin in the PKGeneCat group |
| ∆v_22_(LOGP**s**_III_) | The deviations ∆v_k_(s_j_) of CX LogD pH 7.4 of tigecycline in the PKGeneCat group |
| ∆v_23_(LOGP**s**_III_) | The deviations ∆v_k_(s_j_) of CX LogD pH 7.4 of morinidazole in the PKGeneCat group |
| ∆v_29_(LOGP**s**_III_) | The deviations ∆v_k_(s_j_) of CX LogD pH 7.4 of amiodarone in the PKGeneCat group |
| ∆v_30_(LOGP**s**_III_) | The deviations ∆v_k_(s_j_) of CX LogD pH 7.4 of fluconazole in the PKGeneCat group |
| ∆v_31_(LOGP**s**_III_) | The deviations ∆v_k_(s_j_) of CX LogD pH 7.4 of voriconazole in the PKGeneCat group |
| ∆v_35_(LOGP**s**_III_) | The deviations ∆v_k_(s_j_) of CX LogD pH 7.4 of celecoxib in the PKGeneCat group |
| ∆v_39_(LOGP**s**_III_) | The deviations ∆v_k_(s_j_) of CX LogD pH 7.4 of spironolactone in the PKGeneCat group |
| ∆v_43_(LOGP**s**_III_) | The deviations ∆v_k_(s_j_) of CX LogD pH 7.4 of aspirin in the PKGeneCat group |
| ∆v_50_(LOGP**s**_III_) | The deviations ∆v_k_(s_j_)) of CX LogD pH 7.4 of simvastatin in the PKGeneCat group |
| ∆v_55_(LOGP**s**_III_) | The deviations ∆v_k_(s_j_) of CX LogD pH 7.4 of vitamin K1 in the PKGeneCat group |
| ∆v_6_(COV**s**_III_) | The deviations ∆v_k_(s_j_) of the covariate for basophil count in the PKGeneCat group |
| ∆v_16_(COV**s**_III_) | The deviations ∆v_k_(s_j_) of the covariate for mean corpuscular hemoglobin in the PKGeneCat group |
| ∆v_20_(COV**s**_III_) | The deviations ∆v_k_(s_j_) of the covariate for plateletcrit in the PKGeneCat group |
| ∆v_37_(COV**s**_III_) | The deviations ∆v_k_(s_j_) of the covariate for cystatin C in the PKGeneCat group |
| ∆v_41_(COV**s**_III_) | The deviations ∆v_k_(s_j_) of the covariate for high-density cholesterol in the PKGeneCat group |
| ∆v_53_(COV**s**_III_) | The deviations ∆v_k_(s_j_) of the covariate for vitamin K1 concentration in the PKGeneCat group |
| ∆v_54_(COV**s**_III_) | The deviations ∆v_k_(s_j_) of the covariate for MK4 concentration in the PKGeneCat group |
| ∆v_47_(COVdrug**s**_III_) | The deviations ∆v_k_(s_j_) of the covariate drug for bifidobacterium in the PKGeneCat group |
| ∆v_01_(**s**_IV_) | The deviations ∆v_k_(s_j_) of day one warfarin dose prior to INR in the PDGeneCat group |
| ∆v_02_(**s**_IV_) | The deviations ∆v_k_(s_j_) of day two warfarin dose prior to INR in the PDGeneCat group |
| ∆v_03_(**s**_IV_) | The deviations ∆v_k_(s_j_) of day three warfarin dose prior to INR in the PDGeneCat group |
| ∆v_04_(**s**_IV_) | The deviations ∆v_k_(s_j_) of day four warfarin dose prior to INR in the PDGeneCat group |
| ∆v_05_(**s**_IV_) | The deviations ∆v_k_(s_j_) of day five warfarin dose prior to INR in the PDGeneCat group |
| ∆v_06_(**s**_IV_) | The deviations ∆v_k_(s_j_)of day six warfarin dose prior to INR in the PDGeneCat group |
| ∆v_07_(**s**_IV_) | The deviations ∆v_k_(s_j_) of day seven warfarin dose prior to INR in the PDGeneCat group |
| ∆v_08_(**s**_IV_) | The deviations ∆v_k_(s_j_) of day eight warfarin dose prior to INR in the PDGeneCat group |
| ∆v_09_(**s**_IV_) | The deviations ∆v_k_(s_j_) of day nine warfarin dose prior to INR in the PDGeneCat group |
| ∆v_10_(**s**_IV_) | The deviations ∆v_k_(s_j_) of day ten warfarin dose prior to INR in the PDGeneCat group |
| ∆v_11_(**s**_IV_) | The deviations ∆v_k_(s_j_) of day 11 warfarin dose prior to INR in the PDGeneCat group |
| ∆v_12_(**s**_IV_) | The deviations ∆v_k_(s_j_) of day 12 warfarin dose prior to INR in the PDGeneCat group |
| ∆v_13_(**s**_IV_) | The deviations ∆v_k_(s_j_) of day 13 warfarin dose prior to INR in the PDGeneCat group |
| ∆v_14_(**s**_IV_) | The deviations ∆v_k_(s_j_) of day 14 warfarin dose prior to INR in the PDGeneCat group |
| ∆v_29_(MW**s**_IV_) | The deviations ∆v_k_(s_j_) of molecular weight of amiodarone in the PDGeneCat group |
| ∆v_30_(MW**s**_IV_) | The deviations ∆v_k_(s_j_) of molecular weight of fluconazole in the PDGeneCat group |
| ∆v_31_(MW**s**_IV_) | The deviations ∆v_k_(s_j_) of molecular weight of voriconazole in the PDGeneCat group |
| ∆v_39_(MW**s**_IV_) | The deviations ∆v_k_(s_j_) of molecular weight of spironolactone in the PDGeneCat group |
| ∆v_55_(MW**s**_IV_) | The deviations ∆v_k_(s_j_) of molecular weight of vitamin K1 in the PDGeneCat group |
| ∆v_29_(PSA**s**_IV_) | The deviations ∆v_k_(s_j_) of the polar surface area of amiodarone in the PDGeneCat group |
| ∆v_30_(PSA**s**_IV_) | The deviations ∆v_k_(s_j_) of the polar surface area of fluconazole in the PDGeneCat group |
| ∆v_31_(PSA**s**_IV_) | The deviations ∆v_k_(s_j_) of the polar surface area of voriconazole in the PDGeneCat group |
| ∆v_36_(PSA**s**_IV_) | The deviations ∆v_k_(s_j_) of the polar surface area of diltiazem hydrochloride in the PDGeneCat group |
| ∆v_55_(PSA**s**_IV_) | The deviations ∆v_k_(s_j_) of the polar surface area of vitamin K1 in the PDGeneCat group |
| ∆v_02_(ROV**s**_IV_) | The deviations ∆v_k_(s_j_) of Ro5 violations (Lipinski) of cefuroxime sodium in the PDGeneCat group |
| ∆v_29_(ROV**s**_IV_) | The deviations ∆v_k_(s_j_) of Ro5 violations (Lipinski) of amiodarone in the PDGeneCat group |
| ∆v_55_(ROV**s**_IV_) | The deviations ∆v_k_(s_j_) of Ro5 violations (Lipinski) of vitamin K1 in the PDGeneCat group |
| ∆v_29_(LOGP**s**_IV_) | The deviations ∆v_k_(s_j_) of CX LogD pH 7.4 of amiodarone in the PDGeneCat group |
| ∆v_30_(LOGP**s**_IV_) | The deviations ∆v_k_(s_j_) of CX LogD pH 7.4 of fluconazole in the PDGeneCat group |
| ∆v_31_(LOGP**s**_IV_) | The deviations ∆v_k_(s_j_) of CX LogD pH 7.4 of voriconazole in the PDGeneCat group |
| ∆v_36_(LOGP**s**_IV_) | The deviations ∆v_k_(s_j_) of CX LogD pH 7.4 of diltiazem hydrochloride in the PDGeneCat group |
| ∆v_55_(LOGP**s**_IV_) | The deviations ∆v_k_(s_j_) of CX LogD pH 7.4 of vitamin K1 in the PDGeneCat group |
| ∆v_41_(COV**s**_IV_) | The deviations ∆v_k_(s_j_) of the covariate for high-density cholesterol in the PDGeneCat group |
| ∆v_53_(COV**s**_IV_) | The deviations ∆v_k_(s_j_) of the covariate for vitamin K1 concentration in the PDGeneCat group |
| ∆v_54_(COV**s**_IV_) | The deviations ∆v_k_(s_j_) of the covariate for MK4 concentration in the PDGeneCat group |
| ∆v_19_(COVdrug**s**_IV_) | The deviations ∆v_k_(s_j_) of the covariate drug for vancomycin in the PDGeneCat group |
| ∆v_01_(**s**_V_) | The deviations ∆v_k_(s_j_) of day one warfarin dose prior to INR in the VKPKGeneCat group |
| ∆v_02_(**s**_V_) | The deviations ∆v_k_(s_j_) of day two warfarin dose prior to INR in the VKPKGeneCat group |
| ∆v_03_(**s**_V_) | The deviations ∆v_k_(s_j_) of day three warfarin dose prior to INR in the VKPKGeneCat group |
| ∆v_04_(**s**_V_) | The deviations ∆v_k_(s_j_) of day four warfarin dose prior to INR in the VKPKGeneCat group |
| ∆v_05_(**s**_V_) | The deviations ∆v_k_(s_j_) of day five warfarin dose prior to INR in the VKPKGeneCat group |
| ∆v_06_(**s**_V_) | The deviations ∆v_k_(s_j_) of day six warfarin dose prior to INR in the VKPKGeneCat group |
| ∆v_07_(**s**_V_) | The deviations ∆v_k_(s_j_) of day seven warfarin dose prior to INR in the VKPKGeneCat group |
| ∆v_08_(**s**_V_) | The deviations ∆v_k_(s_j_) of day eight warfarin dose prior to INR in the VKPKGeneCat group |
| ∆v_09_(**s**_V_) | The deviations ∆v_k_(s_j_) of day nine warfarin dose prior to INR in the VKPKGeneCat group |
| ∆v_10_(**s**_V_) | The deviations ∆v_k_(s_j_) of day ten warfarin dose prior to VNR in the VKPKGeneCat group |
| ∆v_11_(**s**_V_) | The deviations ∆v_k_(s_j_) of day 11 warfarin dose prior to VNR in the VKPKGeneCat group |
| ∆v_12_(**s**_V_) | The deviations ∆v_k_(s_j_) of day 12 warfarin dose prior to VNR in the VKPKGeneCat group |
| ∆v_13_(**s**_V_) | The deviations ∆v_k_(s_j_) of day 13 warfarin dose prior to VNR in the VKPKGeneCat group |
| ∆v_14_(**s**_V_) | The deviations ∆v_k_(s_j_) of day 14 warfarin dose prior to VNR in the VKPKGeneCat group |
| ∆v_29_(MW**s**_V_) | The deviations ∆v_k_(s_j_) of molecular weight of amiodarone in the VKPKGeneCat group |
| ∆v_30_(MW**s**_V_) | The deviations ∆v_k_(s_j_) of molecular weight of fluconazole in the VKPKGeneCat group |
| ∆v_31_(MW**s**_V_) | The deviations ∆v_k_(s_j_) of molecular weight of voriconazole in the VKPKGeneCat group |
| ∆v_55_(MW**s**_V_) | The deviations ∆v_k_(s_j_) of molecular weight of vitamin K1 in the VKPKGeneCat group |
| ∆v_29_(PSA**s**_V_) | The deviations ∆v_k_(s_j_) of the polar surface area of amiodarone in the VKPKGeneCat group |
| ∆v_30_(PSA**s**_V_) | The deviations ∆v_k_(s_j_) of the polar surface area of fluconazole in the VKPKGeneCat group |
| ∆v_31_(PSA**s**_V_) | The deviations ∆v_k_(s_j_) of the polar surface area of voriconazole in the VKPKGeneCat group |
| ∆v_36_(PSA**s**_V_) | The deviations ∆v_k_(s_j_) of the polar surface area of diltiazem hydrochloride in the VKPKGeneCat group |
| ∆v_55_(PSA**s**_V_) | The deviations ∆v_k_(s_j_) of the polar surface area of vitamin K1 in the VKPKGeneCat group |
| ∆v_02_(ROV**s**_V_) | The deviations ∆v_k_(s_j_) of Ro5 violations (Lipinski) of cefuroxime sodium in the VKPKGeneCat group |
| ∆v_29_(ROV**s**_V_) | The deviations ∆v_k_(s_j_) of Ro5 violations (Lipinski) of amiodarone in the VKPKGeneCat group |
| ∆v_55_(ROV**s**_V_) | The deviations ∆v_k_(s_j_) of Ro5 violations (Lipinski) of vitamin K1 in the VKPKGeneCat group |
| ∆v_08_(LOGP**s**_V_) | The deviations ∆v_k_(s_j_) of CX LogD pH 7.4 of ceftriaxone sodium in the VKPKGeneCat group |
| ∆v_10_(LOGP**s**_V_) | The deviations ∆v_k_(s_j_) of CX LogD pH 7.4 of cefdinir in the VKPKGeneCat group |
| ∆v_12_(LOGP**s**_V_) | The deviations ∆v_k_(s_j_) of CX LogD pH 7.4 of ofloxacin in the VKPKGeneCat group |
| ∆v_18_2_(LOGP**s**_V_) | The deviations ∆v_k_(s_j_) of CX LogD pH 7.4 of clavulanate potassium in the VKPKGeneCat group |
| ∆v_29_(LOGP**s**_V_) | The deviations ∆v_k_(s_j_)of CX LogD pH 7.4 of amiodarone in the VKPKGeneCat group |
| ∆v_30_(LOGP**s**_V_) | The deviations ∆v_k_(s_j_) of CX LogD pH 7.4 of fluconazole in the VKPKGeneCat group |
| ∆v_31_(LOGP**s**_V_) | The deviations ∆v_k_(s_j_) of CX LogD pH 7.4 of voriconazole in the VKPKGeneCat group |
| ∆v_55_(LOGP**s**_V_) | The deviations ∆v_k_(s_j_) of CX LogD pH 7.4 of vitamin K1 in the VKPKGeneCat group |
| ∆v_41_(COV**s**_V_) | The deviations ∆v_k_(s_j_) of the covariate for high-density cholesterol in the VKPKGeneCat group |
| ∆v_53_(COV**s**_V_) | The deviations ∆v_k_(s_j_) of the covariate for vitamin K1 concentration in the VKPKGeneCat group |
| ∆v_54_(COV**s**_V_) | The deviations ∆v_k_(s_j_) of the covariate for MK4 concentration in the VKPKGeneCat group |
| ∆v_26_((COVdrug**s**_V_) | The deviations ∆v_k_(s_j_) of the covariate drug for human albumin in the VKPKGeneCat group |
| ∆v_01_(**s**_VI_) | The deviations ∆v_k_(s_j_) of day one warfarin dose prior to VNR in the NRGeneCat group |
| ∆v_02_(**s**_VI_) | The deviations ∆v_k_(s_j_) of day two warfarin dose prior to VNR in the NRGeneCat group |
| ∆v_03_(**s**_VI_) | The deviations ∆v_k_(s_j_) of day three warfarin dose prior to VNR in the NRGeneCat group |
| ∆v_04_(**s**_VI_) | The deviations ∆v_k_(s_j_) of day four warfarin dose prior to VNR in the NRGeneCat group |
| ∆v_05_(**s**_VI_) | The deviations ∆v_k_(s_j_) of day five warfarin dose prior to VNR in the NRGeneCat group |
| ∆v_06_(**s**_VI_) | The deviations ∆v_k_(s_j_) of day six warfarin dose prior to VNR in the NRGeneCat group |
| ∆v_07_(**s**_VI_) | The deviations ∆v_k_(s_j_) of day seven warfarin dose prior to VNR in the NRGeneCat group |
| ∆v_08_(**s**_VI_) | The deviations ∆v_k_(s_j_) of day eight warfarin dose prior to VNR in the NRGeneCat group |
| ∆v_09_(**s**_VI_) | The deviations ∆v_k_(s_j_) of day nine warfarin dose prior to VNR in the NRGeneCat group |
| ∆v_10_(**s**_VI_) | The deviations ∆v_k_(s_j_) of day ten warfarin dose prior to VNR in the NRGeneCat group |
| ∆v_11_(**s**_VI_) | The deviations ∆v_k_(s_j_) of day 11 warfarin dose prior to VNR in the NRGeneCat group |
| ∆v_12_(**s**_VI_) | The deviations ∆v_k_(s_j_) of day 12 warfarin dose prior to VNR in the NRGeneCat group |
| ∆v_13_(**s**_VI_) | The deviations ∆v_k_(s_j_) of day 13 warfarin dose prior to VNR in the NRGeneCat group |
| ∆v_14_(**s**_VI_) | The deviations ∆v_k_(s_j_) of day 14 warfarin dose prior to VNR in the NRGeneCat group |
| ∆v_29_(MW**s**_VI_) | The deviations ∆v_k_(s_j_) of molecular weight of amiodarone in the NRGeneCat group |
| ∆v_30_(MW**s**_VI_) | The deviations ∆v_k_(s_j_) of molecular weight of fluconazole in the NRGeneCat group |
| ∆v_31_(MW**s**_VI_) | The deviations ∆v_k_(s_j_) of molecular weight of voriconazole in the NRGeneCat group |
| ∆v_55_(MW**s**_VI_) | The deviations ∆v_k_(s_j_) of molecular weight of vitamin K1 in the NRGeneCat group |
| ∆v_29_(PSA**s**_VI_) | The deviations ∆v_k_(s_j_) of the polar surface area of amiodarone in the NRGeneCat group |
| ∆v_30_(PSA**s**_VI_) | The deviations ∆v_k_(s_j_) of the polar surface area of fluconazole in the NRGeneCat group |
| ∆v_31_(PSA**s**_VI_) | The deviations ∆v_k_(s_j_) of the polar surface area of voriconazole in the NRGeneCat group |
| ∆v_36_(PSA**s**_VI_) | The deviations ∆v_k_(s_j_) of the polar surface area of diltiazem hydrochloride in the NRGeneCat group |
| ∆v_38_(PSA**s**_VI_) | The deviations ∆v_k_(s_j_) of the polar surface area of prednisone in the NRGeneCat group |
| ∆v_55_(PSA**s**_VI_) | The deviations ∆v_k_(s_j_) of the polar surface area of vitamin K1 in the NRGeneCat group |
| ∆v_02_(ROV**s**_VI_) | The deviations ∆v_k_(s_j_) of Ro5 violations (Lipinski) of cefuroxime sodium in the NRGeneCat group |
| ∆v_20_(ROV**s**_VI_) | The deviations ∆v_k_(s_j_) of Ro5 violations (Lipinski) of azithromycin in the NRGeneCat group |
| ∆v_22_(ROV**s**_VI_) | The deviations ∆v_k_(s_j_) of Ro5 violations (Lipinski) of tigecycline in the NRGeneCat group |
| ∆v_29_(ROV**s**_VI_) | The deviations ∆v_k_(s_j_) of Ro5 violations (Lipinski) of amiodarone in the NRGeneCat group |
| ∆v_55_(ROV**s**_VI_) | The deviations ∆v_k_(s_j_) of Ro5 violations (Lipinski) of vitamin K1 in the NRGeneCat group |
| ∆v_05_(LOG**s**_VI_) | The deviations ∆v_k_(s_j_) of CX LogD pH 7.4 of ceftazidime in the NRGeneCat group |
| ∆v_12_(LOGP**s**_VI_) | The deviations ∆v_k_(s_j_) of CX LogD pH 7.4 of ofloxacin in the NRGeneCat group |
| ∆v_13_(LOGP**s**_VI_) | The deviations ∆v_k_(s_j_) of CX LogD pH 7.4 of moxifloxacin in the NRGeneCat group |
| ∆v_18_2_(LOGP**s**_VI_) | The deviations ∆v_k_(s_j_) of CX LogD pH 7.4 of clavulanate potassium in the NRGeneCat group |
| ∆v_20_(LOGP**s**_VI_) | The deviations ∆v_k_(s_j_) of CX LogD pH 7.4 of azithromycin in the NRGeneCat group |
| ∆v_22_(LOGP**s**_VI_) | The deviations ∆v_k_(s_j_) of CX LogD pH 7.4 of tigecycline in the NRGeneCat group |
| ∆v_23_(LOGP**s**_VI_) | The deviations ∆v_k_(s_j_) of CX LogD pH 7.4 of morinidazole in the NRGeneCat group |
| ∆v_29_(LOGP**s**_VI_) | The deviations ∆v_k_(s_j_) of CX LogD pH 7.4 of amiodarone in the NRGeneCat group |
| ∆v_30_(LOGP**s**_VI_) | The deviations ∆v_k_(s_j_) of CX LogD pH 7.4 of fluconazole in the NRGeneCat group |
| ∆v_31_(LOGP**s**_VI_) | The deviations ∆v_k_(s_j_) of CX LogD pH 7.4 of voriconazole in the NRGeneCat group |
| ∆v_35_(LOGP**s**_VI_) | The deviations ∆v_k_(s_j_) of CX LogD pH 7.4 of celecoxib in the NRGeneCat group |
| ∆v_36_(LOGP**s**_VI_) | The deviations ∆v_k_(s_j_) of CX LogD pH 7.4 of diltiazem hydrochloride in the NRGeneCat group |
| ∆v_39_(LOGP**s**_VI_) | The deviations ∆v_k_(s_j_) of CX LogD pH 7.4 of spironolactone in the NRGeneCat group |
| ∆v_50_(LOGP**s**_VI_) | The deviations ∆v_k_(s_j_) of CX LogD pH 7.4 of simvastatin in the NRGeneCat group |
| ∆v_55_(LOGP**s**_VI_) | The deviations ∆v_k_(s_j_) of CX LogD pH 7.4 of vitamin K1 in the NRGeneCat group |
| ∆v_6_(COV**s**_VI_) | The deviations ∆v_k_(s_j_) of the covariate for basophil count in the NRGeneCat group |
| ∆v_16_(COV**s**_VI_) | The deviations ∆v_k_(s_j_) of the covariate for mean corpuscular hemoglobin in the NRGeneCat group |
| ∆v_20_(COV**s**_VI_) | The deviations ∆v_k_(s_j_) of the covariate for plateletcrit in the NRGeneCat group |
| ∆v_37_(COV**s**_VI_) | The deviations ∆v_k_(s_j_) of the covariate for cystatin C in the NRGeneCat group |
| ∆v_38_(COV**s**_VI_) | The deviations ∆v_k_(s_j_) of the covariate for blood sugar in the NRGeneCat group |
| ∆v_41_(COV**s**_VI_) | The deviations ∆v_k_(s_j_) of the covariate for high-density cholesterol in the NRGeneCat group |
| ∆v_53_(COV**s**_VI_) | The deviations ∆v_k_(s_j_) of the covariate for vitamin K1 concentration in the NRGeneCat group |
| ∆v_54_(COV**s**_VI_) | The deviations ∆v_k_(s_j_) of the covariate for MK4 concentration in the NRGeneCat group |
| ∆v_46_(COVdrug**s**_VI_) | The deviations ∆v_k_(s_j_) of covariate drug for live *Bacillus licheniformis* in NRGeneCat group |
| ∆v_01_(**s**_VII_) | The deviations ∆v_k_(s_j_) of day one warfarin dose prior to VNR in the ClotGeneCat group |
| ∆v_02_(**s**_VII_) | The deviations ∆v_k_(s_j_) of day two warfarin dose prior to VNR in the ClotGeneCat group |
| ∆v_03_(**s**_VII_) | The deviations ∆v_k_(s_j_) of day three warfarin dose prior to VNR in the ClotGeneCat group |
| ∆v_04_(**s**_VII_) | The deviations ∆v_k_(s_j_) of day four warfarin dose prior to VNR in the ClotGeneCat group |
| ∆v_05_(**s**_VII_) | The deviations ∆v_k_(s_j_) of day five warfarin dose prior to VNR in the ClotGeneCat group |
| ∆v_06_(**s**_VII_) | The deviations ∆v_k_(s_j_) of day six warfarin dose prior to VNR in the ClotGeneCat group |
| ∆v_07_(**s**_VII_) | The deviations ∆v_k_(s_j_) of day seven warfarin dose prior to VNR in the ClotGeneCat group |
| ∆v_08_(**s**_VII_) | The deviations ∆v_k_(s_j_)of day eight warfarin dose prior to VNR in the ClotGeneCat group |
| ∆v_09_(**s**_VII_) | The deviations ∆v_k_(s_j_) of day nine warfarin dose prior to VNR in the ClotGeneCat group |
| ∆v_10_(**s**_VII_) | The deviations ∆v_k_(s_j_) of day ten warfarin dose prior to VNR in the ClotGeneCat group |
| ∆v_11_(**s**_VII_) | The deviations ∆v_k_(s_j_) of day 11 warfarin dose prior to VNR in the ClotGeneCat group |
| ∆v_12_(**s**_VII_) | The deviations ∆v_k_(s_j_) of day 12 warfarin dose prior to VNR in the ClotGeneCat group |
| ∆v_13_(**s**_VII_) | The deviations ∆v_k_(s_j_) of day 13 warfarin dose prior to VNR in the ClotGeneCat group |
| ∆v_14_(**s**_VII_) | The deviations ∆v_k_(s_j_) of day 14 warfarin dose prior to VNR in the ClotGeneCat group |
| ∆v_29_(MW**s**_VII_) | The deviations ∆v_k_(s_j_) of molecular weight of amiodarone in the ClotGeneCat group |
| ∆v_30_(MW**s**_VII_) | The deviations ∆v_k_(s_j_) of molecular weight of fluconazole in the ClotGeneCat group |
| ∆v_31_(MW**s**_VII_) | The deviations ∆v_k_(s_j_) of molecular weight of voriconazole in the ClotGeneCat group |
| ∆v_48_(MW**s**_VII_) | The deviations ∆v_k_(s_j_) of molecular weight of rosuvastatin calcium in the ClotGeneCat group |
| ∆v_52_(MW**s**_VII_) | The deviations ∆v_k_(s_j_) of molecular weight of diclofenac sodium in the ClotGeneCat group |
| ∆v_55_(MW**s**_VII_) | The deviations ∆v_k_(s_j_) of molecular weight of vitamin K1 in the ClotGeneCat group |
| ∆v_29_(PSA**s**_VII_) | The deviations ∆v_k_(s_j_) of the polar surface area of amiodarone in the ClotGeneCat group |
| ∆v_30_(PSA**s**_VII_) | The deviations ∆v_k_(s_j_) of the polar surface area of fluconazole in the ClotGeneCat group |
| ∆v_31_(PSA**s**_VII_) | The deviations ∆v_k_(s_j_) of the polar surface area of voriconazole in the ClotGeneCat group |
| ∆v_48_(PSA**s**_VII_) | The deviations ∆v_k_(s_j_) of the polar surface area of rosuvastatin calcium in the ClotGeneCat group |
| ∆v_55_(PSA**s**_VII_) | The deviations ∆v_k_(s_j_) of the polar surface area of vitamin K1 in the ClotGeneCat group |
| ∆v_20_(ROV**s**_VII_) | The deviations ∆v_k_(s_j_) of Ro5 violations (Lipinski) of azithromycin in the ClotGeneCat group |
| ∆v_22_(ROV**s**_VII_) | The deviations ∆v_k_(s_j_) of Ro5 violations (Lipinski) of tigecycline in the ClotGeneCat group |
| ∆v_29_(ROV**s**_VII_) | The deviations ∆v_k_(s_j_) of Ro5 violations (Lipinski) of amiodarone in the ClotGeneCat group |
| ∆v_55_(ROV**s**_VII_) | The deviations ∆v_k_(s_j_) of Ro5 violations (Lipinski) of vitamin K1 in the ClotGeneCat group |
| ∆v_12_(LOGP**s**_VII_) | The deviations ∆v_k_(s_j_) of CX LogD pH 7.4 of ofloxacin in the ClotGeneCat group |
| ∆v_18_2_(LOGP**s**_VII_) | The deviations ∆v_k_(s_j_) of CX LogD pH 7.4 of clavulanate potassium in the ClotGeneCat group |
| ∆v_20_(LOGP**s**_VII_) | The deviations ∆v_k_(s_j_) of CX LogD pH 7.4 of azithromycin in the ClotGeneCat group |
| ∆v_22_(LOGP**s**_VII_) | The deviations ∆v_k_(s_j_) of CX LogD pH 7.4 of tigecycline in the ClotGeneCat group |
| ∆v_23_(LOGP**s**_VII_) | The deviations ∆v_k_(s_j_) of CX LogD pH 7.4 of morinidazole in the ClotGeneCat group |
| ∆v_29_(LOGP**s**_VII_) | The deviations ∆v_k_(s_j_) of CX LogD pH 7.4 of amiodarone in the ClotGeneCat group |
| ∆v_30_(LOGP**s**_VII_) | The deviations ∆v_k_(s_j_) of CX LogD pH 7.4 of fluconazole in the ClotGeneCat group |
| ∆v_31_(LOGP**s**_VII_) | The deviations ∆v_k_(s_j_) of CX LogD pH 7.4 of voriconazole in the ClotGeneCat group |
| ∆v_36_(LOGP**s**_VII_) | The deviations ∆v_k_(s_j_) of CX LogD pH 7.4 of diltiazem hydrochloride in the ClotGeneCat group |
| ∆v_48_(LOGP**s**_VII_) | The deviations ∆v_k_(s_j_) of CX LogD pH 7.4 of rosuvastatin calcium in the ClotGeneCat group |
| ∆v_55_(LOGP**s**_VII_) | The deviations ∆v_k_(s_j_) of CX LogD pH 7.4 of vitamin K1 in the ClotGeneCat group |
| ∆v_20_(COV**s**_VII_) | The deviations ∆v_k_(s_j_) of the covariate for plateletcrit in the ClotGeneCatgroup |
| ∆v_22_(COV**s**_VII_) | The deviations ∆v_k_(s_j_) of the covariate for platelet distribution width in the ClotGeneCatgroup |
| ∆v_40_(COV**s**_VII_) | The deviations ∆v_k_(s_j_) of the covariate for triglyceride in the ClotGeneCatgroup |
| ∆v_41_(COV**s**_VII_) | The deviations ∆v_k_(s_j_) of the covariate for high-density cholesterol in the ClotGeneCatgroup |
| ∆v_53_(COV**s**_VII_) | The deviations ∆v_k_(s_j_) of the covariate for vitamin K1 concentration in the ClotGeneCatgroup |
| ∆v_54_(COV**s**_VII_) | The deviations ∆v_k_(s_j_) of the covariate for MK4 concentration in the ClotGeneCatgroup |
| ∆v_47_(COVdrug**s**_VII_) | The deviations ∆v_k_(s_j_) of the covariate drug for bifidobacterium in the ClotGeneCatgroup |

**Table S13**. The input variables for warfarin dosage data analysis in the second order of the IFPTML model are called Euclidean distances ǁ∆v_k_(**s**_j_)ǁ.

| Input variables | Description |
| --- | --- |
| ǁ∆DoseDemoCatǁ | The Euclidean distance of warfarin dosage in the DemoCat group |
| ǁ∆DEMODemoCatǁ | The Euclidean distance of continuous demography variables (such as age, height, weight, and postoperative days) in the DemoCat group |
| ǁ∆MWAntibioticsDemoCatǁ | The Euclidean distance of antibiotics (such as cefuroxime, levofloxacin, and azithromycin) molecular weight in the DemoCat group |
| ǁ∆MWInhibitorDemoCatǁ | The Euclidean distance of inhibitors (such as amiodarone, fluconazole, and voriconazole) of cytochrome P450 enzyme molecular weight in the DemoCat group |
| ǁ∆MWCelecoxibDemoCatǁ | The Euclidean distance of celecoxib molecular weight in the DemoCat group |
| ǁ∆MWDiltiazemDemoCatǁ | The Euclidean distance of diltiazem molecular weight in the DemoCat group |
| ǁ∆MWCorticoidsDemoCatǁ | The Euclidean distance of corticoids (such as methylprednisolone and prednisone) molecular weight in the DemoCat group |
| ǁ∆MWDiureticDemoCatǁ | The Euclidean distance of diuretic (such as spironolactone and furosemide) molecular weight in the DemoCat group |
| ǁ∆MWOmeprazoDemoCatǁ | The Euclidean distance of omeprazole molecular weight in the DemoCat group |
| ǁ∆MWAspirinDemoCatǁ | The Euclidean distance of aspirin molecular weight in the DemoCat group |
| ǁ∆MWStatinDemoCatǁ | The Euclidean distance of statin (such as rosuvastatin, atorvastatin, and simvastatin) molecular weight in the DemoCat group |
| ǁ∆MWDiclofenacDemoCatǁ | The Euclidean distance of diclofenac molecular weight in the DemoCat group |
| ǁ∆MWClopidogrelDemoCatǁ | The Euclidean distance of clopidogrel molecular weight in the DemoCat group |
| ǁ∆MWVK1DemoCatǁ | The Euclidean distance of vitamin K1 molecular weight in the DemoCat group |
| ǁ∆PSAAntibioticsDemoCatǁ | The Euclidean distance of antibiotics (such as cefuroxime, levofloxacin, and azithromycin) polar surface area in the DemoCat group |
| ǁ∆PSAInhibitorDemoCatǁ | The Euclidean distance of inhibitors (such as amiodarone, fluconazole, and voriconazole) of cytochrome P450 enzyme polar surface area in the DemoCat group |
| ǁ∆PSACelecoxibDemoCatǁ | The Euclidean distance of celecoxib polar surface area in the DemoCat group |
| ǁ∆PSADiltiazemDemoCatǁ | The Euclidean distance of diltiazem polar surface area in the DemoCat group |
| ǁ∆PSACorticoidsDemoCatǁ | The Euclidean distance of corticoids (such as methylprednisolone and prednisone) polar surface area in the DemoCat group |
| ǁ∆PSADiureticDemoCatǁ | The Euclidean distance of diuretic (such as spironolactone, furosemide) polar surface area in the DemoCat group |
| ǁ∆PSAOmeprazoDemoCatǁ | The Euclidean distance of omeprazole polar surface area in the DemoCat group |
| ǁ∆PSAAspirinDemoCatǁ | The Euclidean distance of aspirin polar surface area in the DemoCat group |
| ǁ∆PSAStatinDemoCatǁ | The Euclidean distance of statin (such as rosuvastatin, atorvastatin, and simvastatin) polar surface area in the DemoCat group |
| ǁ∆PSADiclofenacDemoCatǁ | The Euclidean distance of diclofenac polar surface area in the DemoCat group |
| ǁ∆PSAClopidogrelDemoCatǁ | The Euclidean distance of clopidogrel polar surface area in the DemoCat group |
| ǁ∆PSAVK1DemoCatǁ | The Euclidean distance of vitamin K1 polar surface area in the DemoCat group |
| ǁ∆ROVAntibioticsDemoCatǁ | The Euclidean distance of antibiotics (such as cefuroxime, levofloxacin, and azithromycin) Ro5 Violations (Lipinski) in the DemoCat group |
| ǁ∆ROVInhibitorDemoCatǁ | The Euclidean distance of inhibitors (such as amiodarone, fluconazole, and voriconazole) of cytochrome P450 enzyme Ro5 Violations (Lipinski) in the DemoCat group |
| ǁ∆ROVStatinDemoCatǁ | The Euclidean distance of statin (such as rosuvastatin, atorvastatin, simvastatin) Ro5 Violations (Lipinski) in the DemoCat group |
| ǁ∆ROVVK1DemoCatǁ | The Euclidean distance of vitamin K1 Ro5 Violations (Lipinski) in the DemoCat group |
| ǁ∆LOGPAntibioticsDemoCatǁ | The Euclidean distance of antibiotics (such as cefuroxime, levofloxacin, and azithromycin) CX LogD pH 7.4 in the DemoCat group |
| ǁ∆LOGPInhibitorDemoCatǁ | The Euclidean distance of inhibitors (such as amiodarone, fluconazole, and voriconazole) of cytochrome P450 enzyme CX LogD pH 7.4 in the DemoCat group |
| ǁ∆LOGPCelecoxibDemoCatǁ | The Euclidean distance of celecoxib CX LogD pH 7.4 in the DemoCat group |
| ǁ∆LOGPDiltiazemDemoCatǁ | The Euclidean distance of diltiazem CX LogD pH 7.4 in the DemoCat group |
| ǁ∆LOGPCorticoidsDemoCatǁ | The Euclidean distance of corticoids (such as methylprednisolone, prednisone) CX LogD pH 7.4 in the DemoCat group |
| ǁ∆LOGPDiureticDemoCatǁ | The Euclidean distance of diuretic (such as spironolactone, furosemide) CX LogD pH 7.4 in the DemoCat group |
| ǁ∆LOGPOmeprazoDemoCatǁ | The Euclidean distance of omeprazole CX LogD pH 7.4 in the DemoCat group |
| ǁ∆LOGPAspirinDemoCatǁ | The Euclidean distance of aspirin CX LogD pH 7.4 in the DemoCat group |
| ǁ∆LOGPStatinDemoCatǁ | The Euclidean distance of statin (such as rosuvastatin, atorvastatin, simvastatin) CX LogD pH 7.4 in the DemoCat group |
| ǁ∆LOGPDiclofenacDemoCatǁ | The Euclidean distance of diclofenac CX LogD pH 7.4 in the DemoCat group |
| ǁ∆LOGPClopidogrelDemoCatǁ | The Euclidean distance of clopidogrel CX LogD pH 7.4 in the DemoCat group |
| ǁ∆LOGPVK1DemoCatǁ | The Euclidean distance of vitamin K1 CX LogD pH 7.4 in the DemoCat group |
| ǁ∆Blood_cell_countDemoCatǁ | The Euclidean distance of blood cell counts in the DemoCat group |
| ǁ∆BiochemistryDemoCatǁ | The Euclidean distance of blood biochemistry in the DemoCat group |
| ǁ∆VKConDemoCatǁ | The Euclidean distance of vitamin K concentration in the DemoCat group |
| ǁ∆AntibioticsDemoCatǁ | The Euclidean distance of antibiotics (such as vancomycin and teicoplanin) in the DemoCat group |
| ǁ∆AlbuminDemoCatǁ | The Euclidean distance of human albumin in the DemoCat group |
| ǁ∆ProbioticsDemoCatǁ | The Euclidean distance of probiotics (such as bifidobacterium triple viable powder, live *Bacillus* *licheniformis*, and bifidobacterium) in the DemoCat group |
| ǁ∆LMWHDemoCatǁ | The Euclidean distance of low molecular weight heparin (such as enoxaparin and nadroparin) in the DemoCat group |
| ǁ∆DoseTypeCatǁ | The Euclidean distance of warfarin dosage in the TypeCat group |
| ǁ∆DEMOTypeCatǁ | The Euclidean distance of continuous demography variables (such as age, height, weight, and postoperative days) in the TypeCat group |
| ǁ∆MWAntibioticsTypeCatǁ | The Euclidean distance of antibiotics (such as cefuroxime, levofloxacin, and azithromycin) molecular weight in the TypeCat group |
| ǁ∆MWInhibitorTypeCatǁ | The Euclidean distance of inhibitors (such as amiodarone, fluconazole, and voriconazole) of cytochrome P450 enzyme molecular weight in the TypeCat group |
| ǁ∆MWCelecoxibTypeCatǁ | The Euclidean distance of celecoxib molecular weight in the TypeCat group |
| ǁ∆MWDiltiazemTypeCatǁ | The Euclidean distance of diltiazem molecular weight in the TypeCat group |
| ǁ∆MWCorticoidsTypeCatǁ | The Euclidean distance of corticoids (such as methylprednisolone and prednisone) molecular weight in the TypeCat group |
| ǁ∆MWDiureticTypeCatǁ | The Euclidean distance of diuretic (such as spironolactone and furosemide) molecular weight in the TypeCat group |
| ǁ∆MWOmeprazoTypeCatǁ | The Euclidean distance of omeprazole molecular weight in the TypeCat group |
| ǁ∆MWAspirinTypeCatǁ | The Euclidean distance of aspirin molecular weight in the TypeCat group |
| ǁ∆MWStatinTypeCatǁ | The Euclidean distance of statin (such as rosuvastatin, atorvastatin, and simvastatin) molecular weight in the TypeCat group |
| ǁ∆MWDiclofenacTypeCatǁ | The Euclidean distance of diclofenac molecular weight in the TypeCat group |
| ǁ∆MWClopidogrelTypeCatǁ | The Euclidean distance of clopidogrel molecular weight in the TypeCat group |
| ǁ∆MWVK1TypeCatǁ | The Euclidean distance of vitamin K1 molecular weight in the TypeCat group |
| ǁ∆PSAAntibioticsTypeCatǁ | The Euclidean distance of antibiotics (such as cefuroxime, levofloxacin, and azithromycin) polar surface area in the TypeCat group |
| ǁ∆PSAInhibitorTypeCatǁ | The Euclidean distance of inhibitors (such as amiodarone, fluconazole, and voriconazole) of cytochrome P450 enzyme polar surface area in the TypeCat group |
| ǁ∆PSACelecoxibTypeCatǁ | The Euclidean distance of celecoxib polar surface area in the TypeCat group |
| ǁ∆PSADiltiazemTypeCatǁ | The Euclidean distance of diltiazem polar surface area in the TypeCat group |
| ǁ∆PSACorticoidsTypeCatǁ | The Euclidean distance of corticoids (such as methylprednisolone and prednisone) polar surface area in the TypeCat group |
| ǁ∆PSADiureticTypeCatǁ | The Euclidean distance of diuretic (such as spironolactone and furosemide) polar surface area in the TypeCat group |
| ǁ∆PSAOmeprazoTypeCatǁ | The Euclidean distance of omeprazole polar surface area in the TypeCat group |
| ǁ∆PSAAspirinTypeCatǁ | The Euclidean distance of aspirin polar surface area in the TypeCat group |
| ǁ∆PSAStatinTypeCatǁ | The Euclidean distance of statin (such as rosuvastatin, atorvastatin, and simvastatin) polar surface area in the TypeCat group |
| ǁ∆PSADiclofenacTypeCatǁ | The Euclidean distance of diclofenac polar surface area in the TypeCat group |
| ǁ∆PSAClopidogrelTypeCatǁ | The Euclidean distance of clopidogrel polar surface area in the TypeCat group |
| ǁ∆PSAVK1TypeCatǁ | The Euclidean distance of vitamin K1 polar surface area in the TypeCat group |
| ǁ∆ROVAntibioticsTypeCatǁ | The Euclidean distance of antibiotics (such as cefuroxime, levofloxacin, and azithromycin) Ro5 Violations (Lipinski) in the TypeCat group |
| ǁ∆ROVInhibitorTypeCatǁ | The Euclidean distance of inhibitors (such as amiodarone, fluconazole, and voriconazole) of cytochrome P450 enzyme Ro5 Violations (Lipinski) in the TypeCat group |
| ǁ∆ROVStatinTypeCatǁ | The Euclidean distance of statin (such as rosuvastatin, atorvastatin, and simvastatin) Ro5 Violations (Lipinski) in the TypeCat group |
| ǁ∆ROVVK1TypeCatǁ | The Euclidean distance of vitamin K1 Ro5 Violations (Lipinski) in the TypeCat group |
| ǁ∆LOGPAntibioticsTypeCatǁ | The Euclidean distance of antibiotics (such as cefuroxime, levofloxacin, and azithromycin) CX LogD pH 7.4 in the TypeCat group |
| ǁ∆LOGPInhibitorTypeCatǁ | The Euclidean distance of inhibitors (such as amiodarone, fluconazole, and voriconazole) of cytochrome P450 enzyme CX LogD pH 7.4 in the TypeCat group |
| ǁ∆LOGPCelecoxibTypeCatǁ | The Euclidean distance of celecoxib CX LogD pH 7.4 in the TypeCat group |
| ǁ∆LOGPDiltiazemTypeCatǁ | The Euclidean distance of diltiazem CX LogD pH 7.4 in the TypeCat group |
| ǁ∆LOGPCorticoidsTypeCatǁ | The Euclidean distance of corticoids (such as methylprednisolone and prednisone) CX LogD pH 7.4 in the TypeCat group |
| ǁ∆LOGPDiureticTypeCatǁ | The Euclidean distance of diuretic (such as spironolactone and furosemide) CX LogD pH 7.4 in the TypeCat group |
| ǁ∆LOGPOmeprazoTypeCatǁ | The Euclidean distance of omeprazole CX LogD pH 7.4 in the TypeCat group |
| ǁ∆LOGPAspirinTypeCatǁ | The Euclidean distance of aspirin CX LogD pH 7.4 in the TypeCat group |
| ǁ∆LOGPStatinTypeCatǁ | The Euclidean distance of statin (such as rosuvastatin, atorvastatin, and simvastatin) CX LogD pH 7.4 in the TypeCat group |
| ǁ∆LOGPDiclofenacTypeCatǁ | The Euclidean distance of diclofenac CX LogD pH 7.4 in the TypeCat group |
| ǁ∆LOGPClopidogrelTypeCatǁ | The Euclidean distance of clopidogrel CX LogD pH 7.4 in the TypeCat group |
| ǁ∆LOGPVK1TypeCatǁ | The Euclidean distance of vitamin K1 CX LogD pH 7.4 in the TypeCat group |
| ǁ∆Blood_cell_countTypeCatǁ | The Euclidean distance of blood cell counts in the TypeCat group |
| ǁ∆BiochemistryTypeCatǁ | The Euclidean distance of blood biochemistry in the TypeCat group |
| ǁ∆VKConTypeCatǁ | The Euclidean distance of vitamin K concentration in the TypeCat group |
| ǁ∆AntibioticsTypeCatǁ | The Euclidean distance of antibiotics (such as vancomycin and teicoplanin) in the TypeCat group |
| ǁ∆AlbuminTypeCatǁ | The Euclidean distance of human albumin in the TypeCat group |
| ǁ∆ProbioticsTypeCatǁ | The Euclidean distance of probiotics (such as bifidobacterium triple viable powder, live *Bacillus licheniformis*, and bifidobacterium) in the TypeCat group |
| ǁ∆LMWHTypeCatǁ | The Euclidean distance of low molecular weight heparin (such as enoxaparin, nadroparin) in the TypeCat group |
| ǁ∆DosePKGeneCatǁ | The Euclidean distance of warfarin dosage in the PKGeneCat group |
| ǁ∆DEMOPKGeneCatǁ | The Euclidean distance of continuous demography variables (such as age, height, weight, and postoperative days) in the PKGeneCat group |
| ǁ∆MWAntibioticsPKGeneCatǁ | The Euclidean distance of antibiotics (such as cefuroxime, levofloxacin, and azithromycin) molecular weight in the PKGeneCat group |
| ǁ∆MWInhibitorPKGeneCatǁ | The Euclidean distance of inhibitors (such as amiodarone, fluconazole, and voriconazole) of cytochrome P450 enzyme molecular weight in the PKGeneCat group |
| ǁ∆MWCelecoxibPKGeneCatǁ | The Euclidean distance of celecoxib molecular weight in the PKGeneCat group |
| ǁ∆MWDiltiazemPKGeneCatǁ | The Euclidean distance of diltiazem molecular weight in the PKGeneCat group |
| ǁ∆MWCorticoidsPKGeneCatǁ | The Euclidean distance of corticoids (such as methylprednisolone and prednisone) molecular weight in the PKGeneCat group |
| ǁ∆MWDiureticPKGeneCatǁ | The Euclidean distance of diuretic (such as spironolactone and furosemide) molecular weight in the PKGeneCat group |
| ǁ∆MWOmeprazoPKGeneCatǁ | The Euclidean distance of omeprazole molecular weight in the PKGeneCat group |
| ǁ∆MWAspirinPKGeneCatǁ | The Euclidean distance of aspirin molecular weight in the PKGeneCat group |
| ǁ∆MWStatinPKGeneCatǁ | The Euclidean distance of statin (such as rosuvastatin, atorvastatin, and simvastatin) molecular weight in the PKGeneCat group |
| ǁ∆MWDiclofenacPKGeneCatǁ | The Euclidean distance of diclofenac molecular weight in the PKGeneCat group |
| ǁ∆MWClopidogrelPKGeneCatǁ | The Euclidean distance of clopidogrel molecular weight in the PKGeneCat group |
| ǁ∆MWVK1PKGeneCatǁ | The Euclidean distance of vitamin K1 molecular weight in the PKGeneCat group |
| ǁ∆PSAAntibioticsPKGeneCatǁ | The Euclidean distance of antibiotics (such as cefuroxime, levofloxacin, and azithromycin) polar surface area in the PKGeneCat group |
| ǁ∆PSAInhibitorPKGeneCatǁ | The Euclidean distance of inhibitors (such as amiodarone, fluconazole, and voriconazole) of cytochrome P450 enzyme polar surface area in PKGeneCat group |
| ǁ∆PSACelecoxibPKGeneCatǁ | The Euclidean distance of celecoxib polar surface area in the PKGeneCat group |
| ǁ∆PSADiltiazemPKGeneCatǁ | The Euclidean distance of diltiazem polar surface area in the PKGeneCat group |
| ǁ∆PSACorticoidsPKGeneCatǁ | The Euclidean distance of corticoids (such as methylprednisolone and prednisone) polar surface area in the PKGeneCat group |
| ǁ∆PSADiureticPKGeneCatǁ | The Euclidean distance of diuretic (such as spironolactone and furosemide) polar surface area in the PKGeneCat group |
| ǁ∆PSAOmeprazoPKGeneCatǁ | The Euclidean distance of omeprazole polar surface area in the PKGeneCat group |
| ǁ∆PSAAspirinPKGeneCatǁ | The Euclidean distance of aspirin polar surface area in the PKGeneCat group |
| ǁ∆PSAStatinPKGeneCatǁ | The Euclidean distance of statin (such as rosuvastatin, atorvastatin, and simvastatin) polar surface area in the PKGeneCat group |
| ǁ∆PSADiclofenacPKGeneCatǁ | The Euclidean distance of diclofenac polar surface area in the PKGeneCat group |
| ǁ∆PSAClopidogrelPKGeneCatǁ | The Euclidean distance of clopidogrel polar surface area in the PKGeneCat group |
| ǁ∆PSAVK1PKGeneCatǁ | The Euclidean distance of vitamin K1 polar surface area in the PKGeneCat group |
| ǁ∆ROVAntibioticsPKGeneCatǁ | The Euclidean distance of antibiotics (such as cefuroxime, levofloxacin, and azithromycin) Ro5 Violations (Lipinski) in the PKGeneCat group |
| ǁ∆ROVInhibitorPKGeneCatǁ | The Euclidean distance of inhibitors (such as amiodarone, fluconazole, and voriconazole) of cytochrome P450 enzyme Ro5 Violations (Lipinski) in the PKGeneCat group |
| ǁ∆ROVStatinPKGeneCatǁ | The Euclidean distance of statin (such as rosuvastatin, atorvastatin, and simvastatin) Ro5 Violations (Lipinski) in the PKGeneCat group |
| ǁ∆ROVVK1PKGeneCatǁ | The Euclidean distance of vitamin K1 Ro5 Violations (Lipinski) in the PKGeneCat group |
| ǁ∆LOGPAntibioticsPKGeneCatǁ | The Euclidean distance of antibiotics (such as cefuroxime, levofloxacin, and azithromycin) CX LogD pH 7.4 in the PKGeneCat group |
| ǁ∆LOGPVnhibitorPKGeneCatǁ | The Euclidean distance of inhibitors (such as amiodarone, fluconazole, and voriconazole) of cytochrome P450 enzyme CX LogD pH 7.4 in the PKGeneCat group |
| ǁ∆LOGPCelecoxibPKGeneCatǁ | The Euclidean distance of celecoxib CX LogD pH 7.4 in the PKGeneCat group |
| ǁ∆LOGPDiltiazemPKGeneCatǁ | The Euclidean distance of diltiazem CX LogD pH 7.4 in the PKGeneCat group |
| ǁ∆LOGPCorticoidsPKGeneCatǁ | The Euclidean distance of corticoids (such as methylprednisolone and prednisone) CX LogD pH 7.4 in the PKGeneCat group |
| ǁ∆LOGPDiureticPKGeneCatǁ | The Euclidean distance of diuretic (such as spironolactone and furosemide) CX LogD pH 7.4 in the PKGeneCat group |
| ǁ∆LOGPOmeprazoPKGeneCatǁ | The Euclidean distance of omeprazole CX LogD pH 7.4 in the PKGeneCat group |
| ǁ∆LOGPAspirinPKGeneCatǁ | The Euclidean distance of aspirin CX LogD pH 7.4 in the PKGeneCat group |
| ǁ∆LOGPStatinPKGeneCatǁ | The Euclidean distance of statin (such as rosuvastatin, atorvastatin, and simvastatin) CX LogD pH 7.4 in the PKGeneCat group |
| ǁ∆LOGPDiclofenacPKGeneCatǁ | The Euclidean distance of diclofenac CX LogD pH 7.4 in the PKGeneCat group |
| ǁ∆LOGPClopidogrelPKGeneCatǁ | The Euclidean distance of clopidogrel CX LogD pH 7.4 in the PKGeneCat group |
| ǁ∆LOGPVK1PKGeneCatǁ | The Euclidean distance of vitamin K1 CX LogD pH 7.4 in the PKGeneCat group |
| ǁ∆Blood_cell_countPKGeneCatǁ | The Euclidean distance of blood cell counts in the PKGeneCat group |
| ǁ∆BiochemistryPKGeneCatǁ | The Euclidean distance of blood biochemistry in the PKGeneCat group |
| ǁ∆VKConPKGeneCatǁ | The Euclidean distance of vitamin K concentration in the PKGeneCat group |
| ǁ∆AntibioticsPKGeneCatǁ | The Euclidean distance of antibiotics (such as vancomycin and teicoplanin) in the PKGeneCat group |
| ǁ∆AlbuminPKGeneCatǁ | The Euclidean distance of human albumin in the PKGeneCat group |
| ǁ∆ProbioticsPKGeneCatǁ | The Euclidean distance of probiotics (such as bifidobacterium triple viable powder, live *Bacillus licheniformis*, and bifidobacterium) in the PKGeneCat group |
| ǁ∆LMWHPKGeneCatǁ | The Euclidean distance of low molecular weight heparin (such as enoxaparin and nadroparin) in the PKGeneCat group |
| ǁ∆DosePDGeneCatǁ | The Euclidean distance of warfarin dosage in the PDGeneCat group |
| ǁ∆DEMOPDGeneCatǁ | The Euclidean distance of continuous demography variables (such as age, height, weight, and postoperative days) in the PDGeneCat group |
| ǁ∆MWAntibioticsPDGeneCatǁ | The Euclidean distance of antibiotics (such as cefuroxime, levofloxacin, and azithromycin) molecular weight in the PDGeneCat group |
| ǁ∆MWInhibitorPDGeneCatǁ | The Euclidean distance of inhibitors (such as amiodarone, fluconazole, and voriconazole) of cytochrome P450 enzyme molecular weight in the PDGeneCat group |
| ǁ∆MWCelecoxibPDGeneCatǁ | The Euclidean distance of celecoxib molecular weight in the PDGeneCat group |
| ǁ∆MWDiltiazemPDGeneCatǁ | The Euclidean distance of diltiazem molecular weight in the PDGeneCat group |
| ǁ∆MWCorticoidsPDGeneCatǁ | The Euclidean distance of corticoids (such as methylprednisolone and prednisone) molecular weight in the PDGeneCat group |
| ǁ∆MWDiureticPDGeneCatǁ | The Euclidean distance of diuretic (such as spironolactone and furosemide) molecular weight in the PDGeneCat group |
| ǁ∆MWOmeprazoPDGeneCatǁ | The Euclidean distance of omeprazole molecular weight in the PDGeneCat group |
| ǁ∆MWAspirinPDGeneCatǁ | The Euclidean distance of aspirin molecular weight in the PDGeneCat group |
| ǁ∆MWStatinPDGeneCatǁ | The Euclidean distance of statin (such as rosuvastatin, atorvastatin, and simvastatin) molecular weight in the PDGeneCat group |
| ǁ∆MWDiclofenacPDGeneCatǁ | The Euclidean distance of diclofenac molecular weight in the PDGeneCat group |
| ǁ∆MWClopidogrelPDGeneCatǁ | The Euclidean distance of clopidogrel molecular weight in the PDGeneCat group |
| ǁ∆MWVK1PDGeneCatǁ | The Euclidean distance of vitamin K1 molecular weight in the PDGeneCat group |
| ǁ∆PSAAntibioticsPDGeneCatǁ | The Euclidean distance of antibiotics (such as cefuroxime, levofloxacin, and azithromycin) polar surface area in the PDGeneCat group |
| ǁ∆PSAInhibitorPDGeneCatǁ | The Euclidean distance of inhibitors (such as amiodarone, fluconazole, and voriconazole) of cytochrome P450 enzyme polar surface area in the PDGeneCat group |
| ǁ∆PSACelecoxibPDGeneCatǁ | The Euclidean distance of celecoxib polar surface area in the PDGeneCat group |
| ǁ∆PSADiltiazemPDGeneCatǁ | The Euclidean distance of diltiazem polar surface area in the PDGeneCat group |
| ǁ∆PSACorticoidsPDGeneCatǁ | The Euclidean distance of corticoids (such as methylprednisolone and prednisone) polar surface area in the PDGeneCat group |
| ǁ∆PSADiureticPDGeneCatǁ | The Euclidean distance of diuretic (such as spironolactone and furosemide) polar surface area in the PDGeneCat group |
| ǁ∆PSAOmeprazoPDGeneCatǁ | The Euclidean distance of omeprazole polar surface area in the PDGeneCat group |
| ǁ∆PSAAspirinPDGeneCatǁ | The Euclidean distance of aspirin polar surface area in the PDGeneCat group |
| ǁ∆PSAStatinPDGeneCatǁ | The Euclidean distance of statin (such as rosuvastatin, atorvastatin, and simvastatin) polar surface area in the PDGeneCat group |
| ǁ∆PSADiclofenacPDGeneCatǁ | The Euclidean distance of diclofenac polar surface area in the PDGeneCat group |
| ǁ∆PSAClopidogrelPDGeneCatǁ | The Euclidean distance of clopidogrel polar surface area in the PDGeneCat group |
| ǁ∆PSAVK1PDGeneCatǁ | The Euclidean distance of vitamin K1 polar surface area in the PDGeneCat group |
| ǁ∆ROVAntibioticsPDGeneCatǁ | The Euclidean distance of antibiotics (such as cefuroxime, levofloxacin, and azithromycin) Ro5 Violations (Lipinski) in the PDGeneCat group |
| ǁ∆ROVInhibitorPDGeneCatǁ | The Euclidean distance of inhibitors (such as amiodarone, fluconazole, and voriconazole) of cytochrome P450 enzyme Ro5 Violations (Lipinski) in the PDGeneCat group |
| ǁ∆ROVStatinPDGeneCatǁ | The Euclidean distance of statin (such as rosuvastatin, atorvastatin, and simvastatin) Ro5 Violations (Lipinski) in the PDGeneCat group |
| ǁ∆ROVVK1PDGeneCatǁ | The Euclidean distance of vitamin K1 Ro5 Violations (Lipinski) in the PDGeneCat group |
| ǁ∆LOGPAntibioticsPDGeneCatǁ | The Euclidean distance of antibiotics (such as cefuroxime, levofloxacin, and azithromycin) CX LogD pH 7.4 in the PDGeneCat group |
| ǁ∆LOGPInhibitorPDGeneCatǁ | The Euclidean distance of inhibitors (such as amiodarone, fluconazole, and voriconazole) of cytochrome P450 enzyme CX LogD pH 7.4 in the PDGeneCat group |
| ǁ∆LOGPCelecoxibPDGeneCatǁ | The Euclidean distance of celecoxib CX LogD pH 7.4 in the PDGeneCat group |
| ǁ∆LOGPDiltiazemPDGeneCatǁ | The Euclidean distance of diltiazem CX LogD pH 7.4 in the PDGeneCat group |
| ǁ∆LOGPCorticoidsPDGeneCatǁ | The Euclidean distance of corticoids (such as methylprednisolone and prednisone) CX LogD pH 7.4 in the PDGeneCat group |
| ǁ∆LOGPDiureticPDGeneCatǁ | The Euclidean distance of diuretic (such as spironolactone and furosemide) CX LogD pH 7.4 in the PDGeneCat group |
| ǁ∆LOGPOmeprazoPDGeneCatǁ | The Euclidean distance of omeprazole CX LogD pH 7.4 in the PDGeneCat group |
| ǁ∆LOGPAspirinPDGeneCatǁ | The Euclidean distance of aspirin CX LogD pH 7.4 in the PDGeneCat group |
| ǁ∆LOGPStatinPDGeneCatǁ | The Euclidean distance of statin (such as rosuvastatin, atorvastatin, and simvastatin) CX LogD pH 7.4 in the PDGeneCat group |
| ǁ∆LOGPDiclofenacPDGeneCatǁ | The Euclidean distance of diclofenac CX LogD pH 7.4 in the PDGeneCat group |
| ǁ∆LOGPClopidogrelPDGeneCatǁ | The Euclidean distance of clopidogrel CX LogD pH 7.4 in the PDGeneCat group |
| ǁ∆LOGPVK1PDGeneCatǁ | The Euclidean distance of vitamin K1 CX LogD pH 7.4 in the PDGeneCat group |
| ǁ∆Blood_cell_countPDGeneCatǁ | The Euclidean distance of blood cell counts in the PDGeneCat group |
| ǁ∆BiochemistryPDGeneCatǁ | The Euclidean distance of blood biochemistry in the PDGeneCat group |
| ǁ∆VKConPDGeneCatǁ | The Euclidean distance of vitamin K concentration in the PDGeneCat group |
| ǁ∆AntibioticsPDGeneCatǁ | The Euclidean distance of antibiotics (such as vancomycin and teicoplanin) in the PDGeneCat group |
| ǁ∆AlbuminPDGeneCatǁ | The Euclidean distance of human albumin in the PDGeneCat group |
| ǁ∆ProbioticsPDGeneCatǁ | The Euclidean distance of probiotics (such as bifidobacterium triple viable powder, live *Bacillus licheniformis*, and bifidobacterium) in the PDGeneCat group |
| ǁ∆LMWHPDGeneCatǁ | The Euclidean distance of low molecular weight heparin (such as enoxaparin and nadroparin) in the PDGeneCat group |
| ǁ∆DoseVKPKGeneCatǁ | The Euclidean distance of warfarin dosage in the VKPKGeneCat group |
| ǁ∆DEMOVKPKGeneCatǁ | The Euclidean distance of continuous demography variables (such as age, height, weight, and postoperative days) in the VKPKGeneCat group |
| ǁ∆MWAntibioticsVKPKGeneCatǁ | The Euclidean distance of antibiotics (such as cefuroxime, levofloxacin, and azithromycin) molecular weight in the VKPKGeneCat group |
| ǁ∆MWInhibitorVKPKGeneCatǁ | The Euclidean distance of inhibitors (such as amiodarone, fluconazole, and voriconazole) of cytochrome P450 enzyme molecular weight in the VKPKGeneCat group |
| ǁ∆MWCelecoxibVKPKGeneCatǁ | The Euclidean distance of celecoxib molecular weight in the VKPKGeneCat group |
| ǁ∆MWDiltiazemVKPKGeneCatǁ | The Euclidean distance of diltiazem molecular weight in the VKPKGeneCat group |
| ǁ∆MWCorticoidsVKPKGeneCatǁ | The Euclidean distance of corticoids (such as methylprednisolone and prednisone) molecular weight in the VKPKGeneCat group |
| ǁ∆MWDiureticVKPKGeneCatǁ | The Euclidean distance of diuretic (such as spironolactone and furosemide) molecular weight in the VKPKGeneCat group |
| ǁ∆MWOmeprazoVKPKGeneCatǁ | The Euclidean distance of omeprazole molecular weight in the VKPKGeneCat group |
| ǁ∆MWAspirinVKPKGeneCatǁ | The Euclidean distance of aspirin molecular weight in the VKPKGeneCat group |
| ǁ∆MWStatinVKPKGeneCatǁ | The Euclidean distance of statin (such as rosuvastatin, atorvastatin, and simvastatin) molecular weight in the VKPKGeneCat group |
| ǁ∆MWDiclofenacVKPKGeneCatǁ | The Euclidean distance of diclofenac molecular weight in the VKPKGeneCat group |
| ǁ∆MWClopidogrelVKPKGeneCatǁ | The Euclidean distance of clopidogrel molecular weight in the VKPKGeneCat group |
| ǁ∆MWVK1VKPKGeneCatǁ | The Euclidean distance of vitamin K1 molecular weight in the VKPKGeneCat group |
| ǁ∆PSAAntibioticsVKPKGeneCatǁ | The Euclidean distance of antibiotics (such as cefuroxime, levofloxacin, and azithromycin) polar surface area in the VKPKGeneCat group |
| ǁ∆PSAInhibitorVKPKGeneCatǁ | The Euclidean distance of inhibitors (such as amiodarone, fluconazole, and voriconazole) of cytochrome P450 enzyme polar surface area in the VKPKGeneCat group |
| ǁ∆PSACelecoxibVKPKGeneCatǁ | The Euclidean distance of celecoxib polar surface area in the VKPKGeneCat group |
| ǁ∆PSADiltiazemVKPKGeneCatǁ | The Euclidean distance of diltiazem polar surface area in the VKPKGeneCat group |
| ǁ∆PSACorticoidsVKPKGeneCatǁ | The Euclidean distance of corticoids (such as methylprednisolone and prednisone) polar surface area in the VKPKGeneCat group |
| ǁ∆PSADiureticVKPKGeneCatǁ | The Euclidean distance of diuretic (such as spironolactone and furosemide) polar surface area in the VKPKGeneCat group |
| ǁ∆PSAOmeprazoVKPKGeneCatǁ | The Euclidean distance of omeprazole polar surface area in the VKPKGeneCat group |
| ǁ∆PSAAspirinVKPKGeneCatǁ | The Euclidean distance of aspirin polar surface area in the VKPKGeneCat group |
| ǁ∆PSAStatinVKPKGeneCatǁ | The Euclidean distance of statin (such as rosuvastatin, atorvastatin, and simvastatin) polar surface area in the VKPKGeneCat group |
| ǁ∆PSADiclofenacVKPKGeneCatǁ | The Euclidean distance of diclofenac polar surface area in the VKPKGeneCat group |
| ǁ∆PSAClopidogrelVKPKGeneCatǁ | The Euclidean distance of clopidogrel polar surface area in the VKPKGeneCat group |
| ǁ∆PSAVK1VKPKGeneCatǁ | The Euclidean distance of vitamin K1 polar surface area in the VKPKGeneCat group |
| ǁ∆ROVAntibioticsVKPKGeneCatǁ | The Euclidean distance of antibiotics (such as cefuroxime, levofloxacin, and azithromycin) Ro5 Violations (Lipinski) in the VKPKGeneCat group |
| ǁ∆ROVInhibitorVKPKGeneCatǁ | The Euclidean distance of inhibitors (such as amiodarone, fluconazole, and voriconazole) of cytochrome P450 enzyme Ro5 Violations (Lipinski) in the VKPKGeneCat group |
| ǁ∆ROVStatinVKPKGeneCatǁ | The Euclidean distance of statin (such as rosuvastatin, atorvastatin, and simvastatin) Ro5 Violations (Lipinski) in the VKPKGeneCat group |
| ǁ∆ROVVK1VKPKGeneCatǁ | The Euclidean distance of vitamin K1 Ro5 Violations (Lipinski) in the VKPKGeneCat group |
| ǁ∆LOGPAntibioticsVKPKGeneCatǁ | The Euclidean distance of antibiotics (such as cefuroxime, levofloxacin, and azithromycin) CX LogD pH 7.4 in the VKPKGeneCat group |
| ǁ∆LOGPInhibitorVKPKGeneCatǁ | The Euclidean distance of inhibitors (such as amiodarone, fluconazole, and voriconazole) of cytochrome P450 enzyme CX LogD pH 7.4 in the VKPKGeneCat group |
| ǁ∆LOGPCelecoxibVKPKGeneCatǁ | The Euclidean distance of celecoxib CX LogD pH 7.4 in the VKPKGeneCat group |
| ǁ∆LOGPDiltiazemVKPKGeneCatǁ | The Euclidean distance of diltiazem CX LogD pH 7.4 in the VKPKGeneCat group |
| ǁ∆LOGPCorticoidsVKPKGeneCatǁ | The Euclidean distance of corticoids (such as methylprednisolone and prednisone) CX LogD pH 7.4 in the VKPKGeneCat group |
| ǁ∆LOGPDiureticVKPKGeneCatǁ | The Euclidean distance of diuretic (such as spironolactone and furosemide) CX LogD pH 7.4 in the VKPKGeneCat group |
| ǁ∆LOGPOmeprazoVKPKGeneCatǁ | The Euclidean distance of omeprazole CX LogD pH 7.4 in the VKPKGeneCat group |
| ǁ∆LOGPAspirinVKPKGeneCatǁ | The Euclidean distance of aspirin CX LogD pH 7.4 in the VKPKGeneCat group |
| ǁ∆LOGPStatinVKPKGeneCatǁ | The Euclidean distance of statin (such as rosuvastatin, atorvastatin, and simvastatin) CX LogD pH 7.4 in the VKPKGeneCat group |
| ǁ∆LOGPDiclofenacVKPKGeneCatǁ | The Euclidean distance of diclofenac CX LogD pH 7.4 in the VKPKGeneCat group |
| ǁ∆LOGPClopidogrelVKPKGeneCatǁ | The Euclidean distance of clopidogrel CX LogD pH 7.4 in the VKPKGeneCat group |
| ǁ∆LOGPVK1VKPKGeneCatǁ | The Euclidean distance of vitamin K1 CX LogD pH 7.4 in the VKPKGeneCat group |
| ǁ∆Blood_cell_countVKPKGeneCatǁ | The Euclidean distance of blood cell counts in the VKPKGeneCat group |
| ǁ∆BiochemistryVKPKGeneCatǁ | The Euclidean distance of blood biochemistry in the VKPKGeneCat group |
| ǁ∆VKConVKPKGeneCatǁ | The Euclidean distance of vitamin K concentration in the VKPKGeneCat group |
| ǁ∆AntibioticsVKPKGeneCatǁ | The Euclidean distance of antibiotics (such as vancomycin and teicoplanin) in the VKPKGeneCat group |
| ǁ∆AlbuminVKPKGeneCatǁ | The Euclidean distance of human albumin in the VKPKGeneCat group |
| ǁ∆ProbioticsVKPKGeneCatǁ | The Euclidean distance of probiotics (such as bifidobacterium triple viable powder, live *Bacillus licheniformis*, and bifidobacterium) in the VKPKGeneCat group |
| ǁ∆LMWHVKPKGeneCatǁ | The Euclidean distance of low molecular weight heparin (such as enoxaparin and nadroparin) in the VKPKGeneCat group |
| ǁ∆DoseNRGeneCatǁ | The Euclidean distance of warfarin dosage in the NRGeneCat group |
| ǁ∆DEMONRGeneCatǁ | The Euclidean distance of continuous demography variables (such as age, height, weight, and postoperative days) in the NRGeneCat group |
| ǁ∆MWAntibioticsNRGeneCatǁ | The Euclidean distance of antibiotics (such as cefuroxime, levofloxacin, and azithromycin) molecular weight in the NRGeneCat group |
| ǁ∆MWInhibitorNRGeneCatǁ | The Euclidean distance of inhibitors (such as amiodarone, fluconazole, and voriconazole) of cytochrome P450 enzyme molecular weight in the NRGeneCat group |
| ǁ∆MWCelecoxibNRGeneCatǁ | The Euclidean distance of celecoxib molecular weight in the NRGeneCat group |
| ǁ∆MWDiltiazemNRGeneCatǁ | The Euclidean distance of diltiazem molecular weight in the NRGeneCat group |
| ǁ∆MWCorticoidsNRGeneCatǁ | The Euclidean distance of corticoids (such as methylprednisolone and prednisone) molecular weight in the NRGeneCat group |
| ǁ∆MWDiureticNRGeneCatǁ | The Euclidean distance of diuretic (such as spironolactone and furosemide) molecular weight in the NRGeneCat group |
| ǁ∆MWOmeprazoNRGeneCatǁ | The Euclidean distance of omeprazole molecular weight in the NRGeneCat group |
| ǁ∆MWAspirinNRGeneCatǁ | The Euclidean distance of aspirin molecular weight in the NRGeneCat group |
| ǁ∆MWStatinNRGeneCatǁ | The Euclidean distance of statin (such as rosuvastatin, atorvastatin, and simvastatin) molecular weight in the NRGeneCat group |
| ǁ∆MWDiclofenacNRGeneCatǁ | The Euclidean distance of diclofenac molecular weight in the NRGeneCat group |
| ǁ∆MWClopidogrelNRGeneCatǁ | The Euclidean distance of clopidogrel molecular weight in the NRGeneCat group |
| ǁ∆MWVK1NRGeneCatǁ | The Euclidean distance of vitamin K1 molecular weight in the NRGeneCat group |
| ǁ∆PSAAntibioticsNRGeneCatǁ | The Euclidean distance of antibiotics (such as cefuroxime, levofloxacin, and azithromycin) polar surface area in the NRGeneCat group |
| ǁ∆PSAInhibitorNRGeneCatǁ | The Euclidean distance of inhibitors (such as amiodarone, fluconazole, and voriconazole) of cytochrome P450 enzyme polar surface area in the NRGeneCat group |
| ǁ∆PSACelecoxibNRGeneCatǁ | The Euclidean distance of celecoxib polar surface area in the NRGeneCat group |
| ǁ∆PSADiltiazemNRGeneCatǁ | The Euclidean distance of diltiazem polar surface area in the NRGeneCat group |
| ǁ∆PSACorticoidsNRGeneCatǁ | The Euclidean distance of corticoids (such as methylprednisolone and prednisone) polar surface area in the NRGeneCat group |
| ǁ∆PSADiureticNRGeneCatǁ | The Euclidean distance of diuretic (such as spironolactone and furosemide) polar surface area in the NRGeneCat group |
| ǁ∆PSAOmeprazoNRGeneCatǁ | The Euclidean distance of omeprazole polar surface area in the NRGeneCat group |
| ǁ∆PSAAspirinNRGeneCatǁ | The Euclidean distance of aspirin polar surface area in the NRGeneCat group |
| ǁ∆PSAStatinNRGeneCatǁ | The Euclidean distance of statin (such as rosuvastatin, atorvastatin, and simvastatin) polar surface area in the NRGeneCat group |
| ǁ∆PSADiclofenacNRGeneCatǁ | The Euclidean distance of diclofenac polar surface area in the NRGeneCat group |
| ǁ∆PSAClopidogrelNRGeneCatǁ | The Euclidean distance of clopidogrel polar surface area in the NRGeneCat group |
| ǁ∆PSAVK1NRGeneCatǁ | The Euclidean distance of vitamin K1 polar surface area in the NRGeneCat group |
| ǁ∆ROVAntibioticsNRGeneCatǁ | The Euclidean distance of antibiotics (such as cefuroxime, levofloxacin, and azithromycin) Ro5 Violations (Lipinski) in the NRGeneCat group |
| ǁ∆ROVInhibitorNRGeneCatǁ | The Euclidean distance of inhibitors (such as amiodarone, fluconazole, and voriconazole) of cytochrome P450 enzyme Ro5 Violations (Lipinski) in the NRGeneCat group |
| ǁ∆ROVStatinNRGeneCatǁ | The Euclidean distance of statin (such as rosuvastatin, atorvastatin, and simvastatin) Ro5 Violations (Lipinski) in the NRGeneCat group |
| ǁ∆ROVVK1NRGeneCatǁ | The Euclidean distance of vitamin K1 Ro5 Violations (Lipinski) in the NRGeneCat group |
| ǁ∆LOGPAntibioticsNRGeneCatǁ | The Euclidean distance of antibiotics (such as cefuroxime, levofloxacin, and azithromycin) CX LogD pH 7.4 in the NRGeneCat group |
| ǁ∆LOGPVnhibitorNRGeneCatǁ | The Euclidean distance of inhibitors (such as amiodarone, fluconazole, and voriconazole) of cytochrome P450 enzyme CX LogD pH 7.4 in the NRGeneCat group |
| ǁ∆LOGPCelecoxibNRGeneCatǁ | The Euclidean distance of celecoxib CX LogD pH 7.4 in the NRGeneCat group |
| ǁ∆LOGPDiltiazemNRGeneCatǁ | The Euclidean distance of diltiazem CX LogD pH 7.4 in the NRGeneCat group |
| ǁ∆LOGPCorticoidsNRGeneCatǁ | The Euclidean distance of corticoids (such as methylprednisolone and prednisone) CX LogD pH 7.4 in the NRGeneCat group |
| ǁ∆LOGPDiureticNRGeneCatǁ | The Euclidean distance of diuretic (such as spironolactone and furosemide) CX LogD pH 7.4 in the NRGeneCat group |
| ǁ∆LOGPOmeprazoNRGeneCatǁ | The Euclidean distance of omeprazole CX LogD pH 7.4 in the NRGeneCat group |
| ǁ∆LOGPAspirinNRGeneCatǁ | The Euclidean distance of aspirin CX LogD pH 7.4 in the NRGeneCat group |
| ǁ∆LOGPStatinNRGeneCatǁ | The Euclidean distance of statin (such as rosuvastatin, atorvastatin, and simvastatin) CX LogD pH 7.4 in NRGeneCat group |
| ǁ∆LOGPDiclofenacNRGeneCatǁ | The Euclidean distance of diclofenac CX LogD pH 7.4 in the NRGeneCat group |
| ǁ∆LOGPClopidogrelNRGeneCatǁ | The Euclidean distance of clopidogrel CX LogD pH 7.4 in the NRGeneCat group |
| ǁ∆LOGPVK1NRGeneCatǁ | The Euclidean distance of vitamin K1 CX LogD pH 7.4 in the NRGeneCat group |
| ǁ∆Blood_cell_countNRGeneCatǁ | The Euclidean distance of blood cell counts in the NRGeneCat group |
| ǁ∆BiochemistryNRGeneCatǁ | The Euclidean distance of blood biochemistry in the NRGeneCat group |
| ǁ∆VKConNRGeneCatǁ | The Euclidean distance of vitamin K concentration in the NRGeneCat group |
| ǁ∆AntibioticsNRGeneCatǁ | The Euclidean distance of antibiotics (such as vancomycin and teicoplanin) in the NRGeneCat group |
| ǁ∆AlbuminNRGeneCatǁ | The Euclidean distance of human albumin in the NRGeneCat group |
| ǁ∆ProbioticsNRGeneCatǁ | The Euclidean distance of probiotics (such as bifidobacterium triple viable powder, live *Bacillus licheniformis*, and bifidobacterium) in the NRGeneCat group |
| ǁ∆LMWHNRGeneCatǁ | The Euclidean distance of low molecular weight heparin (such as enoxaparin and nadroparin) in the NRGeneCat group |
| ǁ∆DoseClotGeneCatǁ | The Euclidean distance of warfarin dosage in the ClotGeneCat group |
| ǁ∆DEMOClotGeneCatǁ | The Euclidean distance of continuous demography variables (such as age, height, weight, and postoperative days) in the ClotGeneCat group |
| ǁ∆MWAntibioticsClotGeneCatǁ | The Euclidean distance of antibiotics (such as cefuroxime, levofloxacin, and azithromycin) molecular weight in the ClotGeneCat group |
| ǁ∆MWInhibitorClotGeneCatǁ | The Euclidean distance of inhibitors (such as amiodarone, fluconazole, and voriconazole) of cytochrome P450 enzyme molecular weight in the ClotGeneCat group |
| ǁ∆MWCelecoxibClotGeneCatǁ | The Euclidean distance of celecoxib molecular weight in the ClotGeneCat group |
| ǁ∆MWDiltiazemClotGeneCatǁ | The Euclidean distance of diltiazem molecular weight in the ClotGeneCat group |
| ǁ∆MWCorticoidsClotGeneCatǁ | The Euclidean distance of corticoids (such as methylprednisolone and prednisone) molecular weight in the ClotGeneCat group |
| ǁ∆MWDiureticClotGeneCatǁ | The Euclidean distance of diuretic (such as spironolactone and furosemide) molecular weight in the ClotGeneCat group |
| ǁ∆MWOmeprazoClotGeneCatǁ | The Euclidean distance of omeprazole molecular weight in the ClotGeneCat group |
| ǁ∆MWAspirinClotGeneCatǁ | The Euclidean distance of aspirin molecular weight in the ClotGeneCat group |
| ǁ∆MWStatinClotGeneCatǁ | The Euclidean distance of statin (such as rosuvastatin, atorvastatin, and simvastatin) molecular weight in the ClotGeneCat group |
| ǁ∆MWDiclofenacClotGeneCatǁ | The Euclidean distance of diclofenac molecular weight in the ClotGeneCat group |
| ǁ∆MWClopidogrelClotGeneCatǁ | The Euclidean distance of clopidogrel molecular weight in the ClotGeneCat group |
| ǁ∆MWVK1ClotGeneCatǁ | The Euclidean distance of vitamin K1 molecular weight in the ClotGeneCat group |
| ǁ∆PSAAntibioticsClotGeneCatǁ | The Euclidean distance of antibiotics (such as cefuroxime, levofloxacin, and azithromycin) polar surface area in the ClotGeneCat group |
| ǁ∆PSAInhibitorClotGeneCatǁ | The Euclidean distance of inhibitors (such as amiodarone, fluconazole, and voriconazole) of cytochrome P450 enzyme polar surface area in the ClotGeneCat group |
| ǁ∆PSACelecoxibClotGeneCatǁ | The Euclidean distance of celecoxib polar surface area in the ClotGeneCat group |
| ǁ∆PSADiltiazemClotGeneCatǁ | The Euclidean distance of diltiazem polar surface area in the ClotGeneCat group |
| ǁ∆PSACorticoidsClotGeneCatǁ | The Euclidean distance of corticoids (such as methylprednisolone and prednisone) polar surface area in the ClotGeneCat group |
| ǁ∆PSADiureticClotGeneCatǁ | The Euclidean distance of diuretic (such as spironolactone and furosemide) polar surface area in the ClotGeneCat group |
| ǁ∆PSAOmeprazoClotGeneCatǁ | The Euclidean distance of omeprazole polar surface area in the ClotGeneCat group |
| ǁ∆PSAAspirinClotGeneCatǁ | The Euclidean distance of aspirin polar surface area in the ClotGeneCat group |
| ǁ∆PSAStatinClotGeneCatǁ | The Euclidean distance of statin (such as rosuvastatin, atorvastatin, and simvastatin) polar surface area in the ClotGeneCat group |
| ǁ∆PSADiclofenacClotGeneCatǁ | The Euclidean distance of diclofenac polar surface area in the ClotGeneCat group |
| ǁ∆PSAClopidogrelClotGeneCatǁ | The Euclidean distance of clopidogrel polar surface area in the ClotGeneCat group |
| ǁ∆PSAVK1ClotGeneCatǁ | The Euclidean distance of vitamin K1 polar surface area in the ClotGeneCat group |
| ǁ∆ROVAntibioticsClotGeneCatǁ | The Euclidean distance of antibiotics (such as cefuroxime, levofloxacin, and azithromycin) Ro5 Violations (Lipinski) in the ClotGeneCat group |
| ǁ∆ROVInhibitorClotGeneCatǁ | The Euclidean distance of inhibitors (such as amiodarone, fluconazole, and voriconazole) of cytochrome P450 enzyme Ro5 Violations (Lipinski) in the ClotGeneCat group |
| ǁ∆ROVStatinClotGeneCatǁ | The Euclidean distance of statin (such as rosuvastatin, atorvastatin, and simvastatin) Ro5 Violations (Lipinski) in the ClotGeneCat group |
| ǁ∆ROVVK1ClotGeneCatǁ | The Euclidean distance of vitamin K1 Ro5 Violations (Lipinski) in the ClotGeneCat group |
| ǁ∆LOGPAntibioticsClotGeneCatǁ | The Euclidean distance of antibiotics (such as cefuroxime, levofloxacin, and azithromycin) CX LogD pH 7.4 in the ClotGeneCat group |
| ǁ∆LOGPInhibitorClotGeneCatǁ | The Euclidean distance of inhibitors (such as amiodarone, fluconazole, and voriconazole) of cytochrome P450 enzyme CX LogD pH 7.4 in the ClotGeneCat group |
| ǁ∆LOGPCelecoxibClotGeneCatǁ | The Euclidean distance of celecoxib CX LogD pH 7.4 in the ClotGeneCat group |
| ǁ∆LOGPDiltiazemClotGeneCatǁ | The Euclidean distance of diltiazem CX LogD pH 7.4 in the ClotGeneCat group |
| ǁ∆LOGPCorticoidsClotGeneCatǁ | The Euclidean distance of corticoids (such as methylprednisolone and prednisone) CX LogD pH 7.4 in the ClotGeneCat group |
| ǁ∆LOGPDiureticClotGeneCatǁ | The Euclidean distance of diuretic (such as spironolactone and furosemide) CX LogD pH 7.4 in the ClotGeneCat group |
| ǁ∆LOGPOmeprazoClotGeneCatǁ | The Euclidean distance of omeprazole CX LogD pH 7.4 in the ClotGeneCat group |
| ǁ∆LOGPAspirinClotGeneCatǁ | The Euclidean distance of aspirin CX LogD pH 7.4 in the ClotGeneCat group |
| ǁ∆LOGPStatinClotGeneCatǁ | The Euclidean distance of statin (such as rosuvastatin, atorvastatin, and simvastatin) CX LogD pH 7.4 in the ClotGeneCat group |
| ǁ∆LOGPDiclofenacClotGeneCatǁ | The Euclidean distance of diclofenac CX LogD pH 7.4 in the ClotGeneCat group |
| ǁ∆LOGPClopidogrelClotGeneCatǁ | The Euclidean distance of clopidogrel CX LogD pH 7.4 in the ClotGeneCat group |
| ǁ∆LOGPVK1ClotGeneCatǁ | The Euclidean distance of vitamin K1 CX LogD pH 7.4 in the ClotGeneCat group |
| ǁ∆Blood_cell_countClotGeneCatǁ | The Euclidean distance of blood cell counts in the ClotGeneCat group |
| ǁ∆BiochemistryClotGeneCatǁ | The Euclidean distance of blood biochemistry in the ClotGeneCat group |
| ǁ∆VKConClotGeneCatǁ | The Euclidean distance of vitamin K concentration in the ClotGeneCat group |
| ǁ∆AntibioticsClotGeneCatǁ | The Euclidean distance of antibiotics (such as vancomycin and teicoplanin) in the ClotGeneCat group |
| ǁ∆AlbuminClotGeneCatǁ | The Euclidean distance of human albumin in the ClotGeneCat group |
| ǁ∆ProbioticsClotGeneCatǁ | The Euclidean distance of probiotics (such as bifidobacterium triple viable powder, live *Bacillus licheniformis*, and bifidobacterium) in the ClotGeneCat group |
| ǁ∆LMWHClotGeneCatǁ | The Euclidean distance of low molecular weight heparin (such as enoxaparin and nadroparin) in the ClotGeneCat group |


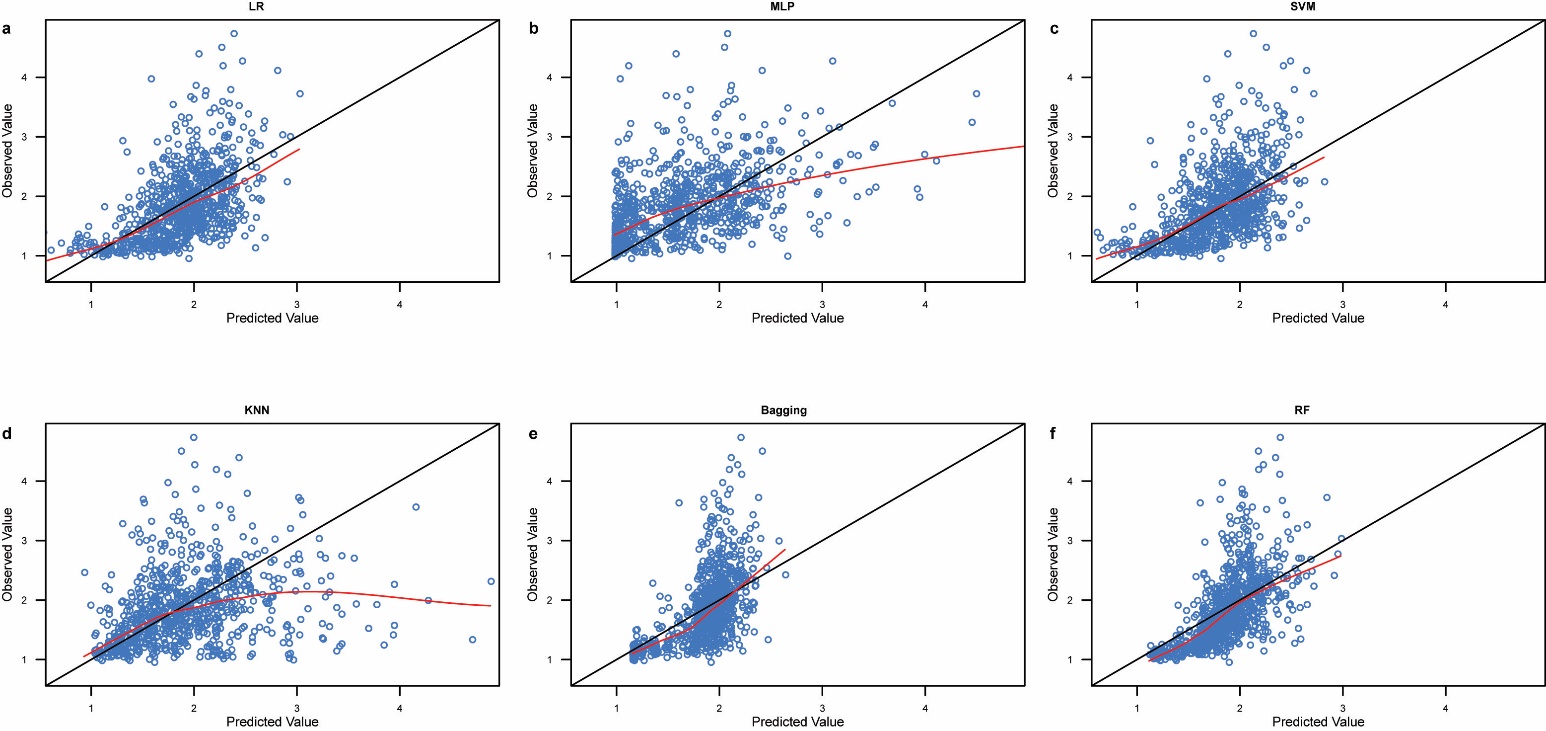


**Fig. S1**. Scatter plot of the observed values and model-predicted values in the validation datasets with the raw clinical longitudinal data.


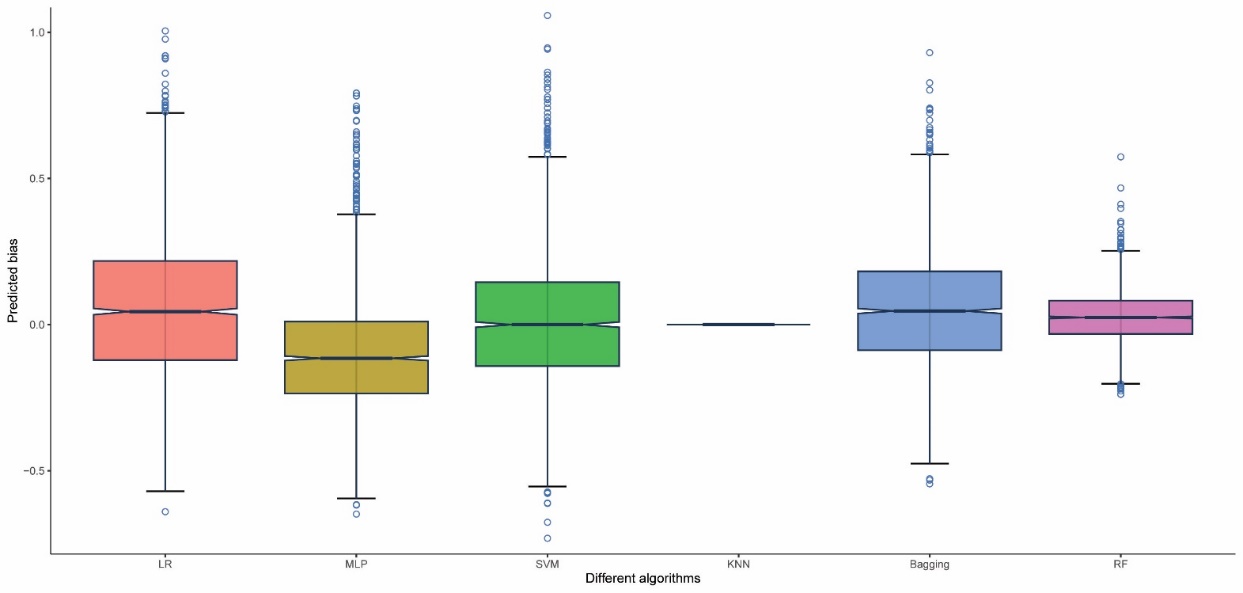


**Fig. S2**. The prediction bias of different algorithms in the training datasets with raw clinical longitudinal data.

**Fig. S3**. The prediction bias of different algorithms in the validation datasets with the raw clinical longitudinal data.

**
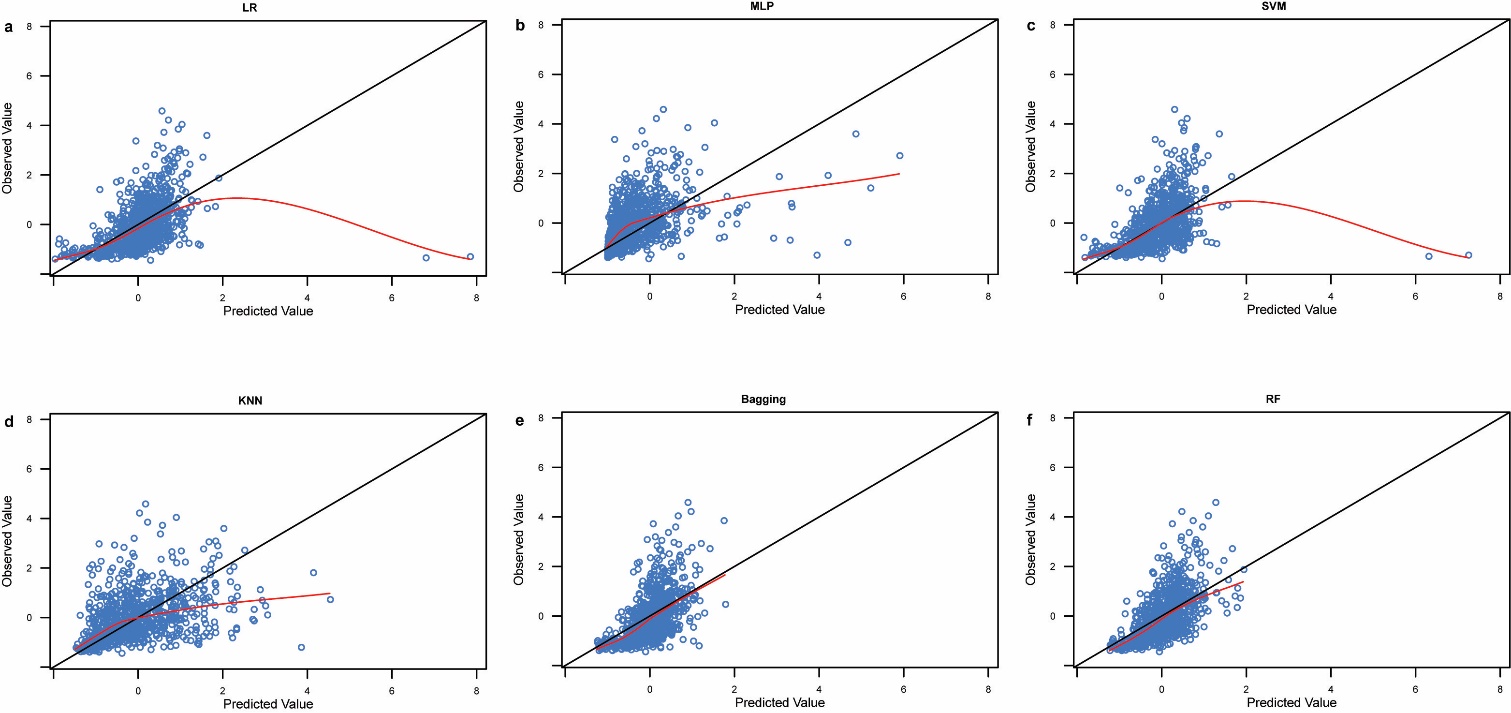
**

**Fig. S4**. Scatterplot of the observed values and model-predicted values in the validation dataset with moving average ∆v_k_(**s**_j_) data


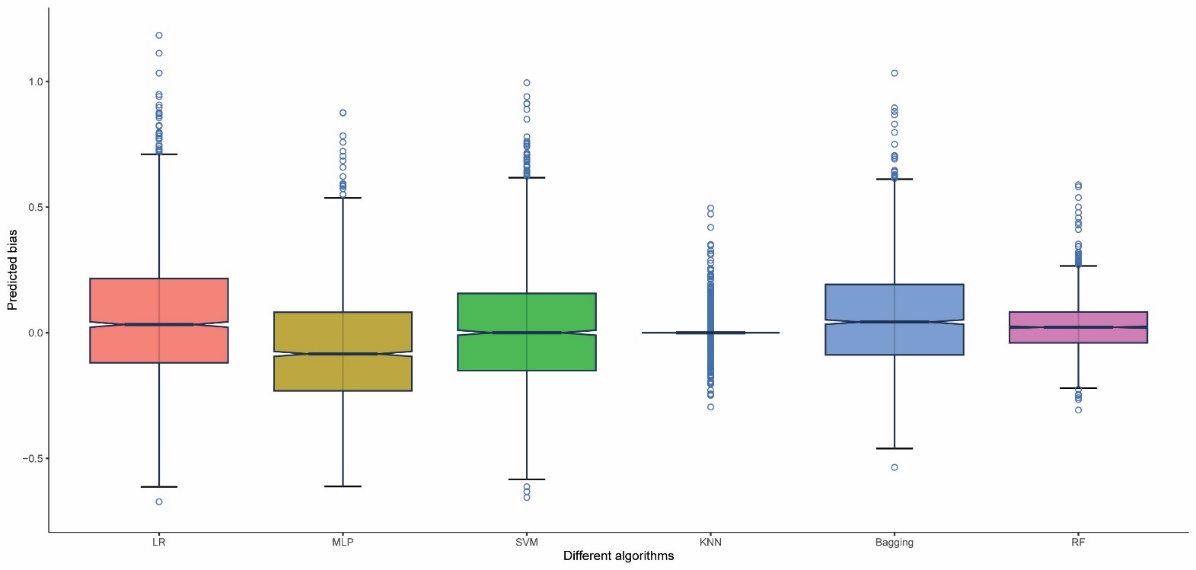


**Fig. S5**. The prediction bias of different algorithms in the training datasets with moving average ∆v_ki_(**s**_j_) data.

**Fig. S6**. The prediction bias of different algorithms in the validation dataset with moving average ∆v_k_(**s**_j_) data


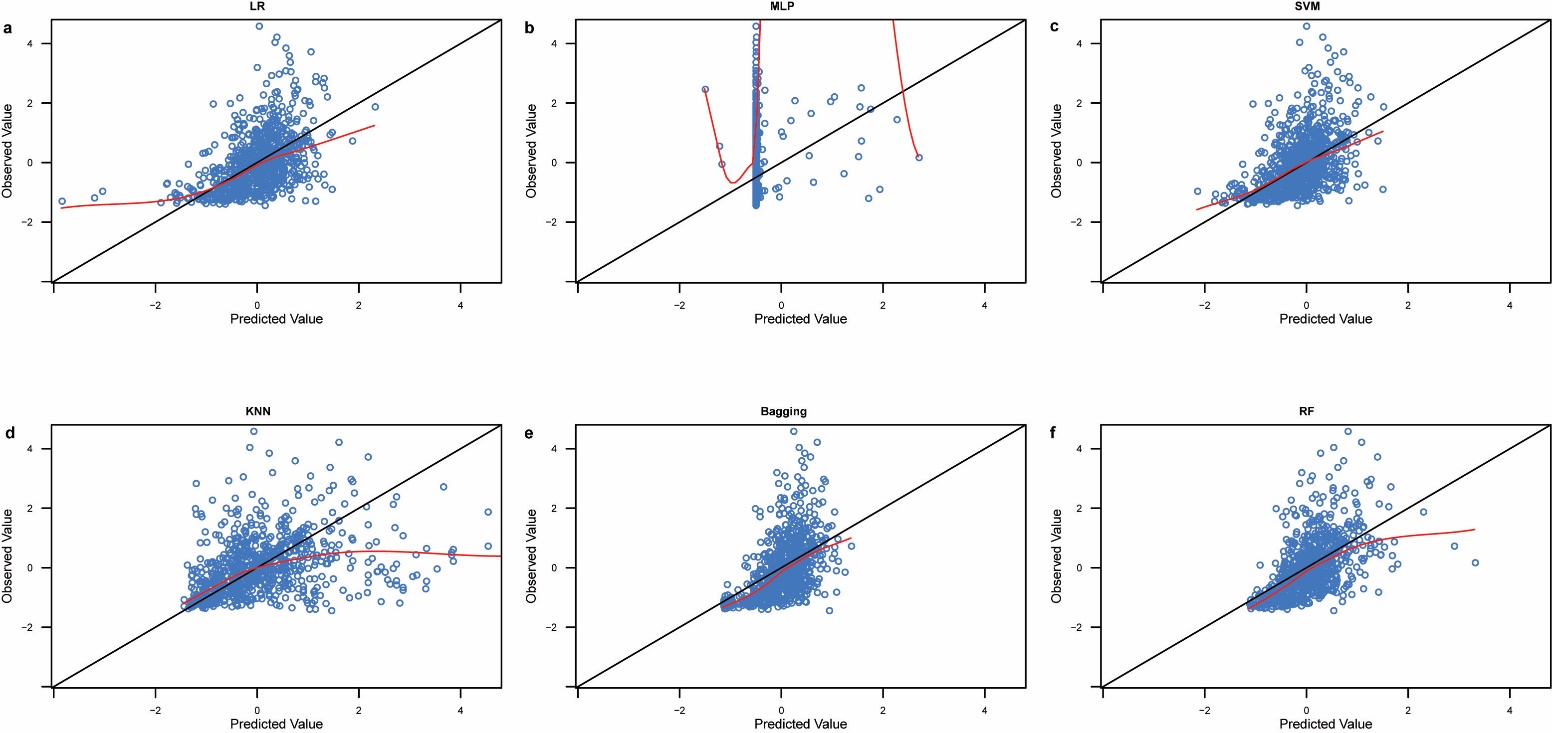


**Fig. S7**. Scatter plot of the observed values and model-predicted values in the validation datasets with Euclidean distance ǁ∆v_k_(**s**_j_)ǁ data.


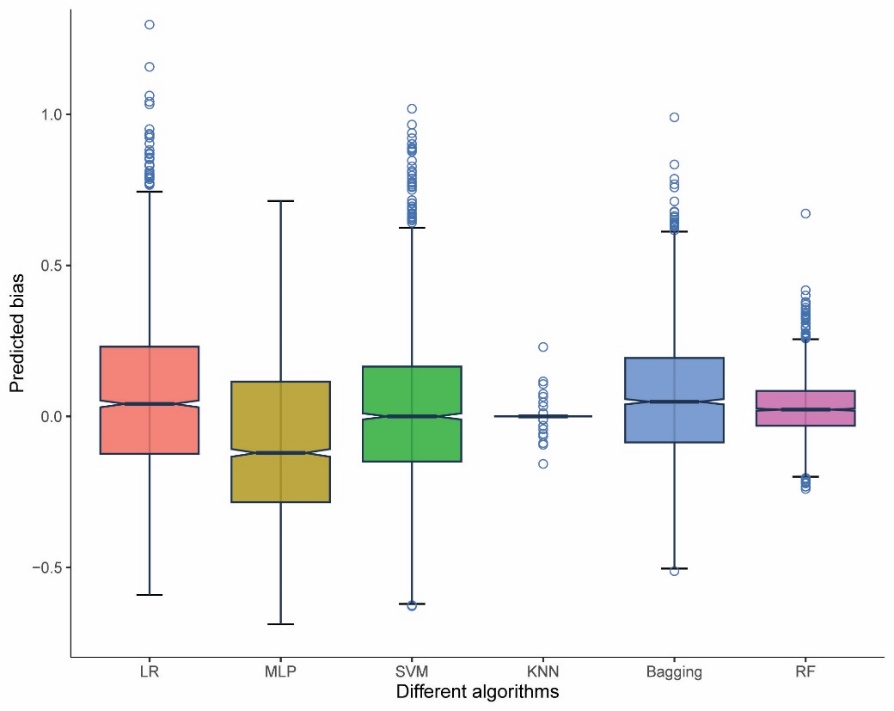


**Fig. S8**. The prediction bias of different algorithms in training datasets with Euclidean distance ǁ∆v_k_(**s**_j_)ǁ data.

**Fig. S9**. The prediction bias of different algorithms in the validation datasets with Euclidean distance ǁ∆v_k_(**s**_j_)ǁ data.
